# EraSOR: a software tool to eliminate inflation caused by sample overlap in polygenic score analyses

**DOI:** 10.1093/gigascience/giad043

**Published:** 2023-06-16

**Authors:** Shing Wan Choi, Timothy Shin Heng Mak, Clive J Hoggart, Paul F O'Reilly

**Affiliations:** Department of Genetics and Genomic Sciences, Icahn School of Medicine, Mount Sinai, New York City, NY 10029, USA; MRC Social, Genetic and Developmental Psychiatry Centre, Institute of Psychiatry, Psychology and Neuroscience, King's College London, London, SE5 8AF, UK; Centre of Genomic Sciences, University of Hong Kong, Pokfulam, Hong Kong SAR, China; Department of Genetics and Genomic Sciences, Icahn School of Medicine, Mount Sinai, New York City, NY 10029, USA; Department of Genetics and Genomic Sciences, Icahn School of Medicine, Mount Sinai, New York City, NY 10029, USA; MRC Social, Genetic and Developmental Psychiatry Centre, Institute of Psychiatry, Psychology and Neuroscience, King's College London, London, SE5 8AF, UK

## Abstract

**Background:**

Polygenic risk score (PRS) analyses are now routinely applied across biomedical research. However, as PRS studies grow in size, there is an increased risk of sample overlap between the genome-wide association study (GWAS) from which the PRS is derived and the “target sample,” in which PRSs are computed and hypotheses are tested. Despite the wide recognition of the sample overlap problem, its potential impact on the results from PRS studies has not yet been quantified, and no analytical solution has been provided.

**Findings:**

Here, we first conduct a comprehensive investigation into the scale of the sample overlap problem, finding that PRS results can be substantially inflated even in the presence of minimal overlap. Next, we introduce a method and software, EraSOR (Erase Sample Overlap and Relatedness), which eliminates the inflation caused by sample overlap (and close relatedness) in almost all settings tested here.

**Conclusions:**

EraSOR could be useful in PRS studies (with target sample >1,000) similar to those investigated here, either (i) to mitigate the potential effects of known or unknown intercohort overlap and close relatedness or (ii) as a sensitivity tool to highlight the possible presence of sample overlap before its direct removal, when possible, or else to provide a lower bound on PRS analysis results after accounting for potential sample overlap.

## Introduction

Polygenic risk scores (PRSs) are proxies of individuals’ genetic liability to a trait or disease [1] that have been applied in a range of research settings, including patient stratification [2] and investigation of treatment response [3–6]. The power of PRS analyses that test a study hypothesis is dependent on the heritability and polygenicity of the trait, the power of the genome-wide association study (GWAS) used to derive the PRS, and the size of the target data sample used to test the hypothesis [7]. The recent surge in availability of high-quality genotype–phenotype data from large-scale biobank projects, such as the UK Biobank [8], BioBank Japan [9], Taiwan Biobank [10], and FinnGen [11], as well as GWAS resources from large consortia such as the Psychiatric Genomic Consortium [12], GIANT [13], and the Global Lipids Genetics Consortium (GLGC) [14], have provided unprecedented opportunities to perform highly powered PRS analyses.

However, expansion in data sizes comes with a cost in this setting: as sample sizes increase, it is more likely that samples are recruited into multiple cohorts or that entire cohorts are included in multiple consortia. For PRS analyses, which typically test for association between PRS and a trait(s) or outcome of interest, overlapping samples between the GWAS and target data samples can result in spurious inflation of the coefficient of determination (*R*^2^) and association *P* values, leading to false-positive and exaggerated findings [15]. If an entire cohort is present in the base GWAS and target data, then ideally it should be removed as follows: (i) directly remove the cohort from the GWAS data (recompute base GWAS results); (ii) if (i) is not an option, then use the cohort GWAS results to derive the base GWAS results minus the cohort, an analytical solution of which we have previously described; and (iii) remove cohort from the target data directly if doing so does not compromise power. Likewise, overlapping individuals should ideally be removed from either the GWAS or target data to avoid misinterpretation of results, but participant privacy agreements usually limit access to raw genotyping data, meaning that this is generally not an option.

Here we first evaluate the extent to which different degrees of sample overlap and relatedness between GWAS and target samples generate biased PRS–trait associations. Next, to overcome the sample overlap problem, we develop and introduce EraSOR (Erase Sample Overlap and Relatedness), a software that adjusts GWAS summary statistics [1] to correct for inflation of PRS–trait association results caused by overlapping samples between the GWAS and target samples. Through extensive simulations using the UK Biobank genetic data [8], we demonstrate that EraSOR can robustly adjust for inflation in test statistics caused by various degrees of overlapping samples and close relatedness, under different ascertainment schemes in case/control settings. EraSOR should increase the accuracy of results in all future PRS studies with known sample overlap and will act as a critical sensitivity tool as part of PRS analyses to ensure the reliability of results in PRS studies with unknown but potential sample overlap. EraSOR is an open-source software and is freely available [16].

## Methods

### EraSOR framework

Consider 2 GWASs *k* = {1, 2} performed on the same continuous outcome *Y_k_*. The effect size of the *g*th single-nucleotide polymorphism (SNP) in study *k* (${\beta }_{kg}$) is estimated using a regression model


(1) \begin{eqnarray*}
{Y}_k = {\alpha }_{kg}{\mathrm{\ }} + {\beta }_{kg}{X}_{kg} + {\varepsilon }_{kg}
\end{eqnarray*}

where ${X}_{kg}$ is the standardized genotype vector for SNP *g* in study *k*, and ${\varepsilon }_{kg}$ is the random error assumed to be independent between studies. Under the null model of no contribution of SNP *g* to the trait, ${\beta }_{kg} = \ 0$, and assuming no sample overlap, then $\widehat {{\beta }_{1g}}$ and $\widehat {{\beta }_{2g}}$ estimated from the 2 GWASs should be independent (i.e., $cor\ ( {\widehat {{\beta }_{1g}},\widehat {{\beta }_{2g}}} ) = \ 0$). However, when there are overlapping samples between the 2 studies, then a correlation is induced between the regression coefficients, such that $cor( {\widehat {{\beta }_{1g}},\widehat {{\beta }_{2g}}} ) \ne 0$. From LeBlanc et al. [17], this correlation can be approximated as


(2) \begin{eqnarray*}
cor\left( {\widehat {{\beta }_{1g}},\widehat {{\beta }_{2g}}} \right) \approx \frac{{{N}_c}}{{\sqrt {{N}_1{N}_2} }}cor\left( {{Y}_1,{\mathrm{\ }}{Y}_2} \right) \end{eqnarray*}

for quantitative traits, where $cor( {{Y}_1,\ {Y}_2} )$ represents the correlation between the traits, ${N}_c$ is the number of overlapping samples, and ${N}_1$, ${N}_2$ are the sample sizes of studies 1 and 2, respectively [17]. Since we are considering only a single phenotype here, $cor( {{Y}_1,\ {Y}_2} )$ is equal to 1, and so we have


(3) \begin{eqnarray*}
cor\left( {\widehat {{\beta }_{1g}},\widehat {{\beta }_{2g}}} \right) \approx \frac{{{N}_c}}{{\sqrt {{N}_1{N}_2} }}
\end{eqnarray*}

which captures correlations due only to sample overlap, independent of the true causal effect (note that if the cohorts were also identical, then N_1_ = N_2_ = N_c_, and thus, appropriately, both sides of (3) would equal 1). Assuming sample overlap does not affect the standard error estimates, LeBlanc et al. [17] proposed that when the number of overlapping samples (N_c_) is known, one can adjust the joint distribution of the summary statistics (*z*-scores) of the 2 GWASs as


(4) \begin{eqnarray*}
{{\boldsymbol{z}}}_{{\boldsymbol{de}} - {\boldsymbol{corr}}} = {{\boldsymbol{C}}}^{ - 0.5}{\boldsymbol{\ z}}
\end{eqnarray*}

where ***z*** is a 2-by-*M* matrix containing *z*-scores estimated in each study, *M* is the number of SNPs common to both studies, and ***C*** is the 2 × 2 matrix with 1s as its diagonal elements and $cor( {\widehat {{\beta }_{1g}},\widehat {{\beta }_{2g}}} )$ as its off-diagonal elements. While this adjustment is effective [17], it requires prior knowledge of N_c_, which is typically unknown in PRS studies. Here, we propose utilizing univariate and bivariate Linkage Disequilibrium (LD) score regression [18, 19] to estimate $\frac{{{N}_c}}{{\sqrt {{N}_1{N}_2} }}$ and thus $cor( {\widehat {{\beta }_{1g}},\widehat {{\beta }_{2g}}} )$ from Equation 3 as follows described below.

Bivariate LD score regression [19] is typically used to estimate the genetic correlation between 2 traits using two GWASs corresponding to each. In the formulation of bivariate LD score regression, genetic stratification—which corresponds to structure in genetic variation in a population due to nonrandom mating—is assumed to be similar in each GWAS sample, since making this assumption simplifies the mathematics and is approximately true in many settings. Yengo et al. [13] generalized this equation by introducing Wright's ${F}_{ST}$, which measures genetic stratification, and made the simplifying assumption that genetic structure in a population is caused by the population comprising 2 subpopulations, with mating occurring mostly within, rather than between, the subpopulations. In this way, *F_ST_* here measures the genetic differences between 2 subpopulations that make up each study population, and the *F_ST_* within each study should be the same. Yengo et al. further introduced an environmental stratification term, ${\sigma }_S,$ which is the mean phenotypic difference between the subpopulations. This leads to the following equation:


(5) \begin{eqnarray*}
\mathbb{E}\ \left[ {{z}_{1j}{z}_{2j}} \right] = \frac{{\sqrt {{N}_1{N}_2} {\rho }_g}}{M}\ {l}_j + \frac{{{N}_c\rho }}{{\sqrt {{N}_1{N}_2} }} + {\rho }_gF_{ST}^2\sqrt {{N}_1{N}_2} + \frac{{N_c^2{F}_{ST}\sigma _S^2}}{{\sqrt {{N}_1{N}_2} }}
\end{eqnarray*}

where ${l}_j$ is the “LD score,” defined as the sum of pairwise squared correlations between genotypes at SNP *j* and all other SNPs within the same LD block; ${\rho }_g$ is the genetic covariance between the 2 traits; $\rho \ = \ {\rho }_g + \ {\rho }_e$; ${\rho }_e$ is the nongenetic covariance; and ${F}_{ST}$ and ${\sigma }_s$ are the genetic and environmental stratification, respectively [13]. Since we are considering only a single phenotype here, $\rho $ is equal to 1, and so we have


(6) \begin{eqnarray*}
\mathbb{E}\ \left[ {{z}_{1j}{z}_{2j}} \right] = \frac{{\sqrt {{N}_1{N}_2} {\rho }_g}}{M}\ {l}_j + \frac{{{N}_c}}{{\sqrt {{N}_1{N}_2} }} + {\rho }_gF_{ST}^2\sqrt {{N}_1{N}_2} + \frac{{N_c^2{F}_{ST}\sigma _S^2}}{{\sqrt {{N}_1{N}_2} }}
\end{eqnarray*}

We wish to solve for N_c_ and hence apply Equation 3 to generate a de-correlated base GWAS that does not lead to inflated PRS–trait associations due to sample overlap. To do this, we will utilize the univariate LD score regression model, which can be derived as a special case of the bivariate LD score equation by assuming that the 2 outcomes and cohorts are identical [13, 18] (i.e., ${N}_1 = {N}_2\ = {N}_c\ = \ N$), leading to


(7) \begin{eqnarray*}
\mathbb{E}\ \left[ {\chi _j^2} \right] = \frac{{N{h}^2}}{M}\ {l}_j + 1 + N{F}_{ST}\left( {{h}^2{F}_{ST} + \sigma _s^2} \right) \end{eqnarray*}

Univariate LD score regression performs a regression of observed ${{\mathrm{\chi }}}^2$ on ${l}_j$, with the effect size estimate of *l_j_* corresponding to a scaled estimate of heritability ($\widehat {h_i^2}\ $) and with the estimated intercept term $\widehat {{I}_u}$ as follows:


\begin{eqnarray*}
{\mathrm{\ }}\widehat {{I}_u} = \ 1 + {N}_i{F}_{ST}\left( {\widehat {h_i^2}{F}_{ST} + {\mathrm{\sigma }}_S^2} \right) \end{eqnarray*}

A key observation by Yengo et al. [13] is that in addition to the level of sample overlap, the inflation of the bivariate LD score regression intercept is also affected by the level of genetic and environmental stratification. As such, the bivariate LD score regression intercept cannot directly be used as an estimate of the level of sample overlap. However, if we assume that the environmental stratification ${\mathrm{\sigma }}_S^2 = \ 0$, then we have


(8) \begin{eqnarray*} \begin{array}{@{}*{1}{l}@{}} {\widehat {{I}_u} = 1 + {N}_i{F}_{ST}\left( {\widehat {h_i^2}{F}_{ST} + {\mathrm{\sigma }}_S^2} \right)}\\ {\widehat {{I}_u} = 1 + {N}_iF_{ST}^2\widehat {h_i^2}}\\ {F_{ST}^2 = \frac{{\widehat {{I}_u} - 1}}{{{N}_i\widehat {h_i^2}}}} \end{array}
\end{eqnarray*}

Since we can estimate $F_{ST}^2$ using both the base and target data, we can take the weighted mean estimate of both:


(9) \begin{eqnarray*}
\widehat {F_{ST}^2} = \frac{1}{{{N}_1 + {N}_2}}\ \mathop \sum \nolimits_{i\ = {\mathrm{\ }}1}^2 \frac{{\widehat {{I}_i} - 1}}{{\widehat {h_i^2}}}
\end{eqnarray*}

The intercept term of the bivariate LD score regression is


(10) \begin{eqnarray*}
\widehat {{I}_b} = \frac{{{N}_c}}{{\sqrt {{N}_1{N}_2} }}{\mathrm{\ }} + \widehat {{{\mathrm{\rho }}}_g}F_{ST}^2\sqrt {{N}_1{N}_2} + \frac{{N_c^2{F}_{ST}{\mathrm{\sigma }}_S^2}}{{\sqrt {{N}_1{N}_2} }}
\end{eqnarray*}

Substituting Equation 9 and $\sigma _S^2 = \ 0$ into Equation 10, we have


(11) \begin{eqnarray*} \begin{array}{@{}*{1}{l}@{}} {\widehat {{I}_b} = \frac{{{N}_c}}{{\sqrt {{N}_1{N}_2} }} + \widehat {{{\mathrm{\rho }}}_g}\widehat {F_{ST}^2}\sqrt {{N}_1{N}_2} + \frac{{N_c^2{F}_{ST}{\mathrm{\sigma }}_S^2}}{{\sqrt {{N}_1{N}_2} }}}\\ {\widehat {{I}_b} = \frac{{{N}_c}}{{\sqrt {{N}_1{N}_2} }} + \widehat {{{\mathrm{\rho }}}_g}\sqrt {{N}_1{N}_2} \left( {\frac{1}{{{N}_1 + {N}_2}}\mathop \sum \nolimits_{i = 1}^2 \frac{{\widehat {{I}_i} - 1}}{{\widehat {h_i^2}}}} \right)}\\ {\frac{{{N}_c}}{{\sqrt {{N}_1{N}_2} }}\& = \frac{{\widehat {{{\mathrm{\rho }}}_g}\sqrt {{N}_1{N}_2} }}{{{N}_1 + {N}_2}}\mathop \sum \nolimits_{i = 1}^2 \frac{{\widehat {{I}_i} - 1}}{{\widehat {h_i^2}}} - \widehat {{I}_b}} \end{array}
\end{eqnarray*}

Since we can estimate the genetic covariate ($\widehat {{{\mathrm{\rho }}}_g}$), the trait heritability $\widehat {h_i^2}$, and the intercepts from the univariate and bivariate LD score regression analyses of the GWAS and target data, we can obtain an estimate of $\frac{{{N}_c}}{{\sqrt {{N}_1{N}_2} }}$. Substituting this estimate into Equation 3 will derive an estimate of $cor( {\widehat {{\beta }_{1g}},\widehat {{\beta }_{2g}}} )$ that can be used to produce de-correlated GWAS *z*-statistics via Equation 4. EraSOR automatically performs the bivariate LD score and univariate LD score regression analyses on the GWAS summary statistics generated from the base and target data. Because of our assumption of ${\mathrm{\sigma }}_S^2 = \ 0$, EraSOR may underperform when large environmental stratification is present. In addition, Equation 2 models the inflation under the null. For highly polygenic traits, a high fraction of variants across the genome may deviate from the null, thus introducing bias into EraSOR adjustments. To test the performance of EraSOR, including its robustness to the modeling assumptions (e.g., assuming ${\mathrm{\sigma }}_S^2 = \ 0$), we performed a series of extensive simulations.

### UK Biobank genotype data

The UK Biobank is a prospective cohort study of around 500,000 individuals recruited across the United Kingdom during 2006–2010. The genetic data from the UK Biobank comprises 488,377 samples and 805,426 SNPs. Standard quality control (QC) procedures were performed, removing any SNPs with minor allele frequency <0.01, genotype missingness >0.02, and a Hardy–Weinberg equilibrium test *P* < 1 × 10^−8^. Samples with high levels of missingness or heterozygosity, with mismatching genetic-inferred and self-reported sex, or with aneuploidy of the sex chromosomes were removed as recommended by the UK Biobank data processing team. Next, 4-means clustering was applied to the first 2 principal components (PCs) of the genotype data, and those individuals in the (largest) cluster corresponding to European ancestry were retained for the primary analyses because polygenic risk scores have been shown to have low portability between ancestries [14], motivating ancestry-matched PRS studies until cross-ancestry PRS methods are developed, which our main results correspond to (see Samples with Population Stratification section, which describes analyses that we also performed on individuals of all ancestries in the UK Biobank). A greedy algorithm [20] was then used to remove related individuals, taking advantage of precomputed pairwise kinship coefficients provided by the UK Biobank. The algorithm first ranks individuals by their number of related pairs (kinship coefficient >0.044), then removes the individual with the highest number of related pairs within the data, which should eliminate a high degree of relatedness while retaining a high sample size. When precomputed kinship coefficients are unavailable, plink2–king cutoff can be used to achieve relatedness removal similarly. In our simulations that investigate the effect of related individuals in the GWAS and target data, we instead randomly retain 1 first-degree relative (defined as kinship coefficient $\ge$0.177 and $\le$0.354) of a randomly sampled individual in the GWAS data. Altogether, we retain 557,369 SNPs, 387,392 individuals, and 23,429 of their first-degree relatives for the set of analyses performed. For the simulations of population-stratified samples, we extracted samples 10 standard deviations from the centroid of the European cluster and defined these as “non-European” samples. QC procedures were repeated using the parameters described above after combining these non-European samples with the European samples, resulting in 387,365 samples of European ancestry and 21,779 individuals of non-European ancestry. Code used to perform the QC and corresponding documentation are available online [21]. This research has been conducted using the UK Biobank Resource under application 18,177 (Dr. O'Reilly).

### Phenotype simulation

#### Quantitative traits without population structure

Quantitative phenotypes (*Y*) with heritability (*h^2^*) of 0, 0.1, and 0.5 were simulated using the UK Biobank genotype data (post-QC; see above) as input. Quantitative traits were simulated as


(12) \begin{eqnarray*}
Y\ = \left( {\alpha + X\beta + \varepsilon } \right)\ \delta
\end{eqnarray*}

where *X* is the standardized genotype matrix corresponding to all samples and the *β* vector corresponds to the effect size associated with each SNP, with either 10,000 or 100,000 SNPs randomly selected to be causal with effect size $\beta \sim N( {0,\ 1} )$, *β* = 0 otherwise. *Xβ* was adjusted such that it has mean 0 and variance *h*^2^, and *ε* represents the random error, which follows $\varepsilon \sim N( {0,\ \sqrt {1 - {h}^2} } )$. To ensure that EraSOR can be applied to traits that do not only follow a strict standard normal distribution (N∼(0,1)), we included α as an intercept parameter (α ∼ N(0,1)) and δ as a scaling parameter (δ ∼ U(1,100)). This was intended only as a basic test of robustness to deviations from a standard normal distribution, and we did not consider any more complex non-Gaussian trait distributions. Consequently, an assumption of EraSOR is that it is applied to continuous data that are either normally distributed or are normalized, using, for example, a log transformation or inverse normal transformation, as is standard in linear regression on continuous outcomes.

To model polygenic risk score analyses with sample overlap, we randomly selected either 120,000 or 250,000 individuals from the sample of 387,392 individuals available to us (see above) to generate 2 different sizes of base GWAS data. Next, we randomly sampled 1,000, 5,000, 10,000, or 50,000 individuals from the remaining sample to act as 3 different sizes of target data, of which 0%, 5%, 10%, 50%, or 100% were randomly selected from the base data sample so that there was a known degree of sample overlap between the base and target data. In addition, we generated an “overlap-free” base cohort in which the overlapping samples were removed from the base cohort so that we could compare the result of applying EraSOR against results of physically removing overlapped samples from the base cohort.

In order to search a feasible parameter space in sufficient depth, we only simulate phenotype with a heritability of 0.5, with a base cohort of 250,000 and a target cohort of 5,000; only simulate a base cohort with 120,000 samples when the phenotypic heritability is ≤0.1 and target cohort has 5,000 samples; and only simulate a target cohort with 1,000 and 10,000 samples when the base cohort contains 250,000 samples and the phenotypic heritability is ≤0.1. The entire set of simulations was repeated 100 times.

#### Binary trait

Binary traits were simulated under the liability threshold model [22], simulating a normally distributed liability using Equation 12 with α = 0, δ = 1, and cases defined as samples with disease liability higher than liability thresholds of 0.9, 0.7, and 0.5, corresponding to population prevalences of 0.1, 0.3, and 0.5, respectively. To limit the complexity of our simulations, the sample prevalence of our cohorts follows the population prevalence, and we only simulated 10,000 causal variants.

In the binary trait setting, overlap can be ascertained such that the overlap is among cases, among controls, or among both. To investigate the effect of case-only or control-only overlap, we randomly selected 120,000 effective samples (effective samples defined as ${N}_{eff} = 4/( {1/{N}_{cases} + 1/{N}_{controls}} )\ $ [23]) as the base cohort and then randomly selected 5,000 effective samples as the target cohort, where 0%, 5%, 10%, 30%, or 50% of the cases or of the controls in the target cohort were sampled from the base cohort. We also performed simulations where the overlapping samples were selected at random among cases and controls. An “overlap-free” base cohort was generated with all overlapping samples removed.

In order to search a feasible parameter space in sufficient depth, we only varied the trait heritability when the population prevalence was 0.1 and only varied the population prevalence when the trait heritability was ≤0.1. These simulations were repeated 100 times.

### Related samples

Spurious inflation in PRS analysis test statistics may also be observed when there are closely related individuals between the base and target cohorts. To investigate the effects of relatedness on PRS results, we repeated the quantitative trait simulations with a modified Equation 12:


(13) \begin{eqnarray*}
Y\ = \left( {\alpha + X\beta + \theta + \varepsilon } \right)\ \delta
\end{eqnarray*}

where $\theta $ is the shared environment between the related individuals and follows a random normal distribution with mean 0 and variance ${\mathrm{\sigma }}_{\mathrm{\theta }}^2 \in ( {0,\ 0.3,\ 0.6} )$ if and only if ${\mathrm{\sigma }}_{\mathrm{\theta }}^2 + {h}^2 < 1$, with each related pair of individuals having the same $\theta $ value. Here, *ε* represents a combination of nonshared environment and random error, which follows $\varepsilon \sim N( {0,\ \sqrt {1 - {h}^2 - {\mathrm{\sigma }}_{\mathrm{\theta }}^2} } )$. To reduce the computational burden, only quantitative traits with 10,000 causal variants were simulated. Additionally, to model the intercohort relatedness, we first selected all individuals with a first-degree relative in the UK Biobank (kinship coefficient $\ge$ 0.177 and $\le$0.354), of which there were 23,429 individuals, and then randomly selected additional samples who did not have any first-degree relatives to form a base cohort containing 250,000 samples. We then generated target cohorts containing 5,000 samples, with 0%, 30%, 60%, or 100% of the target samples being first-degree relatives of samples in the base cohort. We also generated a reference cohort from the base cohort where all the related samples in the target cohort were replaced by unrelated individuals for benchmarking the performance of EraSOR. The entire set of simulations was repeated 100 times.

### Samples with population stratification

An assumption of the EraSOR algorithm is that the environmental stratification (${\mathrm{\sigma }}_s^2$) is zero. When environmental stratification is present, $\frac{{N_c^2{F}_{ST}{\mathrm{\sigma }}_S^2}}{{\sqrt {{N}_1{N}_2} }}$ from Equation 10 is no longer 0 and a bias proportional to the environmental stratification and the genetic stratification (${F}_{ST}$) may be introduced. We partitioned the UK Biobank into European and non-European ancestries and simulated environmental stratifications to test the sensitivity of EraSOR to deviations of each from 0.

UK Biobank samples were divided into European and non-European ancestries based on 4-mean clustering on PC1 and PC2 (see above). Quantitative traits with environmental stratification were then simulated as


(14) \begin{eqnarray*}
Y\ = \left( {{\mathrm{\alpha }} + {\mathrm{X\beta }} + {\mathrm{S}} + \epsilon {\mathrm{\ }}} \right)\ \delta
\end{eqnarray*}

with the environmental stratification term (S) defined as


\begin{eqnarray*}
S\ = \left\{ {\begin{array}{@{}*{1}{c}@{}} { - \frac{{{\sigma }_S}}{2},\ Non - European\ Ancestry}\\ {\frac{{{\sigma }_S}}{2},\ European\ Ancestry} \end{array}} \right.\
\end{eqnarray*}

where ${\mathrm{\sigma }}_S^2$ can take a value of 0, 0.3, or 0.9 if and only if ${\mathrm{\sigma }}_S^2 + {h}^2 < 1$, and $\epsilon $ represents the residual term, which follows $\varepsilon \sim N( {0,\ \sqrt {var( {X\beta + S - 2cov( {X\beta ,\ S} )} )\ \frac{{1 - {h}^2 - \sigma _S^2}}{{{h}^2 + \sigma _S^2}}} } )$, with ${\mathrm{cov}}( {{\mathrm{X\beta }},{\mathrm{\ S}}} )$ being the covariance between ${\mathrm{X\beta }}$ and S. To investigate the effect of sample overlap in the presence of environmental and genetic stratification, we randomly selected either 120,000 or 250,000 individuals from the sample of 409,144 individuals available to us (see above) to generate 2 different sizes of base GWAS data. Next, we randomly sampled 5,000 or 10,000 individuals from the remaining sample to act as 2 different sizes of target data, of which 0%, 10%, 50%, or 100% were randomly selected from the base data sample. To ensure that the genetic and environmental stratification was the same within the base and target data, the same ancestry ratio was maintained in all simulated datasets, matching the ratio in the full dataset (∼5% non-European ancestry). In addition, we generated an “overlap-free” base cohort in which the overlapping samples were removed from the base cohort to allow benchmarking the performance of EraSOR. The entire set of simulations was repeated 50 times.

### Real UK biobank phenotype analysis

To investigate the effect of sample overlap in real phenotypic data, we extracted body mass index (BMI, field ID 21,001), height (field ID 50), and low-density lipoprotein (LDL, field ID 30,780) from the UK Biobank. We residualized the phenotypes against age (field ID 21,003), sex (field ID 31), genotyping batch, UK Biobank assessment center (field ID 54), and 40 PCs. For LDL, we additionally removed individuals who were on statin medication (see Supplementary Materials) and additionally included fasting time (field ID: 74) and dilution factor (field ID: 30,897) as covariates. The residuals were standardized and used as a phenotype for downstream analysis.

To model polygenic risk score analyses with sample overlap, we randomly sampled two-thirds of the individuals with phenotypic information to generate the base GWAS data. Next, we randomly sampled 5,000, 10,000, 5,000, and one-third of the individuals with phenotypic information from the remaining sample to act as the target data, of which 0%, 5%, 10%, 50%, 60%, 70%, 80%, 90%, or 100% were randomly selected from the base data sample so that there was a known degree of sample overlap between the base and target data. In addition, we generated an “overlap-free” base cohort in which the overlapping samples were removed from the base cohort so that we could compare the results of applying EraSOR with the results of direct removal of overlapping samples from the base cohort. The entire set of simulations was repeated 50 times.

In addition, we utilized the latest GWAS summary statistics on LDL from the Global Lipids Genetics Consortium (GLGC) [24] to evaluate the performance of EraSOR. The GLGC has made available summary statistics with or without the inclusion of UK Biobank samples, as well as ancestry-specific and trans-ancestry summary statistics. We generated PRSs using the GLGC summary statistics with UK Biobank samples excluded, representing the “overlap-free” cohort, to calculate the expected performance of PRSs. We then compared the performance of the EraSOR-adjusted and unadjusted PRSs calculated using the UK Biobank-included GLGC summary statistics against the expected performance.

### Genome-wide association study and polygenic score analysis

GWASs were performed on the base and target cohorts using PLINK 2.0 (version 2021–08-04) [25] with the *–glm* function. As binary traits were simulated only for the European ancestry–only analyses, where population structure was not simulated, and considering the computational cost of including covariates in the logistic regression, we did not include PCs in our binary trait analysis. On the other hand, quantitative traits were simulated in all scenarios, some of which were population stratified. Thus, we included 15 PCs as a covariate for our quantitative trait analyses. The resulting summary statistics were then provided to EraSOR to generate the adjusted summary statistics using European LD scores [18] calculated from 1,000 Genomes Project Phase 3 data [26]. PRS analyses using the adjusted, unadjusted, and the “overlap-free” summary statistics were performed using PRSice-2 (v2.3.5) [27] with the default settings. The *R*^2^ and *P* value of association of the PRS–trait tests were reported.

### Strategy for benchmarking

To investigate the level of spurious inflation caused by intercohort relatedness and overlapped samples, we first established a baseline PRS *R*^2^, calculated using base cohorts without overlapped samples. The bias could then be measured as the observed PRS *R*^2^ minuses the baseline PRS *R*^2^ ($\Delta {R}^2$), given the same phenotype and cohort sizes. For nonheritable traits, we also measured the level of false positives, defined as any PRS with *P* < 1 × 10^−4^ [28].

On the other hand, to compare the performance of EraSOR with the optimal strategy of directly removing overlapping samples—an option that is typically not available—we calculated PRSs (i) using summary statistics adjusted by EraSOR (“adjusted PRS”) and (ii) using summary statistics generated from a base cohort with all overlapping and/or related samples removed (“overlap-free PRS”). We present the performance of EraSOR as the PRS–trait association *R*^2^ of the adjusted PRS minuses the *R*^2^ of the overlap-free PRS $( {\Delta {R}^2} )$. If EraSOR has successfully corrected for the sample overlap, then $\Delta {R}^2$ should be close to 0.

## Results

### Inflation caused by overlap

The presence of overlapping samples between the base and target datasets is known to cause inflated association between PRSs and phenotypes, but the extent and characteristics of the problem have not been described. Here, we performed extensive simulations using the UK Biobank [8] genotype data to investigate the inflation caused by different levels and types of intercohort sample overlap in relation to traits simulated with varying heritability and prevalence (see Methods). Base and target cohorts were generated with varying degrees of sample overlap, measured as $\frac{{{N}_c}}{{\sqrt {{N}_1{N}_2} }}$, where ${N}_c$ is the number of overlapping samples and ${N}_1$ and ${N}_2$ are the sample sizes of the base and target cohort, respectively. PRS analyses were conducted using the standard *clumping+thresholding* (C+T) PRS calculation method [1], implemented in *PRSice* [27, 28].

**Figure 1:**
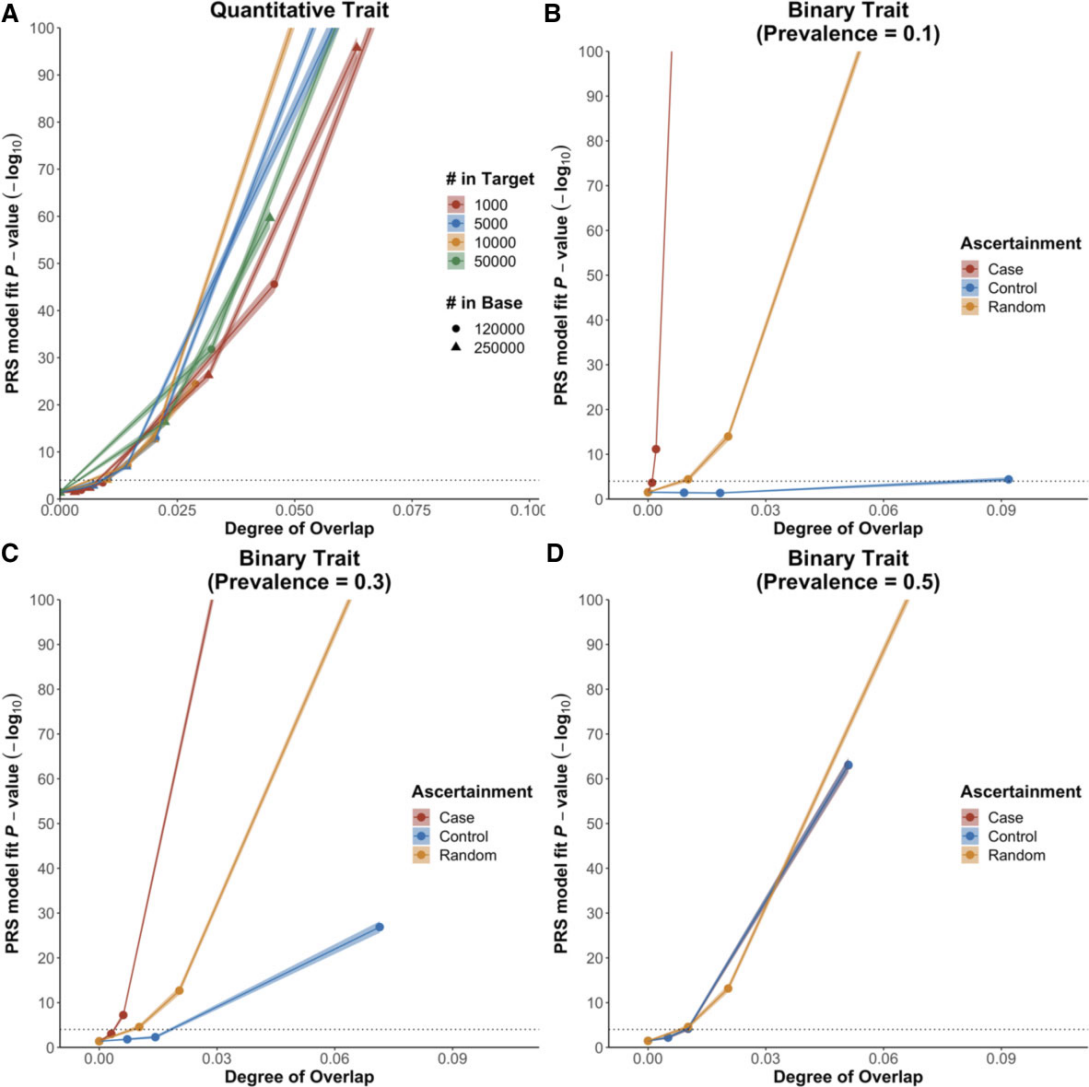
Effect of sample overlap on performance of PRS for nonheritable traits. The dotted line represents the significance threshold (*P* = 1 × 10^−4^) for high-resolution testing in *PRSice-2* [27]. The x-axis shows the degree of overlap, calculated as $\frac{{{N}_c}}{{\sqrt {{N}_1{N}_2} }}$, and the y-axis shows the −log_10_ transformed *P* value of association between the PRS and the simulated phenotype. Shaded area represents the 95% confidence interval. However, as the confidence interval is small, it is difficult to observe on the scale of these plots (A) quantitative traits with different cohort sizes, (b) binary traits with population prevalence of 0.1, (C) binary traits with population prevalence of 0.3, and (D) binary traits with population prevalence of 0.5.

We first estimated the false-positive rate induced by sample overlap by simulating nonheritable traits and recording the fraction of significant PRS–trait association (Supplementary Fig. S1). Highly significant associations between PRS and nonheritable phenotypes were observed when even limited intercohort sample overlap was present (Fig. 1). Specifically, for nonheritable quantitative traits, the inflation in association (e.g., *P* value of association) was highly positively correlated with the degree of overlap (Pearson correlation coefficient $( \gamma )$ = 0.96, *P* < 2.2 × 10^−16^). For example, when there was a base cohort of 250,000 samples, target cohort of 5,000 samples, and 250 overlapping samples (5% of target sample; degree of overlap = 0.0071), the false-positive rate was 16%, while this increased to 90% when there were 500 overlapping samples (10% of target sample; degree of overlap = 0.014) (Fig. 1A).

In the binary trait setting, sample overlap may be among cases only, controls only, or among both. These alternatives were investigated by first simulating binary traits with different population prevalence using the liability threshold model [22]. Cohorts with effective sample sizes of 120,000 in the base data and 5,000 in the target data were generated with different degrees and scenarios of sample overlap. We observed extreme inflation associated with case-only overlap when population prevalence was lower than 0.5. For a binary trait with population prevalence 0.1, a false-positive rate of 40% was observed when the degree of overlap was 0.001, which corresponds to 5% of the cases from the target cohort also present in the base cohort (Fig. 1B). When the degree of sample overlap was doubled (∼0.002), the false-positive rate was 100%. The inflation in PRS–trait association was not as sensitive to the control-only sample overlap when the population prevalence was small. We observed a false-positive rate of 50% when the degree of overlap was as high as 0.092, which corresponds to 50% of the controls from the target cohort also present in the base cohort (Fig. 1B). This discrepancy between the effect of case and control overlap is a result of the differential contribution of cases and controls to the PRS–trait association in our simulations. Cases were sampled from the extreme upper tail of the liability distribution at a frequency corresponding to the disease prevalence, which was typically low: this gives each case greater weight in the calculation of the PRS–trait association and, thus, an overlapping case will generate greater inflation than an overlapping control. This was consistent with our simulation results (Fig. 1C, D), where the inflation in $\Delta {R}^2$ caused by overlapping cases decreased as population prevalence increased ($\gamma \ = \ - 0.245$, *P* = 1.18 × 10^−9^). The reverse relationship between inflation and population prevalence was observed for control-only overlap ($\gamma \ = \ 0.359,\ $  *P* = 1.18 × 10^−19^). For a population prevalence of 0.5, case-only and control-only overlap had the same impact on the inflation (Fig. 1D).

Given that complete overlap of individuals in the base and target data can generate PRS–trait associations that are severely inflated, closely related individuals independently enrolled into the base and target cohorts may induce some inflation considering their shared genetics and environment. Here we tested the effect of relatedness between the base and target cohorts on PRS–trait associations in nonheritable traits in a similar way to that for sample overlap (see Methods), where intercohort relatedness is defined as $\frac{{{N}_r}}{{\sqrt {{N}_1{N}_2} }}$, where ${N}_r$ is the number of samples in the target cohort that are first-degree relatives with samples in the base cohort. A false-positive rate of 100% was observed when the intercohort relatedness was 0.042 (250,000 base, 5,000 target, 30% of target samples had first-degree relatives in the base), when the shared environment explained 30% of the trait variance. See Supplementary Fig. S2 for full results regarding the effects of intercohort relatedness on PRS–trait association inflation.

The analyses in this section were performed only to highlight the potential impact of sample overlap, since highly significant PRS–trait associations were observed with overlap even for nonheritable traits, for which there should be no PRS–trait association. EraSOR should not be applied to data on traits for which there is little evidence of heritability, since PRS analyses performed on underpowered GWAS are more likely to generate misleading results and conclusions based on them. Therefore, as for PRS analyses in general [1], we do not recommend the application of EraSOR to base GWAS data with estimated *h*^2^_SNP_ <5%.

In the next section, we extend these investigations to consider the effects of sample overlap on PRS–trait associations on heritable traits, but we present these findings in conjunction with results based on the application of our method EraSOR, which is designed to resolve the problem.

### Performance of EraSOR

To tackle the problem of inflation caused by inter-cohort overlap and relatedness, we developed the EraSOR method. Using GWAS summary statistics generated from the base and target cohorts, EraSOR implements univariate and bivariate LD score regression [18, 19] to estimate several parameters that are then used to perform a de-correlation calculation of the base GWAS test statistics (see Methods). These adjusted base GWAS summary statistics can then be used for downstream PRS analyses, with sample overlap or relatedness corrected for.

In order to evaluate the performance of EraSOR, we conducted an extensive set of simulations covering a range of scenarios of intercohort sample overlap and relatedness (see below and Methods).

#### Simulations using UK Biobank data

**Figure 2:**
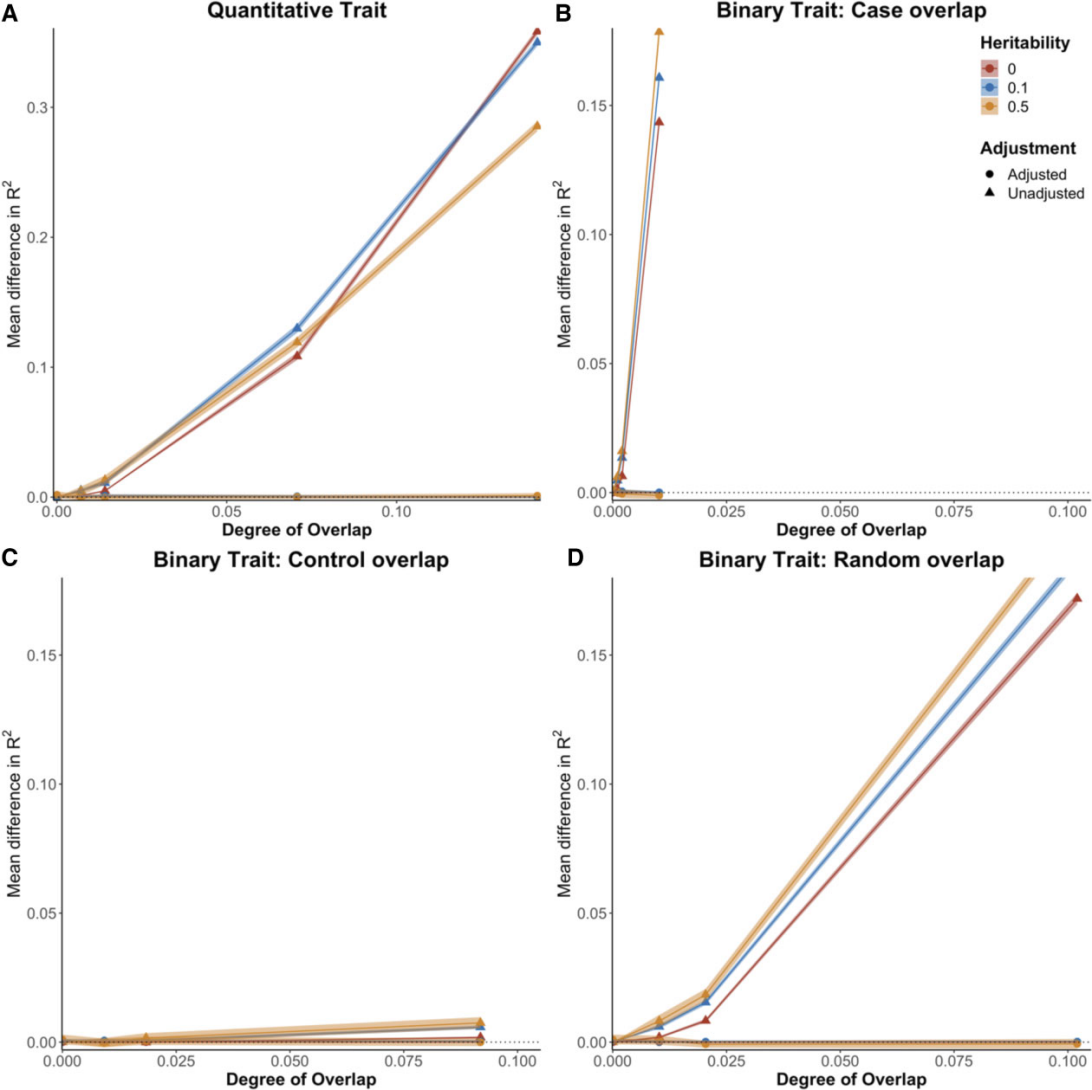
Comparing the performance of the PRS using the EraSOR-adjusted summary statistics and the unadjusted summary statistics. The x-axis shows the degree of overlap, and the y-axis shows the mean difference between the observed *R*^2^ and the expected *R*^2^. Shaded area represents the 95% confidence interval (small on this scale). (A) Performance in quantitative traits with 250,000 samples in the base cohort and 5,000 samples in the target cohort. Performance in binary traits with prevalence of 0.1 and where overlap samples were (B) ascertained for cases, (C) ascertained for controls, (D) or randomly ascertained.

We observed that for both quantitative and binary traits, EraSOR almost entirely eliminates the inflation caused by intercohort overlap and relatedness in our simulations based on UK Biobank (European ancestry base and target samples) data (Fig. 2). These simulations modeled a range of scenarios that varied trait heritability, prevalence, degree of overlap, and combinations of overlap among cases and controls. For example, simulating quantitative traits with a heritability of 0.1, a base cohort of 250,000 samples, a target cohort of 5,000 samples, and degree of overlap of 0.141—in which all samples in the target data are also in the base GWAS—the mean ${\boldsymbol{\Delta }}{{\boldsymbol{R}}}^2{\boldsymbol{\ }}$ is 1.68 × 10^−5^ (standard error: 3.83 × 10^−4^) when there are 10,000 causal variants and 2.21 × 10^−^^4^ (standard error: 4.77 × 10^−^^4^) when there are 100,000 causal variants. Left unadjusted, the mean ${\boldsymbol{\Delta }}{{\boldsymbol{R}}}^2$ is approximately 0.35 (standard error 0.00143) and 0.36 (standard error: 0.00141), respectively, suggesting that EraSOR has removed the inflation introduced by sample overlap. A similar pattern of complete removal of the effects of sample overlap is observed for the quantitative traits across the full range of heritability and cohort sample sizes tested (Fig. 2A), with the exception that when the target cohort is of similar size to the base cohort and when majority of the target samples are found in the base GWAS, EraSOR-adjusted PRS results can deviate from the truth. For example, simulating quantitative traits with a heritability of 0.1, a base cohort size of 120,000, a target cohort of 50,0000 samples, and when all samples in the target are found in the base GWAS, the mean ${\boldsymbol{\Delta }}{{\boldsymbol{R}}}^2$ for the adjusted PRS can be as high as 0.0134 (standard error = 3.73 × 10^−4^), which is still far closer to the truth than the unadjusted PRS (mean ${\boldsymbol{\Delta }}{{\boldsymbol{R}}}^2$ of 0.537). We argue that in such scenarios, while EraSOR cannot completely remove the biases, it can still be used as a tool for sensitivity analyses, where a large discrepancy in performance between the adjusted and unadjusted PRS suggests there might be a large degree of overlap. Similar performances were also observed when lassosum (v0.4.5) [29] instead of PRSice-2 was used for PRS calculation (see Supplementary Fig. S3).

EraSOR also performs extremely well for binary traits in our simulations (Fig. 2B–D). In binary traits with a heritability of 0.1, a population prevalence of 0.1, a base cohort of with 120,000 effective samples, a target cohort of 5,000 effective samples, and with 50% of the target data presented in the base GWAS, the mean ${\mathrm{\Delta }}{R}^2$ for the adjusted PRS in relation to case-only overlap is 7.52 × 10^−5^ (standard error = 2.76 × 10^−4^) (Fig. 2B), and the mean ${\mathrm{\Delta }}{R}^2$ for the adjusted PRS in relation to control-only overlap is 3.36 × 10^−4^ (standard error = 2.84 × 10^−4^) (Fig. 2C). On the other hand, when unadjusted, the mean ${\mathrm{\Delta }}{R}^2$ in relation to case-only overlap is as high as 0.161 (standard error = 0.00110), whereas the mean ${\mathrm{\Delta }}{R}^2$ is 5.80 × 10^−3^ (standard error = 3.48 × 10^−4^) in relation to control-only overlap.

While EraSOR effectively eliminates inflation caused by intercohort overlap in all simulation scenarios tested in relation to heritable traits, false-positive results are still observed after EraSOR adjustment in nonheritable traits when there is a large degree of overlap (>0.289). For nonheritable quantitative traits with base cohorts of 120,000 samples and target cohorts of 10,000 samples, if all target samples are also present in the base cohort, then we observe a false-positive rate of 20%, with a mean ${\mathrm{\Delta }}{R}^2$ of 5.84 × 10^−4^ (standard error = 1.03 × 10^−4^). This is likely caused by the fact that a key component of the mathematics underlying the EraSOR algorithm (described by Equation 11 in Methods) includes an estimate of *h*^2^ in its denominator. Therefore, when the trait is nonheritable, Equation 9 may be unstable and lead to an error in the EraSOR adjustment. However, we recommend that polygenic risk score analyses should not be performed on traits with estimated *h*^2^ <0.05 (see [1]), and thus, in sufficiently powered applications of PRS, EraSOR should have strong performance.

One of the main assumptions of EraSOR is that there is no environmental stratification (${\mathrm{\sigma }}_s^2\ = {\mathrm{\ }}0)$. To investigate the robustness of EraSOR to model misspecification, we also performed simulations by incorporating UK Biobank samples with non-European ancestry and simulated a different level of environmental stratification.

Overall, EraSOR appears to be reasonably robust to model misspecification. For example, considering the high *F_ST_* between the European and non-European samples (see Methods) in UK Biobank (*F_ST_* = 0.018), coupled with a high environmental stratification (e.g., ${\mathrm{\sigma }}_s^2\ = {\mathrm{\ }}$0.3), the mean ${\mathrm{\Delta }}{R}^2$ is still 5.58 × 10^−6^ with standard error of 9.11 × 10^−4^, for quantitative traits with a heritability of 0.1, a base cohort of 250,000 samples, a target cohort of 5,000 samples, and a degree of overlap of 0.141. EraSOR performs equally well for quantitative traits with different heritability, different levels of environmental stratifications, and cohorts with different sample size and overlap (see Supplementary Figs. S4–S6). All simulation results are provided in Supplementary Table S1.

#### Real UK Biobank phenotype analysis

**Figure 3:**
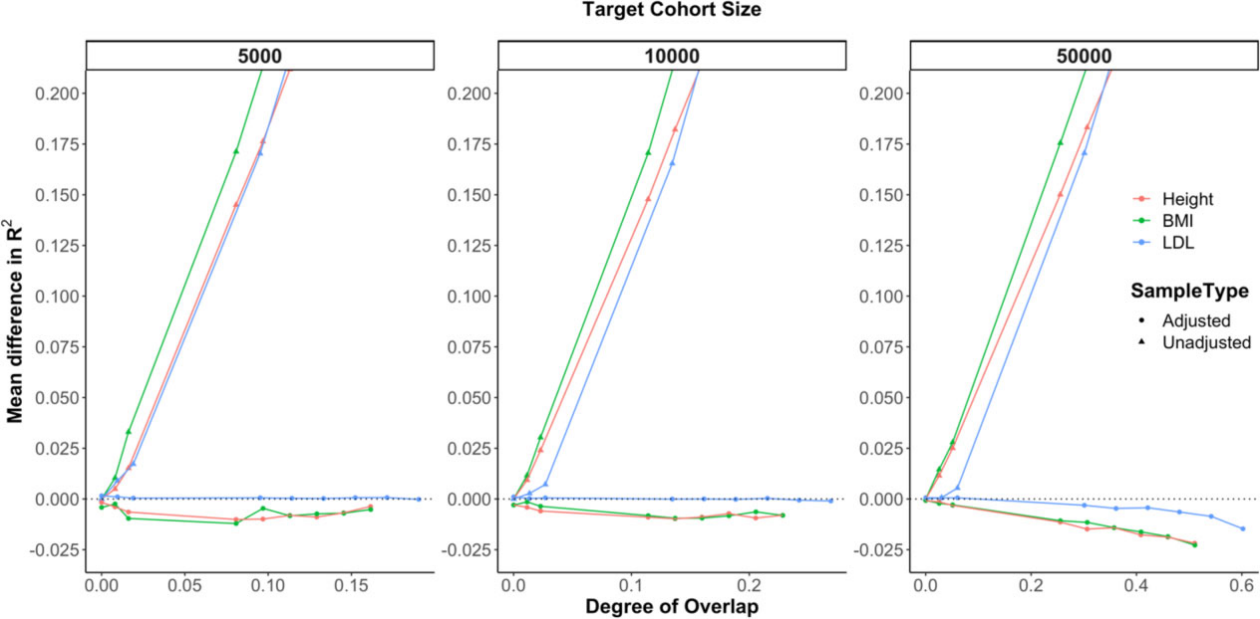
Comparing the performance of PRS using the EraSOR-adjusted summary statistics and the unadjusted summary statistics in real UK Biobank phenotypes. The x-axis shows the degree of overlap, and the y-axis shows the mean difference between the observed *R*^2^ and the expected *R*^2^. Mean difference in *R*^2^ = 0 is represented by the black dotted line. Each column corresponds to different target cohort size, and different colors correspond to different traits. Performance of the adjusted PRS is indicated with circle, and performance of the unadjusted PRS is indicated with triangle. Shaded area represents the 95% confidence interval, which tends to be small.

We performed real data validation of our simulations using height, BMI and LDL data from the UK Biobank. In all scenarios, EraSOR completely removed the effects of sample overlap, albeit with slight overcorrection in height and BMI (Fig. 3 and Supplementary Table S2).

To further evaluate the performance of EraSOR in real data settings, we used the latest GLGC LDL GWAS summary statistics and the LDL data from the UK Biobank. Using the European-specific GLGC summary statistics, where the UK Biobank samples were removed, the expected PRS *R*^2^ was 0.102. When the GWAS summary statistics contain the UK Biobank samples, the PRS *R*^2^ is 0.205, almost double the value without sample overlap. However, this inflation was alleviated when we used the EraSOR-adjusted summary statistics, which yielded a PRS *R*^2^ of 0.0918, representing a relative decrease of 10% compared to the overlap-removed PRS *R*^2^, consistent with our simulation results.

We note that EraSOR relies on the intercept term from LD score regression, the estimate of which assumes that the level of genetic and environmental stratification is similar between the base and target cohorts [13]. When we used the trans-ancestry GLGC GWAS summary statistics, the PRS *R*^2^ with the overlapping samples (UK Biobank) removed was 0.145, and the PRS *R*^2^ using the GWAS that included overlapping samples was 0.228, representing a relative increase of 57%. Although we did not observe the same inflation with the EraSOR-adjusted summary statistics, which yielded a PRS *R*^2^ of 0.0962, its performance was less accurate compared to the case where base and target ancestries were relatively matched, with a relative PRS *R*^2^ decrease of 33.5%, suggesting that EraSOR might be overly conservative in such settings.

Overall, our real data analyses confirmed the performance of EraSOR as predicted by our simulations, and we anticipated that EraSOR will be an appropriate tool for sensitivity analyses in real-world settings.

## Discussion

The recent advent of large-scale national and regional biobank projects, such as the UK Biobank [8], Japan Biobank [9], and FinnGen [11], has provided large resources of genotype–phenotype data ideal for conducting polygenic risk score analyses. However, this burgeoning generation of large data has led to an increased risk of intercohort sample overlap or relatedness, which can lead to inflated type I error. Due to privacy laws and practical concerns, it is usually impossible to identify overlapping samples or related samples across different cohorts. However, ideally, researchers would be aware of the scale of the potential problem and have tools to mitigate against it. Therefore, here we reported on an investigation to evaluate the impact of intercohort sample overlap and relatedness in PRS analyses and developed a method to account for potential intercohort overlap and relatedness that does not require access to raw genotype data from the base GWAS.

We demonstrated that intercohort overlap results in a significant and often substantial inflation in the observed PRS–trait association, coefficient of determination (*R*^2^), and false-positive rate. This inflation can be high even when the absolute number of overlapping individuals is small, if this makes up a notable fraction of the target samples. The inflation is noticeably more severe for binary traits with a small population prevalence when all the overlapping samples are cases. Therefore, PRS results will likely be misinterpreted unless intercohort sample overlap and close relatedness are properly accounted for.

Here, we developed the EraSOR method. EraSOR is designed to correct for intercohort sample overlap and relatedness using only summary statistics, without requiring any other information. The results of PRS analyses using EraSOR-adjusted GWAS results in the presence of sample overlap or relatedness were remarkably similar to those gained when the overlap was explicitly removed in most simulated conditions. EraSOR is also robust to misspecification of the model, for example, when there is environmental stratification. While EraSOR does not fully adjust for the bias introduced by intercohort overlap for nonheritable traits when the degree of overlap is high, we recommend that researchers should not perform PRS analyses on nonheritable traits in any case [1]. EraSOR performs well for most simulation scenarios tested here, which we believe reflect a large fraction of PRS studies.

Theoretically, as $\rho $ from the bivariate LD score regression is assumed to be the phenotypic correlation [19], we can apply EraSOR in situations where the base and target cohorts measure different phenotypes. Based on equations by LeBlanc et al. [17], the spurious correlations caused by intercohort overlap and relatedness are a function of the phenotypic correlation. While this suggests that the impact of intercohort overlap and relatedness is likely to be smaller for cross-trait analyses, EraSOR adjustments may still be beneficial in these scenarios. Investigation of the performance of EraSOR in cross-trait analyses should be the subject of future work. Further research is also required to understand the performance and biases of EraSOR for applications in cross-trait studies and in its potential application to GWAS meta-analyses.

Our algorithm is not without limitation. First, as EraSOR relies on LD score intercept estimates for adjustment, all assumptions of LD score regression also apply to EraSOR. For instance, if the level of genetic and environmental stratification differs between the 2 cohorts, the bivariate LD score equation may not hold [13], which can lead to bias in EraSOR estimates. Another limitation is that EraSOR has only been tested on data from the UK Biobank, which has a maximum genetic stratification corresponding to an *F_ST_* of approximately 0.02 (between European and non-European ancestry samples), and thus, we do not recommend applying EraSOR to data for which the *F_ST_* within or between base and target data is greater than 0.02. In practical terms, this means that until further testing or development of EraSOR has been performed, EraSOR should only be applied to single-ancestry base and target datasets that are closely matched by ancestry to each other. As it happens, cohorts of highly different ancestry are less likely to involve sample overlap. Additionally, EraSOR has only been tested using the C+T and lassosum PRS calculation method and so is subject to their limitations, such as potential overfitting of PRS–trait associations. However, given the similar performance of different PRS methods [29], we do not expect qualitatively different results to those observed here when EraSOR is applied to correct for sample overlap in PRS analyses using other PRS methods.

It is important to note that, due to reliance on LD score regression estimates, EraSOR only produces sufficiently accurate adjustments for application when both base and target cohorts have sample sizes greater than 1,000 and is only consistently accurate when both cohorts have greater than 5,000 samples. In addition, when there is a complete overlap between 2 equal-sized cohort, EraSOR can fail to fully adjust for the inflation caused by sample overlap. Despite this, EraSOR-adjusted results are still far closer to the empirical truth than the unadjusted PRS results. In general, EraSOR tends to overcorrect, meaning that PRS–trait associations are underestimated after EraSOR adjustment. One possible explanation is that the equations by LeBlanc et al. [17] were based on the null model of no contribution of SNP to the trait. Thus, in cases where SNPs contribute to the phenotype of interest, there will be an upper bound to the level of inflation caused by sample overlap, and EraSOR might misclassify some of the true signal as inflation caused by sample overlap, leading to overcorrection of test statistics.

Nevertheless, we argue that a large discrepancy between the unadjusted and EraSOR-adjusted results should act as a warning as to the possible presence of intercohort overlap or close relatedness. In such cases, it is recommended to investigate the potential source of sample overlap or relatedness and take appropriate measures to address them, such as removing the related or overlapped individuals, or adjusted for such bias using EraSOR to obtain more reliable PRS analysis results.

## Availability of Supporting Source Code and Requirements

Project Name: EraSOR

Project homepage: https://choishingwan.gitlab.io/EraSOR/

Programming language: Python (version 3.0+)

License: GNU General Public License version 3.0 (GPLv3)

Any restrictions to use by nonacademics: None

Simulation scripts: https://gitlab.com/choishingwan/sample_overlap_paper

biotools ID: erasor

RRID: SCR_023542

## Data Availability

All code used for this article is available in the GitLab repository [30] and implemented using nextflow (version 20.10.0 build 5430) [31]. Full source code and documentation of EraSOR can be found online [16]. The UK Biobank Resource is retrieved under Application Number 18,177 (controlled access). An archival copy of the code and supporting data is available via the *GigaScience* repository, GigaDB [32].

## Additional Files

**Supplementary Table S1**. Simulation results.

**Supplementary Table S2**. Results for real data analyses.

**Supplementary Figure S1** False positive rate corresponding to different level of sample overlap.

**Supplementary Figure S2** Effect of sample relatedness on performance of PRS for non-heritability phenotypes

**Supplementary Figure S3** Comparing the performance of the PRS using the EraSOR adjusted summary statistics and unadjusted summary statistics

**Supplementary Figure S4** Comparing the performance of PRS using the EraSOR adjusted summary statistics and the unadjusted summary statistics for quantitative trait when there are population stratifications.

**Supplementary Figure S5** Comparing the performance of PRS using the EraSOR adjusted summary statistics and the unadjusted summary statistics for binary trait analyse

**Supplementary Figure S6** Performance of EraSOR when adjusting for related samples between the target cohort and the base cohort

giad043_GIGA-D-22-00019_Original_Submission

giad043_GIGA-D-22-00019_Revision_1

giad043_GIGA-D-22-00019_Revision_2

giad043_GIGA-D-22-00019_Revision_3

giad043_Response_to_Reviewer_Comments_Original_Submission

giad043_Response_to_Reviewer_Comments_Revision_1

giad043_Response_to_Reviewer_Comments_Revision_2

giad043_Reviewer_1_Report_Original_Submission Christopher C. Chang, Ph.D. -- 2/14/2022 Reviewed

giad043_Reviewer_2_Report_Original_Submission Jack Pattee, Ph.D. -- 2/21/2022 Reviewed

giad043_Reviewer_2_Report_Revision_1 Jack Pattee, Ph.D. -- 10/19/2022 Reviewed

giad043_Reviewer_3_Report_Original_Submission Samuel A. Lambert -- 2/28/2022 Reviewed

giad043_Reviewer_3_Report_Revision_1 Samuel A. Lambert -- 11/3/2022 Reviewed

giad043_Reviewer_3_Report_Revision_2 Samuel A. Lambert -- 4/27/2023 Reviewed

giad043_Supplemental_File

## Abbreviations

BMI: body mass index; EraSOR: Erase Sample Overlap and Relatedness; GLGC: Global Lipids Genetics Consortium; GWAS: genome-wide association study; LDL: low-density lipoprotein; PC: principal component; PRS: polygenic risk score; QC: quality control; SNP: single-nucleotide polymorphism.

## Competing interests

The authors declare that they have no competing interests.

## Funding

Medical Research Council FundRef identification ID: http://dx.doi.org/10.13039/501100000265 MR/N015746/1 and the National Institutes of Health (R01MH122866) to P.F.O. This report represents independent research partially funded by the National Institute for Health Research (NIHR) Biomedical Research Centre at South London and Maudsley NHS Foundation Trust and King's College London. Research reported in this article was supported by the Office of Research Infrastructure of the National Institutes of Health under award number S10OD026880. The content is solely the responsibility of the authors and does not necessarily represent the official views of the National Institutes of Health, NHS, the NIHR, or the Department of Health.

## Authors’ Contributions

Conceptualization: S.W.C. and P.F.O. Methodology: S.W.C., T.S.H.M., C.H., and P.F.O. Investigation: S.W.C. Software: S.W.C. Supervision: P.F.O. Funding acquisition: P.F.O. Writing—original draft: S.W.C. Writing—review and editing: S.W.C., C.H., and P.F.O.
