# Supplementary material for: EraSOR: a software tool to eliminate inflation caused by sample overlap in polygenic score analyses
Source: Gigascience. 2023 Jun 16;12:giad043. doi: 10.1093/gigascience/giad043 (PMC10273836; doi:10.1093/gigascience/giad043)
Supplement: giad043_GIGA-D-22-00019_Revision_1 [file giad043_giga-d-22-00019_revision_1.pdf]

GigaScience

EraSOR: a software tool to eliminate inflation caused by sample overlap in polygenic score analyses

--Manuscript Draft--

**Manuscript Number:**	GIGA-D-22-00019R1
**Full Title:**	EraSOR: a software tool to eliminate inflation caused by sample overlap in polygenic score analyses
**Article Type:**	Technical Note
**Funding Information:**	National Institutes of Health (R01MH122866) — Dr Paul F. O'Reilly
**Abstract:**	Polygenic risk score (PRS) analyses are now routinely applied across biomedical research. However, as PRS studies grow in size, there is an increased risk of sample overlap between the genome-wide association study (GWAS) from which the PRS is derived and the 'target sample', in which PRS are computed and hypotheses are tested. Despite the wide recognition of the sample overlap problem, its potential impact on the results from PRS studies has not yet been quantified and no analytical solution has been provided. Here, we first conduct a comprehensive investigation into the scale of the sample overlap problem, finding that PRS results can be substantially inflated even in the presence of minimal overlap. Next, we introduce a method and software, EraSOR (Erase Sample Overlap and Relatedness), which eliminates the inflation caused by sample overlap (and close relatedness) in almost all settings tested here. EraSOR could be useful in PRS studies (with target sample > 1k) similar to those investigated here, either: (i) to mitigate the potential effects of known or unknown inter-cohort overlap and close relatedness, or (ii) as a sensitivity tool to highlight the possible presence of sample overlap before its direct removal, when possible, or else to provide a lower bound on PRS analysis results after accounting for potential sample overlap.
**Corresponding Author:**	Shing Wan Choi<br>Icahn School of Medicine at Mount Sinai<br>New York, UNITED STATES
**Corresponding Author Secondary Information:**	
**Corresponding Author's Institution:**	Icahn School of Medicine at Mount Sinai
**Corresponding Author's Secondary Institution:**	
**First Author:**	Shing Wan Choi
**First Author Secondary Information:**	
**Order of Authors:**	Shing Wan Choi
	Timothy Shin Heng Mak
	Clive J. Hoggart
	Paul F. O'Reilly
**Order of Authors Secondary Information:**	
**Response to Reviewers:**	Reviewer #1: This paper addresses a significant need that has arisen in the interaction between privacy rules and ever-larger genomic datasets, and I find the results to be very promising and clearly worth publishing. I just have a few comments on some methodological details:<br><br>>> We thank the reviewer for their positive comments on the utility and likely impact comment of our paper.<br><br>line 130: Have you compared the effectiveness of this algorithm with plink2 --king-cutoff?

Powered by Editorial Manager® and ProduXion Manager® from Aries Systems Corporation

>> The greedy related algorithm is different from plink2 --king-cutoff in that it does not calculate the relatedness between individuals but uses existing kinship calculation (pre-computed in the UK Biobank) for sample removal. It first ranks samples according to their number of related pairs based on the given kinship information, then removes the individual with the highest number of related pairs within the data, which should eliminate a high degree of relatedness while retaining a high sample size. This process iterates until there are no remaining closely related individuals. This algorithm is particularly useful for the UK Biobank data as the pairwise relatedness information was already calculated, allowing us to quickly remove related samples without recalculating the kinship information. If the kinship information is unavailable, then plink2 --king-cutoff can be used instead.

>> We have now explained this explicitly in the Methods section and have added the recommendation to use plink2 –king-cutoff if relatedness hasn't been pre-computed as it was in the UK Biobank.

lines 145-155: If I understand this correctly, these simulated quantitative traits are still normally distributed, they just aren't standardized to mean 0 variance 1. If the intent is to "simulate phenotypes that [do] not follow the standard normal distribution", I'd expect it to be more valuable to look at e.g. the log-normal case, where an alert user might transform the phenotype to normal, but some users may fail to do so. A mixture distribution may also be worth looking at.

>> Our intent here was only to perform a basic 'sanity check' that our results were unaffected when applied to a trait that does not strictly follow a standard normal distribution, rather than performing a fuller investigation of the impact of application to non-normally distributed traits that were not normalized by users. We have now made our intention here clear and have clarified that application of EraSOR assumes that non-normally distributed continuous traits have been normalized as standard.

lines 238-239: Have you considered using the "cc-residualize" option of plink2 --glm, which removes most of the computational cost of including PCs in your binary trait analysis?

>> We thank the reviewer for suggesting the `cc-residualize` option, which we were unaware of. We did consider performing a residualization of this kind, but decided not to because we did not simulate population stratification in the case control analyses and, thus, there was no need to control for PCs here.

lines 383-387: This is interesting; there is some room for follow-up investigation here. Thanks for posting all the scripts needed for another researcher to easily reproduce this Fst=0.00639 value; this could help facilitate development of a better genotype-simulation tool.

>> We thank the reviewer for their positive comments on this.

Also, some minor copyedits:
line 84: "subpopulation" -> "subpopulations"
line 342: "overlaps" -> "overlap"
line 363: "ErasOR" -> "EraSOR"
line 376: "different level of environmental stratifications" -> "different levels of environmental stratification"
line 384: "population" -> "populations"
line 402: "capture" -> "captured"

>> We thank the reviewer for pointing out these our typos. All of these typos have now been corrected in the revision.

Reviewer #2: Overall, I think that this manuscript is strong and describes a well-formulated method to address a relevant problem. There are a few outstanding questions about the performance of the EraSOR method from my perspective, which I'll detail as follows.

Powered by Editorial Manager® and ProduXion Manager® from Aries Systems Corporation

>> We thank the reviewer for their positive comments on our manuscript, the formulation of our method and the relevance of the problem that our method sets out to tackle.

My understanding of reference [16] indicates that equation (3) of this manuscript only holds for null SNPs, i.e. if SNP g is not associated with the outcome Y. If this is the case, then this should be discussed in the manuscript. I wonder if this can partially explain the 'under-estimation' behavior we see in the application to real data in Supplementary Figure 3. In particular, I am referencing the behavior where the EraSOR correction will under-estimate the predictive accuracy of the PRS in the target data, i.e. where delta-R^2 is negative. This behavior is not seen in the simulation and warrants further investigation and discussion. While the bias appears small, for some cases delta-R^2 approaches -.025, which corresponds to an under-estimation of Pearson's r by roughly .15; this is substantial. Could it be the case that, for highly polygenic traits such as height and BMI, the null-SNP assumption is unreliable and the performance of EraSOR is degraded? Does a fundamental assumption of sparse genetic association underlie EraSOR?

>> We thank the reviewer for highlighting this important point. Eq.2, and thus eq.3, are indeed based on an assumption of null SNPs, which we previously stated following Eq.1 but which we now repeat more explicitly at the end of the 'EraSOR framework' in the Methods section. Based on the reviewer's comments, we have now extended our quantitative simulations to incorporate 100k causal variants (i.e. 10x the fraction of causal variants as we originally simulated). These new simulation results indicate that the polygenicity of the trait is not the cause of the downward bias in the correction. On the other hand, when we avoid the use of EraSOR altogether and instead plug-in the known degree of sample overlap into LeBlanc's equation, then we observe a similar degree of downward bias, suggesting that this may be a result of a limitation in LeBlanc's equation. We have now highlighted this as a limitation in the Discussion.

I recommend that the real data application play a larger role in the manuscript narrative and be moved out of the supplementary. The simulations are appreciated and helpful, but there is nuance in the analysis of real data that cannot be replicated in simulation.

>> We agree with the reviewer that the real data analyses are extremely important in terms of involving relevant nuance that simulations cannot fully model and so we have now moved the entire real data application to the main text.

I believe the reference to "Supplementary Figure 2" on line 346 should actually be "Supplementary Figure 3". I believe that the axis labels in Supp Figure 3 are flipped.

>> Thank you for pointing out these errors. We have now fixed both the reference to the figure and figure axis labels.

Lines 82 and 83 reference genetic stratification and subpopulations; I think the relevance of these concepts should be introduced more clearly and they should be defined in this context. EraSOR concerns the over-estimation of predictive accuracy and association incurred by sample overlap between the base and target GWASs; to this reader, it's not clear what this central issue has to do with population stratification. I realize that the derivation of the LD score method is motivated heavily by correcting for stratification; however, these concepts should be introduced more clearly in this manuscript.

>> Genetic stratification, and relatedly here sub-populations, is only relevant here in that EraSOR leverages a fully expanded version of the LD score regression equation: one that was formulated by Yengo et al (ref.13) and incorporates two terms that include Fst (which measures genetic stratification). We have now expanded the corresponding section of the Methods to define genetic stratification in this context and to clearly explain its relevance in this setting, which involves inferring sample overlap from an equation that includes an explicit measure (Fst) of genetic stratification.

Line 88: consider defining LD score lj.

>> We have now included the definition of LD score in our updated manuscript.

Powered by Editorial Manager® and ProduXion Manager® from Aries Systems Corporation

Lines 94-96: consider outlining the mathematical consequence of the assumption that "the two outcomes and cohorts are identical." It's the case that N_1 = N_2 = N_c = N, correct?

>> That is correct. We have now outlined the mathematical consequence of this assumption, which we agree will help with the reader's understanding here.

Line 109 / equation (11): My understanding is that the relevant quantity of this derivation is N_c / sqrt(N_1 N_2), which allows us to define the correct matrix C in expression (4). If this is the case, perhaps the quantity of interest should be moved to the LHS of the equation in the final line of the expression, for clarity.

>> We thank the reviewer for pointing this out. N_c / sqrt(N_1 N_2) is indeed the quantity of interest. We have now updated equation 11 to have this quantity located on the LHS of the equation for clarity.

As discussed in the manuscript, the estimated heritability is in the denominator of the expression for N_c / sqrt(N_1 N_2). The authors correctly discuss that the method should not be applied when there is doubt as to whether the heritability is different from zero. I would take this a step further; in cases where the heritability is zero, we cannot meaningfully apply the EraSOR correction, and thus I am not sure of the utility of the 'type I error' simulations in the manuscript. Perhaps an explicit test for h^2 > 0 should be worked into the EraSOR workflow?

>> We thank the reviewer for their comment. Our paper has two main goals: 1) To demonstrate the problem of sample overlap in the performance of PRS analyses; and 2) to introduce the EraSOR algorithm. As such, the 'type 1 error' simulations in the manuscript are included primarily to demonstrate the problem of sample overlap convincingly by showing that highly significant results are obtained under overlap, even under the null of no causal variants or heritability.

>> However, we have now explained this point in detail in the main text for clarity, so that it is absolutely clear to the reader that EraSOR should not be performed in relation to PRS analyses that use GWAS data with low heritability (h2SNP < 5%), and in fact we state in our 'guide to PRS' paper (Choi et al. 2020. Tutorial: a guide to polygenic risk score analyses') that PRS analyses should not be performed using GWAS with estimated h2SNP < 5%. Moreover, following the reviewer's suggestion, we have now expanded the pipeline of the EraSOR code to include a quality control step that estimates h2SNP in the base GWAS (provided by the user) and outputs a message to the reader stating that EraSOR is not performed in relation to base GWAS with h2SNP < 5%, since we do not recommend PRS analyses be performed if the base GWAS has estimated h2SNP < 5%.

Line 148 / expression (12): If beta has a normal distribution here, it is the case that all SNPs in the simulation are associated with the outcome Y. This is a somewhat unusual choice for the distribution of SNP effects in a simulation; other applications such as LDPred (Vilhjalmsson et al, AJHG 2015) and LassoSum (TSH Mak et al, Genetic Epi 2017) use a point-normal distribution for simulated SNP effects, which effectively simulates the sparsity frequently observed in nature. Is there a reference or justification for the non-sparse simulation structure here?

>> We thank the reviewer for their comment. In equation 12, we are in fact using a point-normal distribution for simulating SNP effects similar to that used in LDpred and lassosum. Here, only 10,000 SNPs have a non-zero effect size. We have now updated the manuscript to clarify this simulation.

Line 215: there may be a typo in the expression for the variance of the residual term. Is it the case that the variance of the residual depends on the variance of a covariance term? If so, I am confused as to the derivation.
>> We thank the reviewer for pointing this out. There was a typo in the residual term where var(Xβ+S-2cov(Xβ,S)) should have been included within the square root expression. We have now corrected this mistake.

Powered by Editorial Manager® and ProduXion Manager® from Aries Systems Corporation

Line 241: 'triat' should be 'trait'.

>> We thank the reviewer for spotting this error. We have now corrected this in the updated manuscript.

The simulation results in this paper are based on clumping and thresholding for PRS, which does not estimate joint SNP effects i.e. account for LD. Methods such as LDPred and LassoSum do so. Is there any reason to believe the results would be different for a method such as LassoSum?

>> We thank the reviewer for their comment. While methods such as LDPred and LassoSum do account for the correlation between SNPs in a statistically principled way, the 'clumping' process of the standard PRS computation method does account for the correlation between the effects of different SNPs, albeit in a rather simplified way that acts only as a proxy for a full joint model of SNPs (see Choi et al. 2020. Nature Prot). Therefore, given this, and the relatively similar performance in trait prediction of all of these PRS calculation methods, then we do not believe that the results should be systematically different if using methods such as lassosum or LDpred. It is computationally prohibitive to perform our comprehensive simulations and benchmarking using methods such as lassosum or LDpred, but we have now discussed this point in the Discussion section and made clear that a limitation of our work is that we have not checked the impact of using EraSOR to correct PRS analyses with sample overlap performed using one of the multiple other PRS methods available (although we expect the results not to differ qualitatively since such methods will also be affected by sample overlap similarly and EraSOR is applied only to the base and target GWAS data, which are independent of any PRS analyses).

I am confused by the very low Fst between the simulated Finnish and Yoruban samples in simulation. As detailed on line 385: the reported Fst is > .1, but the simulated Fst is essentially zero. This seems likely to be an undesirable simulation artefact, and potentially invalidates the simulation study (or, at least, doesn't provide evidence that EraSOR functions correctly when Fst is large, which was the ostensible motivation for this simulation). Is there no way to effectively simulate populations with a larger Fst?

>> Unfortunately, after much time spent to try to resolve why HapGen-2 is not generating samples with large Fst in our analyses, and after contacting the HapGen-2 authors about the issue, we have not found a solution to this. Following the reviewer's point about this, we have decided to remove this HapGen-2 simulation study from the manuscript given the possibility that the results are subject to a simulation/coding artefact, and instead rely on the analyses performed in the UK Biobank in which we generate samples with an FST of 0.018 (European and non-European ancestry samples), which is relatively high compared to European-only samples.

>> In the Discussion we now explain that a limitation of our study is that we have only tested the performance of EraSOR in the real data of the UK Biobank, and so that we can only recommend the use of EraSOR in settings in which the base and target data are drawn from populations of the same or similar ancestry (at least no greater than random samples within the UK). However, the likely impact of this limitation is reduced by the fact that base and target samples that are drawn from different ancestral populations are substantially less likely to contain sample overlap in any case.

Reviewer #3: In this paper Choi et al. describe EraSOR, a new tool to remove the effects sample overlap between a set of summary statistics and a target dataset. EraSOR works by running a GWAS in the target dataset and then using LD-score regression techniques to estimate the heritability, genetic correlations of the phenotypes, and number of overlapping samples to decorrelate the effect sizes. The method is thoroughly described, and the simulation scenarios are relevant and well-motivated. However, the manuscript could better describe the inputs and characteristics of the decorrelated summary statistics, focusing more on the degree of bias in effect sizes rather than p-value inflation, and the practicalities of how the tool

Powered by Editorial Manager® and ProduXion Manager® from Aries Systems Corporation

may be used.

>> We thank the reviewer for their helpful comments, which have helped us to produce, we believe, a substantially improved manuscript.

Specific Comments:

* The results of Figure 1/Supp Figure 1 are highly motivating, but the p-value of the association doesn't seem like the perfect measure of inflation. Plots of the effect size of the PRS compared to its expected effect (0, based on heritability) would better illustrate this.

>> We thank the reviewer for their comment. The main purpose of Figure 1 is to show the effect of sample overlap on the increase of false positive results and so feel that this is made clearest through a focus on the impact on P-values rather than change in effect size estimates. We did consider specifically highlighting false-positive rates rather than P-values but the false-positive rates are mostly 100% even with limited overlap and so we decided to illustrate the impact on P-values directly to retain greater information and differentiation between scenarios (e.g. different target sample sizes).

* The paper proposes a method to remove the effects of sample overlap on summary statistics, but instead mostly focuses on how overlap biases the results of PRS prediction. Additional exploration of the decorrelated summary statistics themselves is needed to illustrate the validity of the method. Specifically, how different are the EraSOR adjusted summary statistics from the true summary statistics measured without sample overlap (e.g. distribution of effect sizes differences); what types of variants does EraSOR fail for or overcorrect (e.g. MAF differences between the summary statistics and the target cohort)? Are the results used as-is in other analyses, or do they have to be filtered in some way?

>> We thank the reviewer for their comments. The main motivation of the current paper is to specifically tackle the problem of sample overlap in PRS analysis. There are many algorithms designed that account for sample overlap in the context of other genetic studies, for example, METAL has an algorithm for meta-analysis; MTAG is designed to account for sample overlap in cross-trait GWAS analyses; whereas LDSC and LDAK account for sample overlap in heritability estimation. To our knowledge, EraSOR is the only algorithm that can account for an unknown degree of sample overlap in PRS analyses. As such, the main focus of the current paper is its utility for PRS analyses, which we believe should be highly impactful given the large number of PRS analyses being published presently, almost all of which have unknown sample overlap and thus potentially questionable results and conclusions. Our results present extensively on how EraSOR performs in terms of PRS-trait associations across a range of scenarios, each compared to associations observed with sample overlap removed. However, we agree with the reviewer that the statistical development of EraSOR could be useful in other settings, which we think will make our paper an important starting point not only for PRS applied studies but also for methodological follow-up in other settings.

>> In terms of whether results need post-EraSOR filtering – no, the EraSOR corrected GWAS results can be used as-is, albeit the use of EraSOR assumes that the standard GWAS quality control and filtering processes have already been applied to both the base and target data (as recommended in our 'PRS guide paper': ref.1). We have now made this clear in the manuscript and in our new detailed documentation of EraSOR that we produced in response to this and subsequent points from the reviewer (https://choishingwan.gitlab.io/EraSOR/).

* The PRS analyses in the paper all use PRSice to perform clumping+thresholding, selecting the best p-value and LD thresholds on the target datasets. This could be considered overfitting to the target data, and other derivation methods that do not require a sample to optimize hyperparameters (e.g. PRS-cs, LDpred-auto) could be used. It would be good to provide some additional analyses showing that EraSOR outputs also work with other methods of PRS derivation, and that the results are not sensitive to overfitting through hyperparameter optimization.

>> We thank the reviewer for their comment. We acknowledge that the results from the C+T analyses may be subject to overfitting and we have now highlighted this in the

Powered by Editorial Manager® and ProduXion Manager® from Aries Systems Corporation

Discussion. However, the problem of overfitting, and thus PRS calculation accuracy, is independent of inflation caused by sample overlap. Therefore, the use of the EraSOR adjustment, and thus the difference between adjusted, unadjusted and observed results, should not be sensitive to overfitting (or underfitting) or the specific PRS calculation method used. We opted to use the C+T approach, using our PRSice implementation, because it remains the default and most popular PRS calculation method and because our PRSice software is highly computationally efficient, allowing us to perform a huge range of scenarios to evaluate the performance of EraSOR in different settings, which would not have been possible had alternative PRS methods also been included.

* The PRS analysis of the real phenotype data in UKB should be expanded. Currently the analysis uses summary statistics derived in UKB with varying levels of overlap; however, this does not match the real scenario that EraSOR will likely be used in (applying EraSOR to an externally-sourced GWAS and applied to UK Biobank). The authors should perform a descriptive analysis to show that EraSOR is useful in this real-world scenario by downloading summary statistics from the GWAS Catalog (with and without inclusion of UK Biobank), applying EraSOR, and quantifying the difference in accuracy (r2) and effect size. On a related note: does the ancestry of the summary statistics have to perfectly match the target cohort? How well does EraSOR work with multi-ancestry summary statistics where the LD-panel might be mismatched?

>> The main reason why externally sourced GWAS were not used in the current analyses is that without access to the raw genotyped data, we cannot establish the empirical truth and would not know whether results generated by EraSOR reflect scenarios where the sample overlap is known. Limited by data availability, we can only perform large-scale real data analyses using UK Biobank data where we can estimate the expected R2 and effect size of the PRS association according to retaining and removing known sample overlap to assess the performance of EraSOR.

>> In the Discussion, we now make clear that EraSOR has only been tested in settings of ancestry-matched target data, whereby both base and target data sets are predominantly single ancestry (see final response to reviewer 2), and so can only recommend its use in these settings presently.

* The point about insufficient adjustment the authors raise on lines 336-42 is quite important. Proper signposting about the limits of the decorrelation is needed in the software description and the discussion. From this passage that the authors suggest that known sample overlap should be avoided and EraSOR should only be used as a sensitivity analysis to ensure that overlap does not exist? It would be useful to get the authors perspective on whether the evaluation of a PRS in a cohort derived using EraSOR-adjusted summary statistics can be seen as truly external to the source GWAS.

>> We thank the reviewer for their comment. The issue raised on lines 336-42 is specific to the scenario where *all samples* from the target were found in the base data. In this scenario, the EraSOR adjustment is unlikely to be optimal because of the complete degree of overlap, but will still generate results much closer to the empirical truth when compared to the unadjusted results, making this an ideal sensitivity analyses. In this scenario, EraSOR-adjusted summary statistics cannot be viewed as truly “free” of overlap – in the same way as imputed genotypes cannot be viewed as the same as true genotypes (but are useful estimates of them) – and deviation of EraSOR adjusted results and the unadjusted results should be treated as a possible red flag for analysts, informing them about the possibility of overlapping samples between their base and target data. In this case, they should reach out to the GWAS cohort to request leave-one-out GWAS results for their PRS analyses to achieve perfect non-overlap between base and target data, which is always preferable to a mathematical approximation to non-overlapping data. We have now expanded our limitation section to better describe this scenario and our perspective on it.

* The paper should be accompanied by a more detailed user guide and some test data for the EraSOR tool. Are there any diagnostic plots that are produced that could be used to inspect the data quality?

	>> We thank the reviewer for their comment. We have included a detailed documentation, including diagnostic plots, in https://choishingwan.gitlab.io/EraSOR/ and have included test data for users to familiarise themselves with the use of EraSOR.
**Additional Information:**	
**Question**	**Response**
Are you submitting this manuscript to a special series or article collection?	No
**Experimental design and statistics**<br><br>Full details of the experimental design and statistical methods used should be given in the Methods section, as detailed in our Minimum Standards Reporting Checklist. Information essential to interpreting the data presented should be made available in the figure legends.<br><br>Have you included all the information requested in your manuscript?	Yes
**Resources**<br><br>A description of all resources used, including antibodies, cell lines, animals and software tools, with enough information to allow them to be uniquely identified, should be included in the Methods section. Authors are strongly encouraged to cite Research Resource Identifiers (RRIDs) for antibodies, model organisms and tools, where possible.<br><br>Have you included the information requested as detailed in our Minimum Standards Reporting Checklist?	Yes
**Availability of data and materials**<br><br>All datasets and code on which the conclusions of the paper rely must be either included in your submission or deposited in publicly available repositories (where available and ethically appropriate), referencing such data using	Yes

a unique identifier in the references and in the “Availability of Data and Materials” section of your manuscript.<br><br>Have you have met the above requirement as detailed in our Minimum Standards Reporting Checklist?	

Main Manuscript
Click here to access/download;Manuscript;Main_101822.docx

EraSOR: a software tool to eliminate inflation caused by sample overlap in polygenic score analyses

Shing Wan Choi [1,2*], Timothy Shin Heng Mak [3], Clive J. Hoggart [1], Paul F. O'Reilly [1,2*]

1 Department of Genetics and Genomic Sciences, Icahn School of Medicine, Mount Sinai, 1 Gustave L. Levy Pl, New York City, NY 10029, USA;

2 MRC Social, Genetic and Developmental Psychiatry Centre, Institute of Psychiatry, Psychology and Neuroscience, King's College London, De Crespigny Park, Denmark Hill, London, UK, SE5 8AF; and

3 Centre of Genomic Sciences, University of Hong Kong, 21 Sassoon Road, Pokfulam, Hong Kong SAR, China

*To whom correspondence should be addressed

Abstract

Polygenic risk score (PRS) analyses are now routinely applied across biomedical research. However, as PRS studies grow in size, there is an increased risk of sample overlap between the genome-wide association study (GWAS) from which the PRS is derived and the 'target sample', in which PRS are computed and hypotheses are tested. Despite the wide recognition of the sample overlap problem, its potential impact on the results from PRS studies has not yet been quantified and no analytical solution has been provided. Here, we first conduct a comprehensive investigation into the scale of the sample overlap problem, finding that PRS results can be substantially inflated even in the presence of minimal overlap. Next, we introduce a method and software, EraSOR (Erase Sample Overlap and Relatedness), which eliminates the inflation caused by sample overlap (and close relatedness) in almost all settings tested here. ~~We recommend that~~ EraSOR could be useful~~be used~~ in~~ all future~~ PRS studies (with target sample > 1k) similar to those investigated here, either: (i) to mitigate the potential effects of known or unknown inter-cohort overlap and close relatedness, or (ii) as a sensitivity tool to highlight the possible presence of sample overlap before its direct removal, when possible, or else to provide a lower bound on PRS analysis results after accounting for potential sample overlap.

Introduction

Polygenic risk scores (PRSs) are proxies of individuals' genetic liability to a trait or disease [1] that have been applied in a range of~~numerous~~ research settings, including patient stratification [2] and investigation of treatment response [3–6]. The power of PRS analyses that test a study hypothesis is dependent on the

heritability and polygenicity of the trait, the power of the genome wide association study (GWAS) used to derive the PRS, and the size of the target data sample used to test the hypothesis [7]. The recent surge in availability of high quality genotype-~~etic and~~ phenotype~~ic~~ data from large-scale biobank projects, such as the UK Biobank [8], BioBank Japan [9], Taiwan Biobank [10], and FinnGen [11], as well as GWAS resources from large consortia such as the Psychiatric Genomic Consortium (PGC) [12], GIANT [13] and the Global Lipids Genetics Consortium (GLGC) [14], have provided unprecedented opportunities~~y~~ to perform highly-powered PRS analyses.

However, expansion in data sizes ~~does not~~ comes with~~out~~ a cost in this setting: as sample sizes increase, it is more likely~~there is greater risk~~ that samples are recruited into multiple cohorts or that entire cohorts are included in multiple consortia. For PRS analyses, which typically test for association between PRS and a trait(s) or outcome of interest, overlapping samples between the GWAS and target data samples can result in spurious inflation of the coefficient of determination (R^2) and association P-values, leading to false-positive and exaggerated findings [15]. If an entire cohort is present in the base GWAS and target data, then ideally it should be removed as follows: (i) directly remove the cohort from the GWAS data (recompute base GWAS results), (ii) if (i) is not an option, then use the cohort GWAS results to derive the base GWAS results minus the cohort, an analytical solution of which we have previously described, (iii) remove cohort from the target data directly if doing so does not compromise power. Likewise, o~~O~~verlapping individuals~~samples~~ should ideally be removed from either the GWAS or target data to avoid misinterpretation of results, but participant privacy agreements usually limit access to raw genotyping data, meaning that this is generally not an option.

Here we first evaluate the extent to which different degrees of sample overlap and relatedness between GWAS and target samples generates biased PRS-trait associations. Next, to overcome the sample overlap problem, we develop and introduce EraSOR (Erase Sample Overlap and Relatedness), a ~~python~~ software that adjusts GWAS summary statistics [1] to correct for inflation of PRS-trait association results caused by overlapping samples between the GWAS and target samples. Through extensive simulations using the UK Biobank genetic data [8], we demonstrate that EraSOR can robustly adjust for inflation in test statistics caused by various degrees of overlapping samples and~~,~~ close~~level of~~ relatedness, under different~~or~~ ascertainment schemes in case/control settings. ~~We propose that~~ EraSOR should~~will~~ increase the accuracy of results in all future PRS studies with known sample overlap and will act as a critical~~-~~ sensitivity tool as part of PRS analyses to ensure the ~~for assessing the~~ reliability of results in PRS studies with unknown but potential sample overlap. EraSOR is an open-source software and is freely available at **https://gitlab.com/choishingwan/EraSOR**.

Methods

EraSOR framework

Consider two GWAS $k = \{1, 2\}$ performed on the same continuous outcome Y_k. The effect size of the gth SNP in study k (β_{kg}) is estimated using a regression model

$$Y_k = \alpha_{kg} + \beta_{kg}X_{kg} + \varepsilon_{kg} \quad (1)$$

where X_{kg} is the standardized genotype vector for SNP g in study k, and ε_{kg} is the random error assumed to be independent between studies. Under the null model of no contribution of SNP g to the trait, $\beta_{kg} = 0$, and assuming no sample overlap, then $\widehat{\beta_{1g}}$ and $\widehat{\beta_{2g}}$ estimated from the two GWASs should be independent, i.e., $cor\left(\widehat{\beta_{1g}}, \widehat{\beta_{2g}}\right) = 0$. However, when there are overlapping samples between the two studies, then a correlation is induced between the regression coefficients, such that $cor\left(\widehat{\beta_{1g}}, \widehat{\beta_{2g}}\right) \neq 0$. From LeBlanc et al [16], this correlation can be approximated as

$$cor\left(\widehat{\beta_{1g}}, \widehat{\beta_{2g}}\right) \approx \frac{N_c}{\sqrt{N_1N_2}} cor(Y_1, Y_2) \quad (2)$$

for quantitative traits, where $cor(Y_1, Y_2)$ represents the correlation between the traits; N_c is the number of overlapping samples; and N_1, N_2 are the sample sizes of studies 1 and 2, respectively [16]. Since we are considering only a single phenotype here, $cor(Y_1, Y_2)$ is equal to 1, and so we have:

$$cor\left(\widehat{\beta_{1g}}, \widehat{\beta_{2g}}\right) \approx \frac{N_c}{\sqrt{N_1N_2}} \quad (3)$$

which captures correlations ~~only~~ due only to sample overlap~~,~~ independent of the true causal effect (n~~.~~ote that if the cohorts were also identical then N_1=N_2=N_c and thus, appropriately, both sides of (3) would equal 1). Assuming sample overlap does not affect~~s~~ the standard error estimates, LeBlanc et al [16] proposed that when the number of overlapping samples (N_c) is known, one can adjust the joint distribution of the summary statistics (z-scores) of the two GWASs as:

$$\boldsymbol{z_{de-corr}} = \boldsymbol{C}^{-0.5}\boldsymbol{z} \quad (4)$$

where $\boldsymbol{z}$ is a 2-by-M matrix containing z-scores estimated in each study, M is the number of SNPs common to both studies, and $\boldsymbol{C}$ is the 2x2 matrix with ones as~~on~~ its diagonal elements and $cor\left(\widehat{\beta_{1g}}, \widehat{\beta_{2g}}\right)$ as its off-diagonal elements. While this adjustment is effective [16], it requires prior knowledge of N_c, which is typically unknown in PRS studies. ~~However, w~~Here, we propose utilizing univariate and bivariate LD score regression [17,18] to estimate $\frac{N_c}{\sqrt{N_1N_2}}$ and thus $cor\left(\widehat{\beta_{1g}}, \widehat{\beta_{2g}}\right)$ from Eq. 3 as follows described below. ~~:~~

Bivariate LD score regression [18] is typically used to estimate the genetic correlation between two traits using two GWASs corresponding to each. In the formulation of bivariate LD score regression, g~~G~~enetic stratification – which corresponds to structure in genetic variation in a population due to non-random mating – ~~within each GWAS~~ is assumed to be similar in each GWAS sample, since making this assumption simplifies the mathematics and is approximately true in many settings. ~~and described by a 50:50 mixture of two subpopulations.~~ Yengo et al [13] generalized this equation by introducing Wright's F_{ST}, which measures ~~the~~ genetic~~population~~ stratification~~difference due to genetic structures~~, and made the simplifying assumption that genetic structure in a population is caused by the population comprising two sub-populations, with mating occurring mostly within, rather than between, the sub-populations. In this way, ~~,~~ F_{ST}~~,~~ here measures the genetic differences between two sub-populations that make up the overall population. Yengo et al ~~between the two subpopulations and~~ further introduced an environmental stratification term, σ_S, which is the mean phenotypic difference between the sub-populations. This leads to~~gives~~ the following equation:

Formatted: Font: Italic

Formatted: Space After: 0 pt, Don't adjust space between Latin and Asian text, Don't adjust space between Asian text and numbers

$$\mathbb{E}[z_{1j}z_{2j}] = \frac{\sqrt{N_1N_2}\rho_g}{M} l_j + \frac{N_c\rho}{\sqrt{N_1N_2}} + \rho_g F_{ST}^2 \sqrt{N_1N_2} + \frac{N_c^2 F_{ST}\sigma_S^2}{\sqrt{N_1N_2}} \quad (5)$$

where l_j is the 'LD score' ~~of SNP j~~defined as the sum of pairwise squared correlations between genotypes~~minor allele counts~~ at SNP j and all~~versus~~ other SNPs within the same LD block; ρ_g is the genetic covariance between the two traits; $\rho = \rho_g + \rho_e$; ρ_e is the non-genetic covariance; F_{ST} and σ_s are the genetic and environmental stratification respectively~~.~~ [13]. Since we are considering only a single phenotype here, ρ is equal to 1, and so we have:

Formatted: Font: Italic

$$\mathbb{E}[z_{1j}z_{2j}] = \frac{\sqrt{N_1N_2}\rho_g}{M} l_j + \frac{N_c}{\sqrt{N_1N_2}} + \rho_g F_{ST}^2 \sqrt{N_1N_2} + \frac{N_c^2 F_{ST}\sigma_S^2}{\sqrt{N_1N_2}} \quad (6)$$

We wish to solve for N_c and hence apply Eq. 3 to generate a de-correlated base GWAS that does not lead to inflated PRS-trait associations due to sample overlap. To do this, we will utilize the univariate LD score regression model~~. The univariate LD score regression equation~~, which can be derived as a special case of the bivariate LD score equation by assuming that the two outcomes and cohorts are identical [13,17] (ie.~~eg.~~ $N_1 = N_2 = N_c = N$), leading to:

$$\mathbb{E}[\chi_j^2] = \frac{Nh^2}{M} l_j + 1 + NF_{ST}(h^2 F_{ST} + \sigma_S^2) \quad (7)$$

Univariate LD score regression performs a regression of observed χ^2 on l_j, with the effect size estimate of l_j corresponding to a scaled estimate of heritability ($\widehat{h_i^2}$) and with the estimated intercept term, $\widehat{I_u}$ as follows:

$$\widehat{I_u} = 1 + N_i F_{ST}\left(\widehat{h_i^2} F_{ST} + \sigma_S^2\right)$$

A key observation by Yengo et al [13] is that in addition~~al~~ to the level of sample overlap, the inflation of the bivariate LD score regression intercept is also affected by the level of genetic~~s~~ and environmental stratification. As such, the Bivariate LD score regression intercept cannot directly be used as an estimate of the level of sample overlap. However, i~~I~~f we assume that the environmental stratification $\sigma_S^2 = 0$, then we have:

$$\begin{aligned} \widehat{I_u} &= 1 + N_i F_{ST}\left(\widehat{h_i^2} F_{ST} + \sigma_S^2\right) \\ \widehat{I_u} &= 1 + N_i F_{ST}^2 \widehat{h_i^2} \\ F_{ST}^2 &= \frac{\widehat{I_u} - 1}{N_i \widehat{h_i^2}} \end{aligned} \quad (8)$$

Since we can estimate F_{ST}^2 using both the base and target data, we ~~then~~ can take the weighted mean estimate of both:

$$\widehat{F_{ST}^2} = \frac{1}{N_1 + N_2} \sum_{i=1}^{2} \frac{\widehat{I_i} - 1}{\widehat{h_i^2}} \quad (9)$$

The intercept term of the bivariate LD score regression is:

$$\widehat{I_b} = \frac{N_c}{\sqrt{N_1 N_2}} + \widehat{\rho_g}\widehat{F_{ST}^2}\sqrt{N_1 N_2} + \frac{N_c^2 F_{ST}\sigma_S^2}{\sqrt{N_1 N_2}} \quad (10)$$

Substituting Eq. 9 and $\sigma_S^2 = 0$ into Eq. 10, we have:

$$\widehat{I_b} = \frac{N_c}{\sqrt{N_1 N_2}} + \widehat{\rho_g}\widehat{F_{ST}^2}\sqrt{N_1 N_2} + \frac{N_c^2 F_{ST}\sigma_S^2}{\sqrt{N_1 N_2}}$$

$$\widehat{I_b} = \frac{N_c}{\sqrt{N_1 N_2}} + \widehat{\rho_g}\sqrt{N_1 N_2}\left(\frac{1}{N_1+N_2}\sum_{i=1}^{2}\frac{\widehat{I_i}-1}{\widehat{h_i^2}}\right)$$

$$\frac{N_c}{\sqrt{N_1 N_2}}\cancel{\widehat{I_b}} = \cancel{\frac{N_c}{\sqrt{N_1 N_2}} +} \frac{\widehat{\rho_g}\sqrt{N_1 N_2}}{N_1 + N_2}\sum_{i=1}^{2}\frac{\widehat{I_i}-1}{\widehat{h_i^2}} - \widehat{I_b} \quad (11)$$

Since we can estimate the genetic covariate ($\widehat{\rho_g}$), the trait heritability $\widehat{h_i^2}$ and the intercepts from the univariate and bivariate LD score regression analyses of the GWAS and target data, we can obtain an estimate of $\frac{N_c}{\sqrt{N_1 N_2}}$. Substituting this estimate into Eq. 3 will derive an estimate of $cor\left(\widehat{\beta_{1g}}, \widehat{\beta_{2g}}\right)$ that can be used to produce de-correlated GWAS z-statistics via Eq.4. EraSOR automatically performs the bivariate LD score and univariate LD score regression analyses on the GWAS summary statistics generated from the base and target data. Because of our assumption of $\sigma_S^2 = 0$, EraSOR may~~might~~ underperform when large environmental stratification~~s~~ is present~~were observed~~. In addition, Eq 2 models the inflation under the null. For highly polygenic traits, a high fraction of~~much of the~~ variants across the genome may~~might~~ deviate from the null, thus introducing~~introduce~~ bias into EraSOR adjustments. To test the performance of EraSOR, including its robustness to the modelling assumptions (e.g., assuming $\sigma_S^2 = 0$), we performed a series of extensive simulations.

UK Biobank genotype data

The UK Biobank is a prospective cohort study of around 500,000 individuals recruited across the United Kingdom during 2006-2010. The genetic data from UK Biobank comprises 488,377 samples and 805,426 SNPs. Standard quality control (QC) procedures were performed, removing any SNPs with minor allele frequency < 0.01, genotype missingness > 0.02 and with a Hardy Weinberg Equilibrium Test *P*-value < 1×10^{-8}. Samples with high levels of missingness or heterozygosity, with mismatching genetic-inferred and self-reported sex, or with aneuploidy of the sex chromosomes were removed as recommended by the UK Biobank data processing team. Next, 4-means clustering was applied to the first two Principal Components (PCs) of the genotype data and those individuals in the (largest) cluster corresponding to European ancestry were retained for the primary analyses because polygenic risk scores have been shown to have low portability between ancestries [14] motivating ancestry-matched PRS studies until cross-ancestry PRS methods are developed, which our main results correspond to (see section *Samples with population stratification* below, which describes analyses that we also performed on individuals of all ancestries in the UK Biobank). A greedy algorithm [19] was then used to remove related individuals, taking advantage of pre-computed pairwise kinship coefficients provided by the UK Biobank. The algorithm first ranks individuals by their number of related pairs (kinship coefficient > 0.044), then

Formatted: Left

removes the individual with the highest number of related pairs within the data, which should eliminate a high degree of relatedness while retaining a high sample size. When pre-computed kinship coefficients are unavailable, plink2 –king-cutoff can be used to achieve relatedness removal similarly. In our simulations that investigate the effect of related individuals in the GWAS and target data, we instead randomly retain one first degree relative (defined as kinship coefficient ≥ 0.177 and ≤ 0.354) of a randomly sampled individual in the GWAS data. Altogether, we retain 557,369 SNPs, 387,392 individuals and 23,429 of their first-degree relatives for the set of analyses performed. For the simulations of population stratified samples, we extracted samples 10 standard deviations from the centroid of the European cluster and defined these as "non-European" samples. Quality control procedures were repeated using the parameters described above after combining these non-European samples with the European samples, resulting in 387,365 samples of European ancestry and 21,779 individuals of non-European ancestry. Code used to perform the QC and corresponding documentation are available at **https://choishingwan.gitlab.io/ukb-administration/admin/master_generation/.** This research has been conducted using the UK Biobank Resource under application 18177 (Dr O'Reilly).

Phenotype simulation

Quantitative Traits without population structure

Quantitative phenotypes (*Y*) with heritability (h^2) of 0, 0.1, and 0.5 were simulated using the UK Biobank genotype data (post QC; see above) as input. Quantitative traits were simulated as:

$$Y = (\alpha + X\beta + \varepsilon)\delta \qquad (12)$$

where *X* is the standardized genotype matrix corresponding to all samples and the β vector corresponds to the effect size associated with each SNP, with either 10k or 100k SNPs randomly selected to be causal with effect size $\beta \sim N(0, 1)$, $\beta = 0$ otherwise~~10,000 randomly selected SNPs with effect size β following a standard normal distribution~~. $X\beta$ was~~ere~~ adjusted such that it has mean 0 and variance ~~of~~ h^2; and ε represents the random error, which follows $\varepsilon \sim N\left(0, \sqrt{1 - h^2}\right)$. To ensure that EraSOR can be applied to traits that do not only follow a strict standard normal distribution (N~(0,1)) ~~works for distribution that are not only standard normal,~~ we included α as an intercept parameter (α ~ N(0,1)) and ~~the phenotypic mean randomly sampled from a normal distribution with mean 0 and standard deviation of 1, and~~ δ as a scaling parameter (δ ~ U(1,100))~~the phenotypic variable randomly sampled from 1 to 100 to simulate phenotypes that does not follow the standard normal distribution~~. This was ~~only~~ intended only as a basic test of robustness to deviations from a ~~non-~~standard normal distribution and we did not consider any more complex non-Gaussian trait distributions. Consequently,~~This means that~~ an assumption of EraSOR is that it is applied to continuous data that are either normally distributed or are normalised, using e.g. a log transformation or inverse normal transformation, as is standard in linear regression on continuous outcomes.

To model polygenic risk score analyses with sample overlap, we randomly selected either 120k or 250k individuals from the sample of 387,392 individuals available to us (see above) to generate two different sizes of base GWAS data. Next, we randomly sampled 1,000, 5,000, 10,000 or 50,000 individuals from the remaining sample to act as three different sizes of target data, of which 0%, 5%, 10%, 50% or 100% were randomly selected from the base data sample so that there was a known degree of sample overlap between the base and target data. In addition, we generated an "overlap-free" base cohort in which the overlapping

samples were removed from the base cohort so that we could compare the result of applying EraSOR against results of physically removing overlapped samples from the base cohort.

In order to search a feasible parameter space in sufficient depth, we only simulate phenotype with heritability of 0.5, with a base cohort of 250k and target cohort of 5,000; only simulate base cohort with 120k samples when the phenotypic heritability is ≤ 0.1 and target cohort has 5,000 samples; and only simulate target cohort with 1,000 and 10k samples when the base cohort contain 250k samples and the phenotypic heritability is ≤ 0.1. The entire set of simulations were repeated 100 times.

Binary Trait

Binary traits were simulated under the liability threshold model [20], simulating a normally distributed liability using Eq. 12 with α = 0, δ = 1, and cases defined as samples with disease liability higher than liability thresholds of 0.9, 0.7 and 0.5, corresponding to population prevalences of 0.1, 0.3 and 0.5, respectively. To limit the complexity of our simulations, the sample prevalence of our cohorts follows the population prevalence and we only simulated 10k causal variants.

In the binary trait setting, overlap can be ascertained such that the overlap is among cases, or among controls, or among both. To investigate the effect of case-only or control-only overlap, we randomly selected 120k effective samples (effective samples defined as $N_{eff} = 4/(1/N_{cases} + 1/N_{controls})$ [21]) as the base cohort, and then randomly selected 5,000 effective samples as the target cohort, where 0%, 5%, 10%, 30% or 50% of the cases or of the controls in the target cohort were sampled from the base cohort. We also performed simulations where the overlapping samples were selected at random among cases and controls. An "overlap-free" base cohort was generated with all overlapping samples removed.

In order to search a feasible parameter space in sufficient depth, we only vary the trait heritability when the population prevalence is 0.1, and only vary the population prevalence when the trait heritability is ≤ 0.1. These simulations were repeated 100 times.

Related samples

Spurious inflation in PRS analysis test statistics may also be observed when there are closely related individuals between the base and target cohorts. To investigate the effects of relatedness on PRS results, we repeated the quantitative trait simulations with a modified Eq. 12:

$$Y = (\alpha + X\beta + \theta + \varepsilon)\delta \qquad (13)$$

where θ is the shared environment between the related individuals and follows a random normal distribution with mean 0 and variance $\sigma_\theta^2 \in (0, 0.3, 0.6)$ if and only if $\sigma_\theta^2 + h^2 < 1$, with each related pair of individuals having the same θ value. Only simulated 10k causal variants, and with ε represents a combination of non-shared environment and random error, which follows $\varepsilon \sim N\left(0, \sqrt{1 - h^2 - \sigma_\theta^2}\right)$. To model the inter-cohort relatedness, we first select all individuals with a first-degree relative in the UK Biobank (kinship coefficient ≥ 0.177 and ≤ 0.354), of which there are 23,429 individuals, and then randomly select additional samples who do not have any first-degree relatives to form a base cohort containing 250k samples. We then generate target cohorts containing 5,000 samples, with either 0%, 30%, 60% or 100% of the target samples being first-degree relatives of samples in the base cohort. We also generated a reference cohort from the base cohort where all the related samples in the target cohort were

replaced by unrelated individuals for benchmarking the performance of EraSOR. The entire set of simulations were repeated 100 times.

Samples with population stratification

An assumption of the EraSOR algorithm is that the environmental stratification (σ_S^2) is zero. When environmental stratification is present, $\frac{N_c^2 F_{ST}\sigma_S^2}{\sqrt{N_1 N_2}}$ from Eq. 10 is no longer 0 and a bias proportional to the environmental stratification and the genetic stratification (F_{ST}) may be introduced. We devised two strategies for simulating datapartition the UK Biobank into European and non-European ancestries and simulated with both environmental and genetic stratifications to test the sensitivity of EraSOR to deviations of each from 0. In the first, we partitioned the UK Biobank into European and non-European ancestries, while in the second we used the simulation software HapGen2 [22].

In the first simulation strategy, the UK Biobank samples were divided into European and non-European ancestries based on 4-mean clustering on PC1 and PC2 (see above). Quantitative traits with environmental stratification were then simulated as:

$$Y = (\alpha + X\beta + S + \epsilon)\delta \quad (14)$$

with the environmental stratification term (S) defined as

$$S = \begin{cases} -\frac{\sigma_S}{2}, & \text{Non} - \text{European Ancestry} \\ \frac{\sigma_S}{2}, & \text{European Ancestry} \end{cases}$$

where σ_S^2 can take a value of 0, 0.3 or 0.9 if and only if $\sigma_S^2 + h^2 < 1$, and ϵ represents the residual term, which follows $\varepsilon \sim N\left(0, var(X\beta + S - 2cov(X\beta, S))\ \sqrt{var(X\beta + S - 2cov(X\beta, S))\ \frac{1-h^2-\sigma_S^2}{h^2+\sigma_S^2}}\right)$, with $\mathrm{cov}(X\beta, S)$ being the covariance between $X\beta$ and S. To investigate the effect of sample overlap in the presence of environmental and genetic stratification, we randomly selected either 120k or 250k individuals from the sample of 409,144 individuals available to us (see above) to generate two different sizes of base GWAS data. Next, we randomly sampled 5,000 or 10,000 individuals from the remaining sample to act as two different sizes of target data, of which 0%, 10%, 50% or 100% were randomly selected from the base data sample. To ensure that the genetic and environmental stratification is the same within the base and target data, the same ancestry ratio was maintained in all simulated data sets, matching the ratio in the full data set (~5% non-European ancestry). In addition, we generated an "overlap-free" base cohort in which the overlapping samples were remove from the base cohort to allow benchmarking the performance of EraSOR. The entire set of simulations were repeated 50 times.

Real UK Biobank phenotype

analysis

Formatted: Left

To investigate the effect of sample overlap in real phenotypic data, we extract Body Mass Index (BMI, field ID 21001), Height (field ID 50) and Low-Density Lipoprotein (LDL, field ID 30780) from the UK Biobank. We residualisze the phenotypes against age (field ID 21003), sex (field ID 31), genotyping

batch, UK Biobank assessment centre (field ID 54) and 40 Principal Components (PCs). For LDL, we additionally removed individuals~~samples~~ who were on statin medication (see Supplementary Materials), and additionally included fasting time (field ID: 74) and dilution factor (field ID: 30897) as ~~a~~ covariates. The residuals were standardis~~z~~ed and used as a phenotype for downstream analysis.

To model polygenic risk score analyses with sample overlap, we randomly sampled 2/3 of the individual with phenotypic information to generate the base GWAS data. Next, we randomly sampled 5,000, 10,000, 5,000 and 1/3 of the individuals with phenotypic information from the remaining sample to act as the target data, of which 0%, 5%, 10%, 50%, 60%, 70%, 80%, 90% or 100% were randomly selected from the base data sample so that there was a known degree of sample overlap between the base and target data. In addition, we generated an “overlap-free” base cohort in which the overlapping samples were removed from the base cohort so that we could compare the results of applying EraSOR with the~~against~~ results of direct~~physically~~ removal~~ing~~ of overlapping~~ed~~ samples from the base cohort. The entire set of simulations wa~~w~~s~~ere~~ repeated 50 times.

~~Given that only ~5% of the UK biobank samples correspond to individuals of non-European ancestry, the effect of genetics and environmental stratification may be limited. Thus, we developed a second strategy to test their effects in which we used HapGen2 [22] to simulate 180k Yoruban and 180k Finnish samples using recombination maps from the 1000 Genomes Project [23]. 500 “Finnish” samples and 500 “Yoruban” samples were selected to calculate the LD scores using LDSC (v1.0.1) and flashPCA (v2.0) [24] was used to calculate the first 15 PCs of the data.~~

~~We repeated the population stratification simulation using the HapGen2 simulated genotype data, with S represented now segregate according to the simulated population. The entire set of simulations were repeated 50 times.~~

Genome-~~-~~Wide Association Study and Polygenic Score Analysis

Genome-~~-~~wide association analyses (GWAS) were performed on the base and target cohorts using PLINK 2.0 (version 2021-08-04) [25] with the --*glm* function. As binary traits were only simulated for the European ancestry only analyses, where population structure was not simulated, and considering the computational cost of including covariates in the logistic regression, we did not include PCs in our binary trait analysis. On the other hand, quantitative traits were simulated in all scenarios, some of which are population stratified. Thus, we included 15 PCs as a covariate for our quantitative trai~~a~~t analyses. The resulting summary statistics were then provided to EraSOR to generate the adjusted summary statistics using European LD scores [17] calculated from 1,000 Genomes Project Phase 3 data [23]~~ or the LD scores calculated from a subset of the simulated genotypes (HapGen2 simulation) using LDSC (v1.0.1) [17]~~. PRS analyses using the adjusted, unadjusted, and the “overlap-free” summary statistics were performed using PRSice-2 (v2.3.5) [26] with the default settings. The R^2 and *P*-value of association of the PRS-trait tests were reported.

Strategy for Benchmarking

To investigate the level of spurious inflation caused by inter-cohort relatedness and overlapped samples, we first established a baseline PRS R^2, calculated using base cohorts without overlapped samples. The bias can then be measured as the observed PRS R^2 minuses the baseline PRS R^2 (ΔR^2), given the same phenotype and cohort sizes. For non-heritable traits, we also measure the level of false-positive, defined as any PRS with *P*-value < $1x10^{-4}$ [27].

On the other hand, to compare the performance of EraSOR with the optimal strategy of directly removing overlapping samples – an option that is typically not available – we calculate PRSs: (i) using summary

Formatted: Normal

statistics adjusted by EraSOR ("adjusted PRS") and (ii) using summary statistics generated from a base cohort with all overlapping and/or related samples removed ("overlap-free PRS"). We present the performance of EraSOR as the PRS-trait association R^2 of the adjusted PRS minuses the R^2 of the overlap-free PRS (ΔR^2). If EraSOR has successfully corrected for the sample overlap, then ΔR^2 should be close to 0.

Results

Inflation caused by overlap

The presence of overlapping samples between the base and target data sets is known to cause inflated association between polygenic risk scores (PRS) and ~~phenotypes ,~~phenotypes, but the extent and characteristics of the problem have not been described. Here, we performed extensive simulations using the UK Biobank [8] genotype data to investigate the inflation caused by different levels and types of inter-cohort sample overlap in relation to traits simulated with varying heritability and prevalence (see Methods). Base and target cohorts were generated with varying degrees of sample overlap, measured as $\frac{N_c}{\sqrt{N_1 N_2}}$, where N_c is the number of overlapping samples and N_1 and N_2 are the sample sizes of the base and target cohort, respectively. PRS analyses were conducted using the standard *clumping+thresholding* (C+T) PRS calculation method [1], implemented in *PRSice* [26,27].

We first estimated the false-positive rate induced by sample overlap by simulating non-heritable traits and recording the fraction of significant PRS-trait association (Supplementary Fig. 1). Highly significant associations between PRS and non-heritable phenotypes were observed when even limited inter-cohort sample overlap was present (Fig. 1). Specifically, for non-heritable quantitative traits, the inflation in association (e.g., *p*-value of association) is highly positively correlated with the degree of overlap (Pearson Correlation coefficient (γ) = 0.96, *P*-value < $2.2\text{x}10^{-16}$). For example, when there is a base cohort of 250k samples, target cohort of 5,000 samples and 250 overlapping samples (5% of target sample; degree of overlap = 0.0071) the false positive rate is 16%, while this increases to 90% when there are 500 overlapping samples (10% of target sample; degree of overlap = 0.014) (Fig 1a).

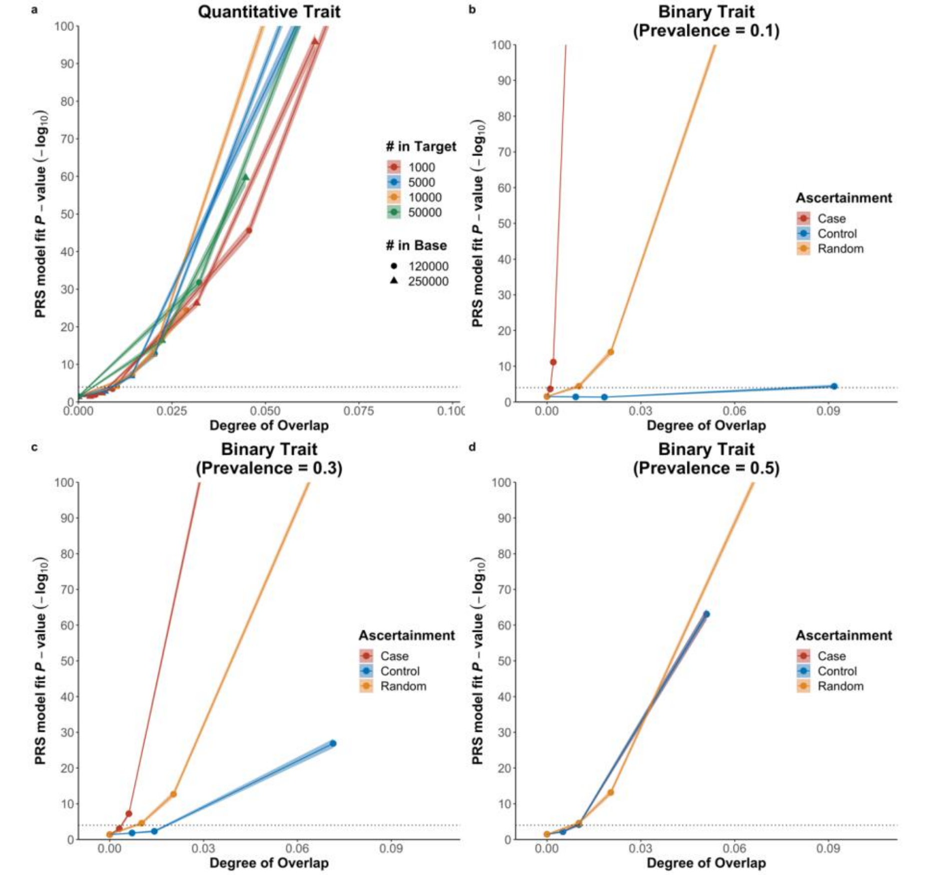

Figure 1. Effect of sample overlap on performance of PRS for non-heritable traits. The dotted line represents the significance threshold (*P*-value = 1x10^{-4}) for high-resolution testing in *PRSice-2* [26]. The X-axis shows the degree of overlap, calculated as $\frac{N_c}{\sqrt{N_1 N_2}}$ and the Y-axis shows the -log$_{10}$ transformed p-value of association between the PRS and the simulated phenotype. Shaded area represents the 95% confidence interval. However, as the confidence interval is small, it is difficult to observe on the scale of these plots **a)** Quantitative traits with different cohort sizes. **b)** Binary traits with population prevalence of 0.1 **c)** Binary traits with population prevalence of 0.3 **d)** Binary traits with population prevalence of 0.5

In the binary trait setting, sample overlap may be among cases only, controls only, or be among both. These alternatives were investigated by first simulating binary traits with different population prevalence using the liability threshold model [20]. Cohorts with effective sample sizes of 120k in the base data and 5000 in the target data were generated with different degrees and scenarios of sample overlap. We observed extreme inflation associated with case-only overlap when population prevalence is lower than 0.5. For a binary trait with population prevalence 0.1, a false positive rate of 40% is observed when the degree of overlap is 0.001, which corresponds to 5% of the cases from the target cohort also present in the base cohort (Fig 1b). When the degree of sample overlap is doubled (~0.002), the false positive rate is 100%. The inflation in PRS-trait

association is not as sensitive to control-only sample overlap when the population prevalence is small. We observe a false positive rate of 50% when the degree of overlap is as high as 0.092, which corresponds to 50% of the controls from the target cohort also present in the base cohort (Fig 1b). This discrepancy between the effect of case and control overlap is a result of the differential contribution of cases and controls to the PRS-trait association in our simulations. Cases are sampled from the extreme upper tail of the liability distribution at a frequency corresponding to the disease prevalence, which is typically low: this gives each case greater weight in the calculation of the PRS-trait association and, thus, an overlapping case will generate greater inflation than an overlapping control. This was consistent with our simulation results (Fig 1c, 1d), where the inflation in ΔR^2 caused by overlapping cases decreases as population prevalence increases ($\gamma = -0.245$, P-value = $1.18\text{x}10^{-9}$). The reverse relationship between inflation and population prevalence was observed for control-only overlap ($\gamma = 0.359$, p-value = $1.18\text{x}10^{-19}$). For a population prevalence of 0.5, case-only and control-only overlap have the same impact on the inflation (Fig 1d).

Given that complete overlap of individuals in the base and target data can generate PRS-trait associations that are severely inflated, closely related individuals independently enrolled into the base and target cohorts may induce some inflation considering their shared genetics and environment. Here we tested the effect of relatedness between the base and target cohorts on PRS-trait associations in non-heritable traits in a similar way to that for sample overlap (see Methods), where inter-cohort relatedness is defined as $\frac{N_r}{\sqrt{N_1 N_2}}$ where N_r is the number of samples in the target cohort that are first degree relatives with samples in the base cohort. A false positive rate of 100% is observed when the inter-cohort relatedness is 0.042 (250k base, 5k target, 30% of target samples have 1st degree relatives in the base), when the shared environment explains $\geq$30% of the trait variance. See Supplementary Fig. 2 for full results regarding the effects of inter-cohort relatedness on PRS-trait association inflation.

The analyses in this section were performed only to highlight the potential impact of sample overlap, since highly significant PRS-trait associations are observed with overlap even for non-heritable traits for which there should be no PRS-trait association. EraSOR should not be applied to data on traits for which there is little evidence of heritability, since PRS analyses performed on underpowered GWAS are more likely to generate misleading results and conclusions based on them. Therefore, as for PRS analyses in general [1], we do not recommend the application of EraSOR to base GWAS data with estimated h^2_{SNP} < 5%.

In the next section we extend these investigations to consider the effects of sample overlap on PRS-trait associations on heritable traits, but we present these findings in conjunction with results based on the application of our method EraSOR, which is designed to resolve the problem.

Performance of EraSOR

To tackle the problem of inflation caused by inter-cohort overlap and relatedness, we developed the Erase Sample Overlap and Relatedness (EraSOR) method. Using GWAS summary statistics generated from the base and target cohorts, EraSOR implements univariate and bivariate LD score regression [17,18] to estimate several parameters that are then used to perform a de-correlation calculation of the base GWAS test statistics (see Methods). These adjusted base GWAS summary statistics can then be used for downstream PRS analyses, with sample overlap or relatedness corrected for.

In order to evaluate the performance of EraSOR, we conducted an extensive set of simulations covering a range of scenarios of inter-cohort sample overlap and relatedness (see below and Methods).

Simulations using UK Biobank data

We observed that for both quantitative and binary traits, EraSOR almost entirely eliminates the inflation caused by inter-cohort overlap and relatedness in our simulations based on UK Biobank (European ancestry base and target samples) data (Figure 2). These simulations modelled a range of scenarios that varied trait heritability, prevalence, degree of overlap and combinations of overlap among cases and controls. For example, simulating quantitative traits with heritability 0.1, a base cohort of 250k samples, target cohort of 5000 samples, and degree of overlap 0.141 – in which all samples in the target data are also in the base GWAS – the mean ΔR^2 is $1.68x10^{-5}$ (standard error: $3.83x10^{-4}$) when there are 10k causal variants and 2.21 $x10^{-4}$ (standard error: $4.77x10^{-4}$) when there are 100k causal variants. Left unadjusted, the mean ΔR^2 is approximately 0.35 (standard error 0.00143) and 0.36 (standard error: 0.00141), respectively, suggesting that EraSOR has removed the inflation introduced by sample overlap. A similar pattern of complete removal of the effects of sample overlap is observed for the quantitative traits across the full range of heritability and cohort sample sizes tested (Figure 2a), with the exception that when the target cohort is of similar size as the base cohort and when majority of the target samples were found in the base GWAS, EraSOR adjusted PRS results can deviate from the truth. For example, simulating quantitative traits with heritability 0.1, a base cohort size of 120k, target cohort of 50k samples, and when all samples in the target are found in the base GWAS, the mean ΔR^2 for the adjusted PRS can be as high as 0.0134 (standard error = $3.73x10^{-4}$), which is still far closer to the truth than the unadjusted PRS (mean ΔR^2 of 0.537). We argue that in such scenarios, while EraSOR cannot completely remove the biases, it can still be used as a tool for sensitivity analyses, where a large discrepancy in performance between the adjusted and unadjusted PRS suggests there might be a large degree of overlap~~s~~. .

~~We performed real data validation of our simulations using height, body mass index (BMI) and low-density lipoprotein (LDL) data from the UK Biobank (see Supplementary methods). In all scenarios, EraSOR completely removed the effects of sample overlap, albeit with slight over-correction in Height and BMI~~

Formatted: Heading 3

Formatted: Superscript

Commented [CSW1]: We have higher variance this time.

Formatted: Superscript

~~(Supplementary Figure 2 and Supplementary Table 2). Therefore, we expect EraSOR to perform as predicted by our simulations when applied in real data settings.~~

EraSOR also performs extremely well for binary traits in our simulations (Figure 2b, 2c, 2d). In binary traits with heritability 0.1, population prevalence 0.1, a base cohort of with 120k effective samples, a target cohort of 5,000 effective samples, and with 50% of the target data presented in the base GWAS, the mean ΔR^2 for the adjusted PRS in relation to case-only overlap is 7.52×10^{-5} (standard error = 2.76×10^{-4}) (Fig 2b) and the mean ΔR^2 for the adjusted PRS in relation to control-only overlap is 3.36×10^{-4} (standard error = 2.84×10^{-4}) (Fig 2c). On the other hand, when unadjusted, the mean ΔR^2 in relation to case only overlap is as high as 0.161 (standard error = 0.00110), whereas the mean ΔR^2 is 5.80×10^{-3} (standard error = 3.48×10^{-4}) in relation to control-only overlap.

While EraSOR effectively eliminates inflation caused by inter-cohort overlap in all simulation scenarios tested in relation to heritable traits, false-positive results are still observed after EraSOR adjustment in non-heritable traits when there is a large degree of overlap (> 0.289). For non-heritable quantitative traits with base cohorts of 120k samples and target cohorts of 10k samples, if all target samples are also present in the base cohort, then we observe a false-positive rate of 20%, with a mean ΔR^2 of 5.84×10^{-4} (standard error = 1.03×10^{-4}). This is likely caused by the fact that a key component of the mathematics underlying the EraSOR algorithm (described by Eq. 11 in Methods) includes an estimate of h^2 in its denominator. Therefore, when the trait is non-heritable, Eq 9 may be unstable and lead to an error in the ~~ErasOR~~ EraSOR adjustment. However, we recommend that polygenic risk score analyses should not be performed on traits with estimated $h^2 < 0.05$ (see [1]) and, thus, in sufficiently powered applications of PRS, EraSOR should have strong performance.

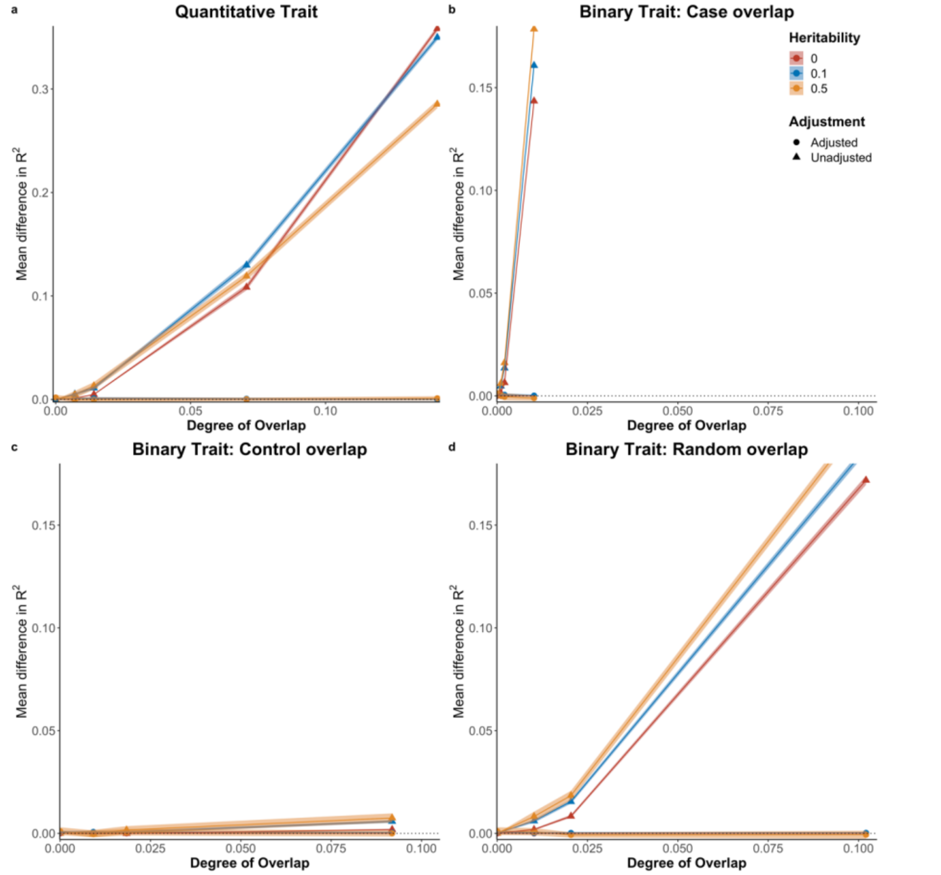

Figure 2. Comparing the performance of the PRS using the EraSOR adjusted summary statistics and the unadjusted summary statistics. The X-axis shows the degree of overlap, and the Y-axis shows the mean difference between the observed R^2 and the expected R^2. Shaded area represents the 95% confidence interval (small on this scale). **a)** Performance in quantitative traits with 250,000 samples in the base cohort and 5,000 samples in the target cohort; b) Performance in binary traits with prevalence of 0.1 and where overlap samples were ascertained for cases; **c)** ascertained for controls; **d)** or were randomly ascertained.

One of the main assumptions of EraSOR is that there is no environmental stratification ($\sigma_S^2 = 0$). To investigate the robustness of EraSOR to model misspecification, we also performed simulations by incorporating UK Biobank samples with non-European ancestry and simulated different level of environmental stratification.

Overall, EraSOR appears to be reasonably~~is~~ robust ~~against~~ to model misspecification. For example, considering the high F_{ST} between the European and non-European samples (see Methods) in UK Biobank ($F_{ST} = 0.018$), coupled~~couple~~ with a high environmental stratification (e.g. σ_S^2 =0.3), the mean ΔR^2 is still 5.58×10^{-6} with standard error of 9.11×10^{-4}, for quantitative traits with heritability of 0.1, ~~For example, for quantitative traits with heritability of 0.1,~~ a base cohort of 250k samples, target cohort of 5000 samples, and

Formatted: Subscript

Formatted: Subscript

Formatted: Not Superscript/ Subscript

a degree of overlap of 0.141.and an environmental stratification between the European and non-European UK Biobank samples at 0.3, the mean ΔR^2 is $5.58x10^{-6}$ with standard error of $9.11x10^{-4}$, EraSOR performs equally well for quantitative traits with different heritability, different levels of environmental stratifications and cohorts with different sample size and overlap (see Supplementary Fig. 4 75-6).

Real UK Biobank phenotype analysis

We performed real data validation of our simulations using height, body mass index (BMI) and low-density lipoprotein (LDL) data from the UK Biobank. In all scenarios, EraSOR completely removed the effects of sample overlap, albeit with slight over-correction in Height and BMI (Supplementary Figure 3 and Supplementary Table 2). Therefore, we expect EraSOR to perform as predicted by our simulations when applied in real data settings.

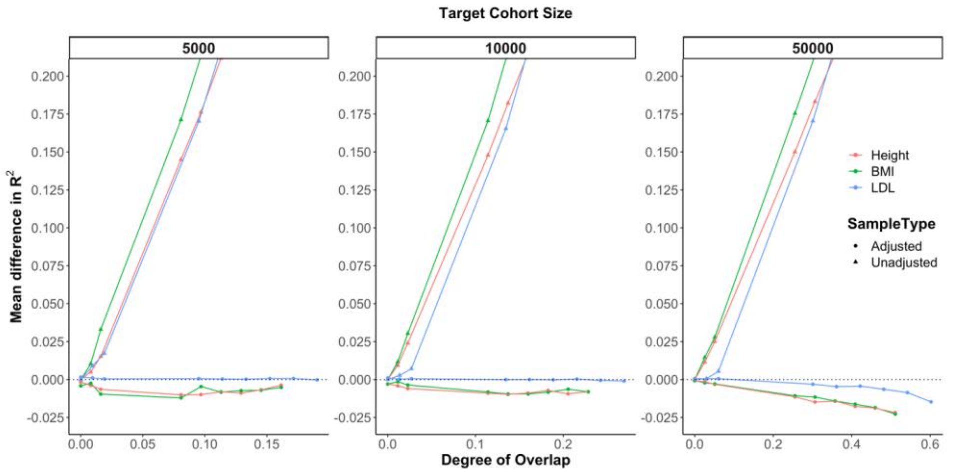

Figure 3. Comparing the performance of PRS using the EraSOR adjusted summary statistics and the unadjusted summary statistics in real UK Biobank phenotypes. The X-axis shows the degree of overlap, and the Y-axis shows the mean difference between the observed R^2 and the expected R^2. Mean difference in R^2 = 0 is represented by the black dotted line. Each column corresponds to different target cohort size and different colors correspond to different traits. Performance of the adjusted PRS is indicated with circle and performance of the unadjusted PRS is indicated with triangle. Shaded area represents the 95% confidence interval, which tends to be small.Robustness with diverse ancestry data

While the F_{ST} between the European and non-European samples (see Methods) is high (F_{ST} = 0.018), non-European samples account for only ~5% of the UK Biobank population. Our results might therefore be

dominated by the European samples. To understand how EraSOR performs in a more heterogenous dataset, additional simulations were performed using HapGen2 simulated genotype [22].

Using HapGen2 and the Finnish and Yoruban recombination maps from 1000 genome, we simulated 180k “Finnish” and 180k “Yoruban” samples. F_{ST} estimation from PLINK between the two population is only 0.00639, much lower than the reported $F_{ST} > 0.1$ African and European population[23]. This discrepancy is likely a result of the fact that the only difference in the simulations is the recombination map used (Supplementary Fig. 8) by HapGen2. Nonetheless, the HapGen2 simulation generates a dataset where the genetic signal is not dominated by one single population and allows us to better understand the performance of EraSOR in the presence of stratification.

Under the HapGen2 simulations, for quantitative traits with heritability 0.1, base cohort size of 250k, target cohort size of 5000, degree of overlap at 0.141 (all target samples also in the base cohort) and without environmental stratification, then the mean ΔR^2 for the adjusted PRS is -1.92×10^{-4} (standard error = 5.58×10^{-4}) compared to the mean ΔR^2 of 0.243 (standard error = 0.00152); when the environmental stratification is 0.3, the mean ΔR^2 of the adjusted PRS is 5.70×10^{-5} (standard error = 8.19×10^{-4}), much smaller than the mean ΔR^2 of 0.213 from the unadjusted PRS.

One potential reason for the robustness of EraSOR may be due to the simplistic population structure of the simulated genotype. As we simulated the environmental stratification according to the population label, it is possible that by adjusting for PCs, the environmental stratification was fully adjusted for. While it is highly unlikely that environmental stratification is orthogonal to population genetic structure, we performed an additional simulation in which the population label was randomly assigned to the simulated genotype. This ensured the simulation of environmental stratification independent of population genetic structure and, thus, should not be capture by PCA adjustment (see Supplementary Methods and Supplementary Fig. 9).

Even when environmental stratification is simulated independently of the population genetic structure, EraSOR adjustments are still robust to different environmental structure. For quantitative traits with heritability of 0.1, base cohort size of 250k, target cohort size of 5,000, environmental stratification of 0.3 and degree of overlap of 0.141, the mean ΔR^2 for the adjusted PRS is -3.11×10^{-4} (standard error = 8.00×10^{-4}).

Discussion

The recent advent of large-scale national and regional biobank projects, such as the UK Biobank [8], Japan Biobank [9] and FinnGen [11], have provided large resources of genotype-phenotype data ideal for conducting polygenic risk score analyses. However, this burgeoning generation of large data has led to an increased risk of inter-cohort sample overlap or relatedness, which can lead to inflated type 1 error. Due to privacy laws and practical concerns, it is usually impossible to identify overlapping samples or related samples across different cohorts. However, ideally researchers would be aware of the scale of the potential problem and have tools to mitigate against it. Therefore, here we reported on an investigation to evaluate the impact of inter-cohort sample overlap and relatedness in PRS analyses and developed a method to account for potential inter-cohort overlap and relatedness that does not require access to raw genotype data from the base GWAS.

We demonstrated that inter-cohort overlap results in a significant and often substantial inflation in the observed PRS-trait association, coefficient of determination (R^2) and false-positive rate. This inflation can be high even when the absolute number of overlapping individuals is small if this makes up a notable fraction of the target samples. The inflation is noticeably more severe for binary traits with a small population prevalence when all the overlapping samples are cases. Therefore, PRS results will likely be misinterpreted unless inter-cohort sample overlap and close relatedness is properly accounted for.

Here, we developed the Erase Sample Overlap and Relatedness (EraSOR) method. EraSOR is designed to correct for inter-cohort sample overlap and relatedness using only summary statistics, without requiring any other information. The results of PRS analyses using EraSOR-adjusted GWAS results in the presence of sample overlap or relatedness was remarkably similar to those gained when the overlap was explicitly removed in most simulated conditions. EraSOR is also robust to mis-specification of the model, for example, when there is environmental stratification. While EraSOR does not fully adjust for the bias introduced by inter-cohort overlap for non-heritable traits when the degree of overlap is high, we recommend that researchers should not perform PRS analyses on non-heritable traits in any case [1]. EraSOR performs well for the majority of simulation scenarios tested here, which we believe reflect a large fraction of PRS studies.

Theoretically, as ρ from the bivariate LD score regression is assumed to be the phenotypic correlation [18], we can apply EraSOR in situations where the base and target cohorts measure different phenotypes. Based on LeBlanc's equations [16], the spurious correlations caused by inter-cohort overlap and relatedness is a function of the phenotypic correlation. While this suggests that the impact of inter-cohort overlap and relatedness are likely to be smaller for cross-trait analyses, EraSOR adjustments may still be beneficial in these scenarios. Investigation of the performance of EraSOR in cross-trait analyses should be the subject of future work. Further research is also required to understand the performance and biases of EraSOR for applications in cross-trait studies and in its potential application to GWAS meta-analyses.

Our algorithm is not without any limitations. First, as EraSOR depends on the LD score intercept estimates for the adjustment, all assumptions of LD score regression also apply to EraSOR. For example, LD score regression assumes that the level of genetic and environmental stratification is similar between the two cohorts [13], and if this assumption is violated, then it is likely that the bivariate LD score equation does not hold, which will lead to bias in EraSOR estimates. We have also only tested EraSOR in the real data of the UK Biobank, which has a maximum genetic stratification corresponding to an F_{ST} of approximately 0.02 (between European and non-European ancestry samples), and, thus, we do not recommend applying EraSOR to data for which the F_{ST} within or between base and target data is greater than 0.02. In practical terms, this means that until further testing or development of EraSOR has been performed, then EraSOR should only be applied to single-ancestry base and target data sets that are closely matched by ancestry to each other. As it happens, cohorts of highly different ancestry are less likely to involve sample overlap. Furthermore, all testing performed here is based on the C+T PRS calculation method, and so is subject to its limitations, such as potential overfitting of PRS-trait associations. However, given the similar performance of different PRS methods [29] we do not expect qualitatively different results to those observed here when EraSOR is applied to correct for sample overlap in PRS analyses using other PRS methods. Moreover, due to reliance on LD score regression estimates, EraSOR only produces sufficiently accurate adjustments for application when both base and target cohorts have sample sizes greater than 1,000 and is only consistently accurate when both cohorts have are greater than 5,000 samples. In addition, when the target cohort size is similar to the base cohort size, and all the target samples are present were found in the base cohort, EraSOR can failstruggle to fully adjusted for the inflation caused by sample overlap. HoweverNonetheless, even in this extremein such scenario, the EraSOR adjusted results are still farmuch closer to the empirical truth than the unadjusted PRS results. In general, in fact, EraSOR tends to produce an over-correction, meaning that PRS-trait associations are underestimated after EraSOR-adjustment. However, despite its limitations, EraSOR is an ideal tool for application in settings in which there is known

~~overlap in relation to large target samples and for sensitivity analyses in PRS studies. If the performance of PRS using the unadjusted and EraSOR adjusted summary statistics differs~~a~~A~~ large discrepancy between the unadjusted and EraSOR-adjusted results ~~substantially, then~~ should~~can~~ ~~this will~~ act as a warning as to the possible presence of inter-cohort overlap or close relatedness, which ~~that~~ should ~~be~~ either be removed, or adjusted for using EraSOR, in order to obtain more reliable PRS analysis results.

Availability of supporting source code and requirements

Project Name	EraSOR
Project Homepage	https://choishingwan.gitlab.io/EraSOR/
Programming Language	Python (version 3.0+)
License	GNU General Public License version 3.0 (GPLv3)
Any restrictions to use by non-academics	None
Simulation Scripts	**https://gitlab.com/choishingwan/sample_overlap_paper**

Availability of supporting data and materials

All code used for this paper is available at **https://gitlab.com/choishingwan/sample_overlap_paper** and were implemented using nextflow (version 20.10.0 build 5430) [28]. Full source code and documentation of EraSOR can be found on **https://choishingwan.gitlab.io/EraSOR/**

Abbreviations

PRS: polygenic risk score, GWAS: Genome Wide Association Study

Additional files

Supplementary Table 1: Simulation results

Supplementary Table 2: Results for real data analyses

Supplementary Method and Figures

Competing interests

The authors declare that they have no competing interests.

Funding

Medical Research Council FundRef identification ID: **http://dx.doi.org/10.13039/501100000265** MR/N015746/1 and the National Institute of Health (R01MH122866) to P.F.O. This report represents independent research partially funded by the National Institute for Health Research (NIHR) Biomedical Research Centre at South London and Maudsley NHS Foundation Trust and King's College London. Research reported in this paper was supported by the Office of Research Infrastructure of the National Institutes of Health under award number S10OD026880. The content is solely the responsibility of the authors and does not necessarily represent the official views of the National Institutes of Health, NHS, the NIHR or the Department of Health.

Authors' contributions

Conceptualization, S.W.C. and P.F.O.; Methodology, S.W.C., T.S.H.M., C.H. and P.F.O.; Investigation, S.W.C.; Software, S.W.C.; Supervision, P.F.O.; Funding Acquisition, P.F.O.; Writing – Original Draft, S.W.C; Writing - Review and Edition, S.W.C., C.H. and P.F.O.;

Acknowledgements

We thank the participants in the UK Biobank and the scientists involved in the construction of this resource. We thank Jonathan Coleman and Kylie Glanville for helpful discussions. This research has been conducted using the UK Biobank Resource under application 18177 (P.F.O.). This work was supported in part through the computational resources and staff expertise provided by Scientific Computing at the Icahn School of Medicine at Mount Sinai.

Reference

1. Choi SW, Mak TS-H, O'Reilly PF. Tutorial: a guide to performing polygenic risk score analyses. *Nat Protoc*. 2020; doi: 10.1038/s41596-020-0353-1.

2. Mavaddat N, Michailidou K, Dennis J, Lush M, Fachal L, Lee A, et al.. Polygenic Risk Scores for Prediction of Breast Cancer and Breast Cancer Subtypes. *Am J Hum Genet*. 2019; doi: 10.1016/j.ajhg.2018.11.002.

3. Zhang J-P, Robinson D, Yu J, Gallego J, Fleischhacker WW, Kahn RS, et al.. Schizophrenia Polygenic Risk Score as a Predictor of Antipsychotic Efficacy in First-Episode Psychosis. *Am J Psychiatry*. 2019; doi: 10.1176/appi.ajp.2018.17121363.

4. Natarajan P, Young R, Stitziel NO, Padmanabhan S, Baber U, Mehran R, et al.. Polygenic Risk Score Identifies Subgroup with Higher Burden of Atherosclerosis and Greater Relative Benefit from Statin Therapy in the Primary Prevention Setting. *Circulation*. 2017; doi: 10.1161/CIRCULATIONAHA.116.024436.

5. Mega JL, Stitziel NO, Smith JG, Chasman DI, Caulfield MJ, Devlin JJ, et al.. Genetic risk, coronary heart disease events, and the clinical benefit of statin therapy: an analysis of primary and secondary prevention trials. *The Lancet*. Elsevier; 2015; doi: 10.1016/S0140-6736(14)61730-X.

6. Pain O, Hodgson K, Trubetskoy V, Ripke S, Marshe VS, Adams MJ, et al.. Antidepressant Response in Major Depressive Disorder: A Genome-wide Association Study. *medRxiv*. Cold Spring Harbor Laboratory Press; 2020; doi: 10.1101/2020.12.11.20245035.

7. Dudbridge F. Power and Predictive Accuracy of Polygenic Risk Scores. *PLOS Genet*. 2013; doi: 10.1371/journal.pgen.1003348.

8. Sudlow C, Gallacher J, Allen N, Beral V, Burton P, Danesh J, et al.. UK Biobank: An Open Access Resource for Identifying the Causes of a Wide Range of Complex Diseases of Middle and Old Age. *PLOS Med*. 2015; doi: 10.1371/journal.pmed.1001779.

9. Nagai A, Hirata M, Kamatani Y, Muto K, Matsuda K, Kiyohara Y, et al.. Overview of the BioBank Japan Project: Study design and profile. *J Epidemiol*. 2017; doi: 10.1016/j.je.2016.12.005.

10. Fan C-T, Lin J-C, Lee C-H. Taiwan Biobank: a project aiming to aid Taiwan's transition into a biomedical island. *Pharmacogenomics*. 2008; doi: 10.2217/14622416.9.2.235.

11. FinnGen. FinnGen Documentation of R3 release.

12. Sullivan PF, Agrawal A, Bulik CM, Andreassen OA, Børglum AD, Breen G, et al.. Psychiatric Genomics: An Update and an Agenda. *Am J Psychiatry*. 2018; doi: 10.1176/appi.ajp.2017.17030283.

13. Yengo L, Sidorenko J, Kemper KE, Zheng Z, Wood AR, Weedon MN, et al.. Meta-analysis of genome-wide association studies for height and body mass index in ∼700000 individuals of European ancestry. *Hum Mol Genet*. 2018; doi: 10.1093/hmg/ddy271.

14. Global Lipids Genetics Consortium, Willer CJ, Schmidt EM, Sengupta S, Peloso GM, Gustafsson S, et al.. Discovery and refinement of loci associated with lipid levels. *Nat Genet*. 2013; doi: 10.1038/ng.2797.

15. Wray NR, Yang J, Hayes BJ, Price AL, Goddard ME, Visscher PM. Pitfalls of predicting complex traits from SNPs. *Nat Rev Genet*. 2013; doi: 10.1038/nrg3457.

16. LeBlanc M, Zuber V, Thompson WK, Andreassen OA, Frigessi A, Andreassen BK, et al.. A correction for sample overlap in genome-wide association studies in a polygenic pleiotropy-informed framework. *BMC Genomics*. 2018; doi: 10.1186/s12864-018-4859-7.

17. Bulik-Sullivan BK, Loh P-R, Finucane HK, Ripke S, Yang J, Schizophrenia Working Group of the Psychiatric Genomics Consortium, et al.. LD Score regression distinguishes confounding from polygenicity in genome-wide association studies. *Nat Genet*. 2015; doi: 10.1038/ng.3211.

18. Bulik-Sullivan B, Finucane HK, Anttila V, Gusev A, Day FR, Loh P-R, et al.. An atlas of genetic correlations across human diseases and traits. *Nat Genet*. 2015; doi: 10.1038/ng.3406.

19. Choi SW. GreedyRelated: Script for greedily remove related samples.

20. Falconer DS. Introduction to quantitative genetics. New York,: Ronald Press Co;

21. Willer CJ, Li Y, Abecasis GR. METAL: fast and efficient meta-analysis of genomewide association scans. *Bioinformatics*. 2010; doi: 10.1093/bioinformatics/btq340.

22. Su Z, Marchini J, Donnelly P. HAPGEN2: simulation of multiple disease SNPs. *Bioinforma Oxf Engl*. 2011; doi: 10.1093/bioinformatics/btr341.

23. Auton A, Abecasis GR, Altshuler DM, Durbin RM, Abecasis GR, Bentley DR, et al.. A global reference for human genetic variation. *Nature*. Nature Publishing Group; 2015; doi: 10.1038/nature15393.

24. Abraham G, Qiu Y, Inouye M. FlashPCA2: principal component analysis of Biobank-scale genotype datasets. *Bioinformatics*. 2017; doi: 10.1093/bioinformatics/btx299.

25. Chang CC, Chow CC, Tellier LC, Vattikuti S, Purcell SM, Lee JJ. Second-generation PLINK: rising to the challenge of larger and richer datasets. *GigaScience*. 2015; doi: 10.1186/s13742-015-0047-8.

26. Choi SW, O'Reilly PF. PRSice-2: Polygenic Risk Score software for biobank-scale data. *GigaScience*. 2019; doi: 10.1093/gigascience/giz082.

27. Euesden J, Lewis CM, O'Reilly PF. PRSice: Polygenic Risk Score software. *Bioinformatics*. 2015; doi: 10.1093/bioinformatics/btu848.

28. Di Tommaso P, Chatzou M, Floden EW, Barja PP, Palumbo E, Notredame C. Nextflow enables reproducible computational workflows. *Nat Biotechnol*. 2017; doi: 10.1038/nbt.3820.

29. Pain O, Glanville KP, Hagenaars SP, Selzam S, Furtjes AE, Gaspar HA, et al.. Evaluation of polygenic prediction methodology within a reference-standardized framework. *Plos Gen.* 2021. doi: 10.1371/journal.pgen.1009021

Click here to access/download
Supplementary Material
Supplementary_101822.docx

Supplementary Table 1

Click here to access/download
Supplementary Material
Supplementary_table1.csv

Supplementary Table 2

Click here to access/download
Supplementary Material
Supplementary_table2.csv

Cover Letter
Click here to access/download;Personal Cover;CoverLetter_Response.docx

Icahn School of Medicine at Mount Sinai

Dr Paul F. O'Reilly
Dept. Genetics and Genomic Sciences
Icahn Institute at Mount Sinai
One Gustave L. Levy Place, Box 1498
New York, NY 10029-6574

EraSOR: a software tool to eliminate inflation caused by sample overlap in polygenic score analyses

17th October 2022

Dear Editor

We apologize for the delay in response to reviews of our manuscript. We thank the reviewers for their feedback, which has helped us to produce a substantially improved manuscript (this, despite our 1st author, Shing Wan Choi, moving from academia to an industry position soon after we received the reviews).

We have contributed additional analyses to further support our results, we have revised and added text throughout the manuscript to make our presentation of our methodology and results clearer and to clarify multiple points raised, and we have produced a detailed user guide for our EraSOR software, with test data sets included, to provide a user-friendly software for optimal use in the field. On the latter point in particular, we have developed EraSOR with the same user-friendly ethos as our popular PRSice-2 software, published in *Gigascience* in 2019 with > 550 citations to date. We believe that our EraSOR software may prove similarly impactful as PRSice-2, given that there is good reason to use EraSOR in *all* standard PRS analyses, at least as a sensitivity tool, because it is rarely known for certain that a PRS analysis is not subject to sample overlap and, therefore, EraSOR can confirm (or otherwise) the validity of results.

We have enclosed a point-by-point response to the reviews and hope that you now find our article suitable for publication in *Gigascience* and we look forward to hearing from you

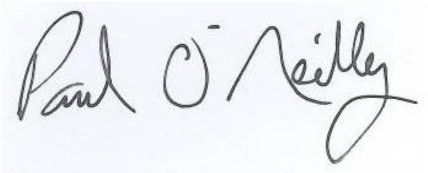

Paul F. O'Reilly and Shing Wan Choi
