# Supplementary material for: EraSOR: a software tool to eliminate inflation caused by sample overlap in polygenic score analyses
Source: Gigascience. 2023 Jun 16;12:giad043. doi: 10.1093/gigascience/giad043 (PMC10273836; doi:10.1093/gigascience/giad043)
Supplement: giad043_GIGA-D-22-00019_Revision_3 [file giad043_giga-d-22-00019_revision_3.pdf]

GigaScience

EraSOR: a software tool to eliminate inflation caused by sample overlap in polygenic score analyses

--Manuscript Draft--

**Manuscript Number:**	GIGA-D-22-00019R3
**Full Title:**	EraSOR: a software tool to eliminate inflation caused by sample overlap in polygenic score analyses
**Article Type:**	Technical Note
**Funding Information:**	National Institutes of Health (R01MH122866) — Dr Paul F. O’Reilly
**Abstract:**	Polygenic risk score (PRS) analyses are now routinely applied across biomedical research. However, as PRS studies grow in size, there is an increased risk of sample overlap between the genome-wide association study (GWAS) from which the PRS is derived and the ‘target sample’, in which PRS are computed and hypotheses are tested. Despite the wide recognition of the sample overlap problem, its potential impact on the results from PRS studies has not yet been quantified and no analytical solution has been provided. Here, we first conduct a comprehensive investigation into the scale of the sample overlap problem, finding that PRS results can be substantially inflated even in the presence of minimal overlap. Next, we introduce a method and software, EraSOR (Erase Sample Overlap and Relatedness), which eliminates the inflation caused by sample overlap (and close relatedness) in almost all settings tested here. EraSOR could be useful in PRS studies (with target sample > 1k) similar to those investigated here, either: (i) to mitigate the potential effects of known or unknown inter-cohort overlap and close relatedness, or (ii) as a sensitivity tool to highlight the possible presence of sample overlap before its direct removal, when possible, or else to provide a lower bound on PRS analysis results after accounting for potential sample overlap.
**Corresponding Author:**	Shing Wan Choi<br>Icahn School of Medicine at Mount Sinai<br>New York, UNITED STATES
**Corresponding Author Secondary Information:**	
**Corresponding Author's Institution:**	Icahn School of Medicine at Mount Sinai
**Corresponding Author's Secondary Institution:**	
**First Author:**	Shing Wan Choi
**First Author Secondary Information:**	
**Order of Authors:**	Shing Wan Choi
	Timothy Shin Heng Mak
	Clive J. Hoggart
	Paul F. O’Reilly
**Order of Authors Secondary Information:**	
**Response to Reviewers:**	Attached are the updated manuscript with the required correction to citations. Thank you.
**Additional Information:**	
**Question**	**Response**
Are you submitting this manuscript to a special series or article collection?	No
**Experimental design and statistics**	Yes

Powered by Editorial Manager® and ProduXion Manager® from Aries Systems Corporation

Full details of the experimental design and statistical methods used should be given in the Methods section, as detailed in our Minimum Standards Reporting Checklist. Information essential to interpreting the data presented should be made available in the figure legends.<br><br>Have you included all the information requested in your manuscript?	
**Resources**<br><br>A description of all resources used, including antibodies, cell lines, animals and software tools, with enough information to allow them to be uniquely identified, should be included in the Methods section. Authors are strongly encouraged to cite Research Resource Identifiers (RRIDs) for antibodies, model organisms and tools, where possible.<br><br>Have you included the information requested as detailed in our Minimum Standards Reporting Checklist?	Yes
**Availability of data and materials**<br><br>All datasets and code on which the conclusions of the paper rely must be either included in your submission or deposited in publicly available repositories (where available and ethically appropriate), referencing such data using a unique identifier in the references and in the "Availability of Data and Materials" section of your manuscript.<br><br>Have you have met the above requirement as detailed in our Minimum Standards Reporting Checklist?	Yes

Manuscript

Click here to access/download;Manuscript;erasor_for_publication.docx

EraSOR: a software tool to eliminate inflation caused by sample overlap in polygenic score analyses

Shing Wan Choi [1,2*], Timothy Shin Heng Mak [3], Clive J. Hoggart [1], Paul F. O'Reilly [1,2*]

1 Department of Genetics and Genomic Sciences, Icahn School of Medicine, Mount Sinai, 1 Gustave L. Levy Pl, New York City, NY 10029, USA;

2 MRC Social, Genetic and Developmental Psychiatry Centre, Institute of Psychiatry, Psychology and Neuroscience, King's College London, De Crespigny Park, Denmark Hill, London, UK, SE5 8AF; and

3 Centre of Genomic Sciences, University of Hong Kong, 21 Sassoon Road, Pokfulam, Hong Kong SAR, China

*To whom correspondence should be addressed

ORCID iDs: Shing Wan Choi [0000-0003-2215-3238]; Timothy Shin Heng Mak [0000-0002-2151-2406]; Clive J Hoggart [0000-0002-6875-6993]; Paul F O'Reilly [0000-0001-7515-0845];

Abstract

Background: Polygenic risk score (PRS) analyses are now routinely applied across biomedical research. However, as PRS studies grow in size, there is an increased risk of sample overlap between the genome-wide association study (GWAS) from which the PRS is derived and the 'target sample', in which PRS are computed and hypotheses are tested. Despite the wide recognition of the sample overlap problem, its potential impact on the results from PRS studies has not yet been quantified and no analytical solution has been provided. Findings: Here, we first conduct a comprehensive investigation into the scale of the sample overlap problem, finding that PRS results can be substantially inflated even in the presence of minimal overlap. Next, we introduce a method and software, EraSOR (Erase Sample Overlap and Relatedness), which eliminates the inflation caused by sample overlap (and close relatedness) in almost all settings tested here. Conclusions: EraSOR could be useful in PRS studies (with target sample > 1k) similar to those investigated here, either: (i) to mitigate the potential effects of known or unknown inter-cohort overlap and close relatedness, or (ii) as a sensitivity tool to highlight the possible presence of sample overlap before its direct removal, when possible, or else to provide a lower bound on PRS analysis results after accounting for potential sample overlap.

Introduction

Polygenic risk scores (PRSs) are proxies of individuals' genetic liability to a trait or disease [1] that have been applied in a range of research settings, including patient stratification [2] and investigation of treatment response [3–6]. The power of PRS analyses that test a study hypothesis is dependent on the heritability and polygenicity of the trait, the power of the genome wide association study (GWAS) used to derive the PRS, and the size of the target data sample used to test the hypothesis [7]. The recent surge in availability of high quality genotype-phenotype data from large-scale biobank projects, such as the UK Biobank [8], BioBank Japan [9], Taiwan Biobank [10], and FinnGen [11], as well as GWAS resources from large consortia such as the Psychiatric Genomic Consortium (PGC) [12], GIANT [13] and the Global Lipids Genetics Consortium (GLGC) [14], have provided unprecedented opportunities to perform highly-powered PRS analyses.

However, expansion in data sizes comes with a cost in this setting: as sample sizes increase, it is more likely that samples are recruited into multiple cohorts or that entire cohorts are included in multiple consortia. For PRS analyses, which typically test for association between PRS and a trait(s) or outcome of interest, overlapping samples between the GWAS and target data samples can result in spurious inflation of the coefficient of determination (R^2) and association P-values, leading to false-positive and exaggerated findings [15]. If an entire cohort is present in the base GWAS and target data, then ideally it should be removed as follows: (i) directly remove the cohort from the GWAS data (recompute base GWAS results), (ii) if (i) is not an option, then use the cohort GWAS results to derive the base GWAS results minus the cohort, an analytical solution of which we have previously described, (iii) remove cohort from the target data directly if doing so does not compromise power. Likewise, overlapping individuals should ideally be removed from either the GWAS or target data to avoid misinterpretation of results, but participant privacy agreements usually limit access to raw genotyping data, meaning that this is generally not an option.

Here we first evaluate the extent to which different degrees of sample overlap and relatedness between GWAS and target samples generates biased PRS-trait associations. Next, to overcome the sample overlap problem, we develop and introduce EraSOR (Erase Sample Overlap and Relatedness), a software that adjusts GWAS summary statistics [1] to correct for inflation of PRS-trait association results caused by overlapping samples between the GWAS and target samples. Through extensive simulations using the UK Biobank genetic data [8], we demonstrate that EraSOR can robustly adjust for inflation in test statistics caused by various degrees of overlapping samples and close relatedness, under different ascertainment schemes in case/control settings. EraSOR should increase the accuracy of results in all future PRS studies with known sample overlap and will act as a critical sensitivity tool as part of PRS analyses to ensure the reliability of results in PRS studies with unknown but potential sample overlap. EraSOR is an open-source software and is freely available [16].

Methods

EraSOR framework

Consider two GWAS $k = \{1, 2\}$ performed on the same continuous outcome Y_k. The effect size of the gth SNP in study k (β_{kg}) is estimated using a regression model

$$Y_k = \alpha_{kg} + \beta_{kg} X_{kg} + \varepsilon_{kg} \quad (1)$$

where X_{kg} is the standardized genotype vector for SNP g in study k, and ε_{kg} is the random error assumed to be independent between studies. Under the null model of no contribution of SNP g to the trait, $\beta_{kg} =$

0, and assuming no sample overlap, then $\widehat{\beta_{1g}}$ and $\widehat{\beta_{2g}}$ estimated from the two GWASs should be independent, i.e., $cor(\widehat{\beta_{1g}}, \widehat{\beta_{2g}}) = 0$. However, when there are overlapping samples between the two studies, then a correlation is induced between the regression coefficients, such that $cor(\widehat{\beta_{1g}}, \widehat{\beta_{2g}}) \neq 0$. From LeBlanc et al [17], this correlation can be approximated as

$$cor(\widehat{\beta_{1g}}, \widehat{\beta_{2g}}) \approx \frac{N_c}{\sqrt{N_1 N_2}} cor(Y_1, Y_2) \quad (2)$$

for quantitative traits, where $cor(Y_1, Y_2)$ represents the correlation between the traits; N_c is the number of overlapping samples; and N_1, N_2 are the sample sizes of studies 1 and 2, respectively [17]. Since we are considering only a single phenotype here, $cor(Y_1, Y_2)$ is equal to 1, and so we have:

$$cor(\widehat{\beta_{1g}}, \widehat{\beta_{2g}}) \approx \frac{N_c}{\sqrt{N_1 N_2}} \quad (3)$$

which captures correlations due only to sample overlap, independent of the true causal effect (note that if the cohorts were also identical then $N_1=N_2=N_c$ and thus, appropriately, both sides of (3) would equal 1). Assuming sample overlap does not affect the standard error estimates, LeBlanc et al [17] proposed that when the number of overlapping samples (N_c) is known, one can adjust the joint distribution of the summary statistics (z-scores) of the two GWASs as:

$$\boldsymbol{z_{de-corr}} = \boldsymbol{C^{-0.5} z} \quad (4)$$

where $\boldsymbol{z}$ is a 2-by-M matrix containing z-scores estimated in each study, M is the number of SNPs common to both studies, and $\boldsymbol{C}$ is the 2x2 matrix with ones as its diagonal elements and $cor(\widehat{\beta_{1g}}, \widehat{\beta_{2g}})$ as its off-diagonal elements. While this adjustment is effective [17], it requires prior knowledge of N_c, which is typically unknown in PRS studies. Here, we propose utilizing univariate and bivariate LD score regression [18,19] to estimate $\frac{N_c}{\sqrt{N_1 N_2}}$ and thus $cor(\widehat{\beta_{1g}}, \widehat{\beta_{2g}})$ from Eq. 3 as follows described below.

Bivariate LD score regression [19] is typically used to estimate the genetic correlation between two traits using two GWASs corresponding to each. In the formulation of bivariate LD score regression, genetic stratification – which corresponds to structure in genetic variation in a population due to non-random mating – is assumed to be similar in each GWAS sample, since making this assumption simplifies the mathematics and is approximately true in many settings. Yengo et al [13] generalized this equation by introducing Wright's F_{ST}, which measures genetic stratification, and made the simplifying assumption that genetic structure in a population is caused by the population comprising two sub-populations, with mating occurring mostly within, rather than between, the sub-populations. In this way, F_{ST} here measures the genetic differences between two sub-populations that make up the each of the study population, and the F_{ST} within each study should be the same. Yengo et al further introduced an environmental stratification term, σ_S, which is the mean phenotypic difference between the sub-populations. This leads to the following equation:

$$\mathbb{E}[z_{1j} z_{2j}] = \frac{\sqrt{N_1 N_2}\rho_g}{M} l_j + \frac{N_c \rho}{\sqrt{N_1 N_2}} + \rho_g F_{ST}^2 \sqrt{N_1 N_2} + \frac{N_c^2 F_{ST} \sigma_S^2}{\sqrt{N_1 N_2}} \quad (5)$$

where l_j is the 'LD score' defined as the sum of pairwise squared correlations between genotypes at SNP j and all other SNPs within the same LD block; ρ_g is the genetic covariance between the two traits; $\rho = \rho_g + \rho_e$; ρ_e is the non-genetic covariance; F_{ST} and σ_s are the genetic and environmental stratification respectively [13]. Since we are considering only a single phenotype here, ρ is equal to 1, and so we have:

$$\mathbb{E}\left[z_{1j}z_{2j}\right] = \frac{\sqrt{N_1N_2}\rho_g}{M}l_j + \frac{N_c}{\sqrt{N_1N_2}} + \rho_g F_{ST}^2\sqrt{N_1N_2} + \frac{N_c^2F_{ST}\sigma_S^2}{\sqrt{N_1N_2}} \quad (6)$$

We wish to solve for N_c and hence apply Eq. 3 to generate a de-correlated base GWAS that does not lead to inflated PRS-trait associations due to sample overlap. To do this, we will utilize the univariate LD score regression model, which can be derived as a special case of the bivariate LD score equation by assuming that the two outcomes and cohorts are identical [13,18] (i.e. $N_1 = N_2 = N_c = N$), leading to:

$$\mathbb{E}\left[\chi_j^2\right] = \frac{Nh^2}{M}l_j + 1 + NF_{ST}\left(h^2F_{ST} + \sigma_S^2\right) \quad (7)$$

Univariate LD score regression performs a regression of observed χ^2 on l_j, with the effect size estimate of l_j corresponding to a scaled estimate of heritability ($\widehat{h_i^2}$) and with the estimated intercept term, $\widehat{I_u}$ as follows:

$$\widehat{I_u} = 1 + N_iF_{ST}\left(\widehat{h_i^2}F_{ST} + \sigma_S^2\right)$$

A key observation by Yengo et al [13] is that in addition to the level of sample overlap, the inflation of the bivariate LD score regression intercept is also affected by the level of genetic and environmental stratification. As such, the Bivariate LD score regression intercept cannot directly be used as an estimate of the level of sample overlap. However, if we assume that the environmental stratification $\sigma_S^2 = 0$, then we have:

$$\begin{aligned}
\widehat{I_u} &= 1 + N_iF_{ST}\left(\widehat{h_i^2}F_{ST} + \sigma_S^2\right) \\
\widehat{I_u} &= 1 + N_iF_{ST}^2\widehat{h_i^2} \\
F_{ST}^2 &= \frac{\widehat{I_u} - 1}{N_i\widehat{h_i^2}}
\end{aligned} \quad (8)$$

Since we can estimate F_{ST}^2 using both the base and target data, we can take the weighted mean estimate of both:

$$\widehat{F_{ST}^2} = \frac{1}{N_1 + N_2}\sum_{i=1}^{2}\frac{\widehat{I_i} - 1}{\widehat{h_i^2}} \quad (9)$$

The intercept term of the bivariate LD score regression is:

$$\widehat{I_b} = \frac{N_c}{\sqrt{N_1N_2}} + \widehat{\rho_g}F_{ST}^2\sqrt{N_1N_2} + \frac{N_c^2F_{ST}\sigma_S^2}{\sqrt{N_1N_2}} \quad (10)$$

Substituting Eq. 9 and $\sigma_S^2 = 0$ into Eq. 10, we have:

$$\begin{aligned}
\widehat{I_b} &= \frac{N_c}{\sqrt{N_1N_2}} + \widehat{\rho_g}\widehat{F_{ST}^2}\sqrt{N_1N_2} + \frac{N_c^2 F_{ST}\sigma_S^2}{\sqrt{N_1N_2}} \\
\widehat{I_b} &= \frac{N_c}{\sqrt{N_1N_2}} + \widehat{\rho_g}\sqrt{N_1N_2}\left(\frac{1}{N_1+N_2}\sum_{i=1}^{2}\frac{\widehat{I_i}-1}{\widehat{h_i^2}}\right) \\
\frac{N_c}{\sqrt{N_1N_2}} &= \frac{\widehat{\rho_g}\sqrt{N_1N_2}}{N_1+N_2}\sum_{i=1}^{2}\frac{\widehat{I_i}-1}{\widehat{h_i^2}} - \widehat{I_b} \qquad (11)
\end{aligned}$$

Since we can estimate the genetic covariate ($\widehat{\rho_g}$), the trait heritability $\widehat{h_i^2}$ and the intercepts from the univariate and bivariate LD score regression analyses of the GWAS and target data, we can obtain an estimate of $\frac{N_c}{\sqrt{N_1N_2}}$. Substituting this estimate into Eq. 3 will derive an estimate of $cor(\widehat{\beta_{1g}}, \widehat{\beta_{2g}})$ that can be used to produce de-correlated GWAS z-statistics via Eq.4. EraSOR automatically performs the bivariate LD score and univariate LD score regression analyses on the GWAS summary statistics generated from the base and target data. Because of our assumption of $\sigma_S^2 = 0$, EraSOR may underperform when large environmental stratification is present. In addition, Eq 2 models the inflation under the null. For highly polygenic traits, a high fraction of variants across the genome may deviate from the null, thus introducing bias into EraSOR adjustments. To test the performance of EraSOR, including its robustness to the modelling assumptions (e.g., assuming $\sigma_S^2 = 0$), we performed a series of extensive simulations.

UK Biobank genotype data

The UK Biobank is a prospective cohort study of around 500,000 individuals recruited across the United Kingdom during 2006-2010. The genetic data from UK Biobank comprises 488,377 samples and 805,426 SNPs. Standard quality control (QC) procedures were performed, removing any SNPs with minor allele frequency < 0.01, genotype missingness > 0.02 and with a Hardy Weinberg Equilibrium Test *P*-value < $1\text{x}10^{-8}$. Samples with high levels of missingness or heterozygosity, with mismatching genetic-inferred and self-reported sex, or with aneuploidy of the sex chromosomes were removed as recommended by the UK Biobank data processing team. Next, 4-means clustering was applied to the first two Principal Components (PCs) of the genotype data and those individuals in the (largest) cluster corresponding to European ancestry were retained for the primary analyses because polygenic risk scores have been shown to have low portability between ancestries [14] motivating ancestry-matched PRS studies until cross-ancestry PRS methods are developed, which our main results correspond to (see section *Samples with population stratification* below, which describes analyses that we also performed on individuals of all ancestries in the UK Biobank). A greedy algorithm [20] was then used to remove related individuals, taking advantage of pre-computed pairwise kinship coefficients provided by the UK Biobank. The algorithm first ranks individuals by their number of related pairs (kinship coefficient > 0.044), then removes the individual with the highest number of related pairs within the data, which should eliminate a high degree of relatedness while retaining a high sample size. When pre-computed kinship coefficients are unavailable, plink2 –king-cutoff can be used to achieve relatedness removal similarly. In our simulations that investigate the effect of related individuals in the GWAS and target data, we instead randomly retain one first degree relative (defined as kinship coefficient ≥ 0.177 and ≤ 0.354) of a randomly sampled individual in the GWAS data. Altogether, we retain 557,369 SNPs, 387,392 individuals and 23,429 of their first-degree relatives for the set of analyses performed. For the simulations of population stratified samples, we extracted samples 10 standard deviations from the centroid of the European cluster and defined these as “non-European” samples. Quality control procedures were repeated using the parameters described above after combining these non-European samples with the European

samples, resulting in 387,365 samples of European ancestry and 21,779 individuals of non-European ancestry. Code used to perform the QC and corresponding documentation are available online [21]. This research has been conducted using the UK Biobank Resource under application 18177 (Dr O'Reilly).

Phenotype simulation

Quantitative Traits without population structure

Quantitative phenotypes (Y) with heritability (h^2) of 0, 0.1, and 0.5 were simulated using the UK Biobank genotype data (post QC; see above) as input. Quantitative traits were simulated as:

$$Y = (\alpha + X\beta + \varepsilon)\delta \quad (12)$$

where X is the standardized genotype matrix corresponding to all samples and the β vector corresponds to the effect size associated with each SNP, with either 10k or 100k SNPs randomly selected to be causal with effect size $\beta \sim N(0,1)$, $\beta = 0$ otherwise. $X\beta$ was adjusted such that it has mean 0 and variance h^2; and ε represents the random error, which follows $\varepsilon \sim N\left(0, \sqrt{1-h^2}\right)$. To ensure that EraSOR can be applied to traits that do not only follow a strict standard normal distribution (N~(0,1)) we included α as an intercept parameter ($\alpha \sim N(0,1)$) and δ as a scaling parameter ($\delta \sim U(1,100)$). This was intended only as a basic test of robustness to deviations from a standard normal distribution and we did not consider any more complex non-Gaussian trait distributions. Consequently, an assumption of EraSOR is that it is applied to continuous data that are either normally distributed or are normalised, using e.g. a log transformation or inverse normal transformation, as is standard in linear regression on continuous outcomes.

To model polygenic risk score analyses with sample overlap, we randomly selected either 120k or 250k individuals from the sample of 387,392 individuals available to us (see above) to generate two different sizes of base GWAS data. Next, we randomly sampled 1,000, 5,000, 10,000 or 50,000 individuals from the remaining sample to act as three different sizes of target data, of which 0%, 5%, 10%, 50% or 100% were randomly selected from the base data sample so that there was a known degree of sample overlap between the base and target data. In addition, we generated an "overlap-free" base cohort in which the overlapping samples were removed from the base cohort so that we could compare the result of applying EraSOR against results of physically removing overlapped samples from the base cohort.

In order to search a feasible parameter space in sufficient depth, we only simulate phenotype with heritability of 0.5, with a base cohort of 250k and target cohort of 5,000; only simulate base cohort with 120k samples when the phenotypic heritability is ≤ 0.1 and target cohort has 5,000 samples; and only simulate target cohort with 1,000 and 10k samples when the base cohort contain 250k samples and the phenotypic heritability is ≤ 0.1. The entire set of simulations were repeated 100 times.

Binary Trait

Binary traits were simulated under the liability threshold model [22], simulating a normally distributed liability using Eq. 12 with $\alpha = 0$, $\delta = 1$, and cases defined as samples with disease liability higher than liability thresholds of 0.9, 0.7 and 0.5, corresponding to population prevalences of 0.1, 0.3 and 0.5, respectively. To limit the complexity of our simulations, the sample prevalence of our cohorts follows the population prevalence and we only simulated 10k causal variants.

In the binary trait setting, overlap can be ascertained such that the overlap is among cases, or among controls, or among both. To investigate the effect of case-only or control-only overlap, we randomly selected 120k effective samples (effective samples defined as $N_{eff} = 4/(1/N_{cases} + 1/N_{controls})$ [23]) as the base cohort, and then randomly selected 5,000 effective samples as the target cohort, where 0%, 5%, 10%, 30% or 50% of the cases or of the controls in the target cohort were sampled from the base cohort. We also performed simulations where the overlapping samples were selected at random among cases and controls. An "overlap-free" base cohort was generated with all overlapping samples removed.

In order to search a feasible parameter space in sufficient depth, we only vary the trait heritability when the population prevalence is 0.1, and only vary the population prevalence when the trait heritability is ≤ 0.1. These simulations were repeated 100 times.

Related samples

Spurious inflation in PRS analysis test statistics may also be observed when there are closely related individuals between the base and target cohorts. To investigate the effects of relatedness on PRS results, we repeated the quantitative trait simulations with a modified Eq. 12:

$$Y = (\alpha + X\beta + \theta + \varepsilon)\delta \quad (13)$$

where θ is the shared environment between the related individuals and follows a random normal distribution with mean 0 and variance $\sigma_\theta^2 \in (0, 0.3, 0.6)$ if and only if $\sigma_\theta^2 + h^2 < 1$, with each related pair of individuals having the same θ value. Only simulated 10k causal variants, and with ε represents a combination of non-shared environment and random error, which follows $\varepsilon \sim N\left(0, \sqrt{1 - h^2 - \sigma_\theta^2}\right)$. To model the inter-cohort relatedness, we first select all individuals with a first-degree relative in the UK Biobank (kinship coefficient ≥ 0.177 and ≤ 0.354), of which there are 23,429 individuals, and then randomly select additional samples who do not have any first-degree relatives to form a base cohort containing 250k samples. We then generate target cohorts containing 5,000 samples, with either 0%, 30%, 60% or 100% of the target samples being first-degree relatives of samples in the base cohort. We also generated a reference cohort from the base cohort where all the related samples in the target cohort were replaced by unrelated individuals for benchmarking the performance of EraSOR. The entire set of simulations were repeated 100 times.

Samples with population stratification

An assumption of the EraSOR algorithm is that the environmental stratification (σ_S^2) is zero. When environmental stratification is present, $\frac{N_c^2 F_{ST} \sigma_S^2}{\sqrt{N_1 N_2}}$ from Eq. 10 is no longer 0 and a bias proportional to the environmental stratification and the genetic stratification (F_{ST}) may be introduced. We partition the UK Biobank into European and non-European ancestries and simulated environmental stratifications to test the sensitivity of EraSOR to deviations of each from 0.

UK Biobank samples were divided into European and non-European ancestries based on 4-mean clustering on PC1 and PC2 (see above). Quantitative traits with environmental stratification were then simulated as:

$$Y = (\alpha + X\beta + S + \epsilon)\delta \quad (14)$$

with the environmental stratification term (S) defined as

$$S = \begin{cases} -\frac{\sigma_S}{2}, & \text{Non} - \text{European Ancestry} \\ \frac{\sigma_S}{2}, & \text{European Ancestry} \end{cases}$$

where σ_S^2 can take a value of 0, 0.3 or 0.9 if and only if $\sigma_S^2 + h^2 < 1$, and ϵ represents the residual term, which follows $\varepsilon \sim N\left(0, \sqrt{var(X\beta + S - 2cov(X\beta, S))\ \frac{1-h^2-\sigma_S^2}{h^2+\sigma_S^2}}\right)$, with $\mathrm{cov}(\mathrm{X}\beta, \mathrm{S})$ being the covariance between Xβ and S. To investigate the effect of sample overlap in the presence of environmental and genetic stratification, we randomly selected either 120k or 250k individuals from the sample of 409,144 individuals available to us (see above) to generate two different sizes of base GWAS data. Next, we randomly sampled 5,000 or 10,000 individuals from the remaining sample to act as two different sizes of target data, of which 0%, 10%, 50% or 100% were randomly selected from the base data sample. To ensure that the genetic and environmental stratification is the same within the base and target data, the same ancestry ratio was maintained in all simulated data sets, matching the ratio in the full data set (~5% non-European ancestry). In addition, we generated an “overlap-free” base cohort in which the overlapping samples were remove from the base cohort to allow benchmarking the performance of EraSOR. The entire set of simulations were repeated 50 times.

Real UK Biobank phenotype analysis

To investigate the effect of sample overlap in real phenotypic data, we extract Body Mass Index (BMI, field ID 21001), Height (field ID 50) and Low-Density Lipoprotein (LDL, field ID 30780) from the UK Biobank. We residualise the phenotypes against age (field ID 21003), sex (field ID 31), genotyping batch, UK Biobank assessment centre (field ID 54) and 40 Principal Components (PCs). For LDL, we additionally removed individuals who were on statin medication (see Supplementary Materials), and additionally included fasting time (field ID: 74) and dilution factor (field ID: 30897) as covariates. The residuals were standardised and used as a phenotype for downstream analysis.

To model polygenic risk score analyses with sample overlap, we randomly sampled 2/3 of the individual with phenotypic information to generate the base GWAS data. Next, we randomly sampled 5,000, 10,000, 5,000 and 1/3 of the individuals with phenotypic information from the remaining sample to act as the target data, of which 0%, 5%, 10%, 50%, 60%, 70%, 80%, 90% or 100% were randomly selected from the base data sample so that there was a known degree of sample overlap between the base and target data. In addition, we generated an “overlap-free” base cohort in which the overlapping samples were removed from the base cohort so that we could compare the results of applying EraSOR with the results of direct removal of overlapping samples from the base cohort. The entire set of simulations was repeated 50 times.

In addition, we utilize the latest GWAS summary statistics on LDL from the Global Lipids Genetics Consortium (GLGC) [24] to evaluate the performance of EraSOR. The GLGC has made available summary statistics with or without the inclusion of UK Biobank samples, as well as ancestry-specific and trans-ancestry summary statistics. We generated polygenic risk scores (PRS) using the GLGC summary statistics with UK Biobank samples excluded, representing the "overlap-free" cohort, to calculate the expected performance of PRS. We then compared the performance of the EraSOR-adjusted and unadjusted PRS calculated using the UK Biobank-included GLGC summary statistics against the expected performance.

Genome-Wide Association Study and Polygenic Score Analysis

Genome-wide association analyses (GWAS) were performed on the base and target cohorts using PLINK 2.0 (version 2021-08-04) [25] with the *--glm* function. As binary traits were only simulated for the European ancestry only analyses, where population structure was not simulated, and considering the computational cost of including covariates in the logistic regression, we did not include PCs in our binary trait analysis. On the other hand, quantitative traits were simulated in all scenarios, some of which are population stratified. Thus, we included 15 PCs as a covariate for our quantitative trait analyses. The resulting summary statistics were then provided to EraSOR to generate the adjusted summary statistics using European LD scores [18] calculated from 1,000 Genomes Project Phase 3 data [26]. PRS analyses using the adjusted, unadjusted, and the "overlap-free" summary statistics were performed using PRSice-2 (v2.3.5) [27] with the default settings. The R^2 and *P*-value of association of the PRS-trait tests were reported.

Strategy for Benchmarking

To investigate the level of spurious inflation caused by inter-cohort relatedness and overlapped samples, we first established a baseline PRS R^2, calculated using base cohorts without overlapped samples. The bias can then be measured as the observed PRS R^2 minuses the baseline PRS R^2 (ΔR^2), given the same phenotype and cohort sizes. For non-heritable traits, we also measure the level of false-positive, defined as any PRS with *P*-value < 1×10^{-4} [28].

On the other hand, to compare the performance of EraSOR with the optimal strategy of directly removing overlapping samples – an option that is typically not available – we calculate PRSs: (i) using summary statistics adjusted by EraSOR ("adjusted PRS") and (ii) using summary statistics generated from a base cohort with all overlapping and/or related samples removed ("overlap-free PRS"). We present the performance of EraSOR as the PRS-trait association R^2 of the adjusted PRS minuses the R^2 of the overlap-free PRS (ΔR^2). If EraSOR has successfully corrected for the sample overlap, then ΔR^2 should be close to 0.

Results

Inflation caused by overlap

The presence of overlapping samples between the base and target data sets is known to cause inflated association between polygenic risk scores (PRS) and phenotypes, but the extent and characteristics of the problem have not been described. Here, we performed extensive simulations using the UK Biobank [8] genotype data to investigate the inflation caused by different levels and types of inter-cohort sample overlap in relation to traits simulated with varying heritability and prevalence (see Methods). Base and target cohorts were generated with varying degrees of sample overlap, measured as $\frac{N_c}{\sqrt{N_1 N_2}}$, where N_c is the number of overlapping samples and N_1 and N_2 are the sample sizes of the base and target cohort, respectively. PRS analyses were conducted using the standard *clumping+thresholding* (C+T) PRS calculation method [1], implemented in *PRSice* [27,28].

We first estimated the false-positive rate induced by sample overlap by simulating non-heritable traits and recording the fraction of significant PRS-trait association (Supplementary Fig. 1). Highly significant associations between PRS and non-heritable phenotypes were observed when even limited inter-cohort sample overlap was present (Fig. 1). Specifically, for non-heritable quantitative traits, the inflation in association (e.g., *p*-value of association) is highly positively correlated with the degree of overlap (Pearson Correlation coefficient (γ) = 0.96, *P*-value < 2.2×10^{-16}). For example, when there is a base cohort of 250k

samples, target cohort of 5,000 samples and 250 overlapping samples (5% of target sample; degree of overlap = 0.0071) the false positive rate is 16%, while this increases to 90% when there are 500 overlapping samples (10% of target sample; degree of overlap = 0.014) (Fig 1a).

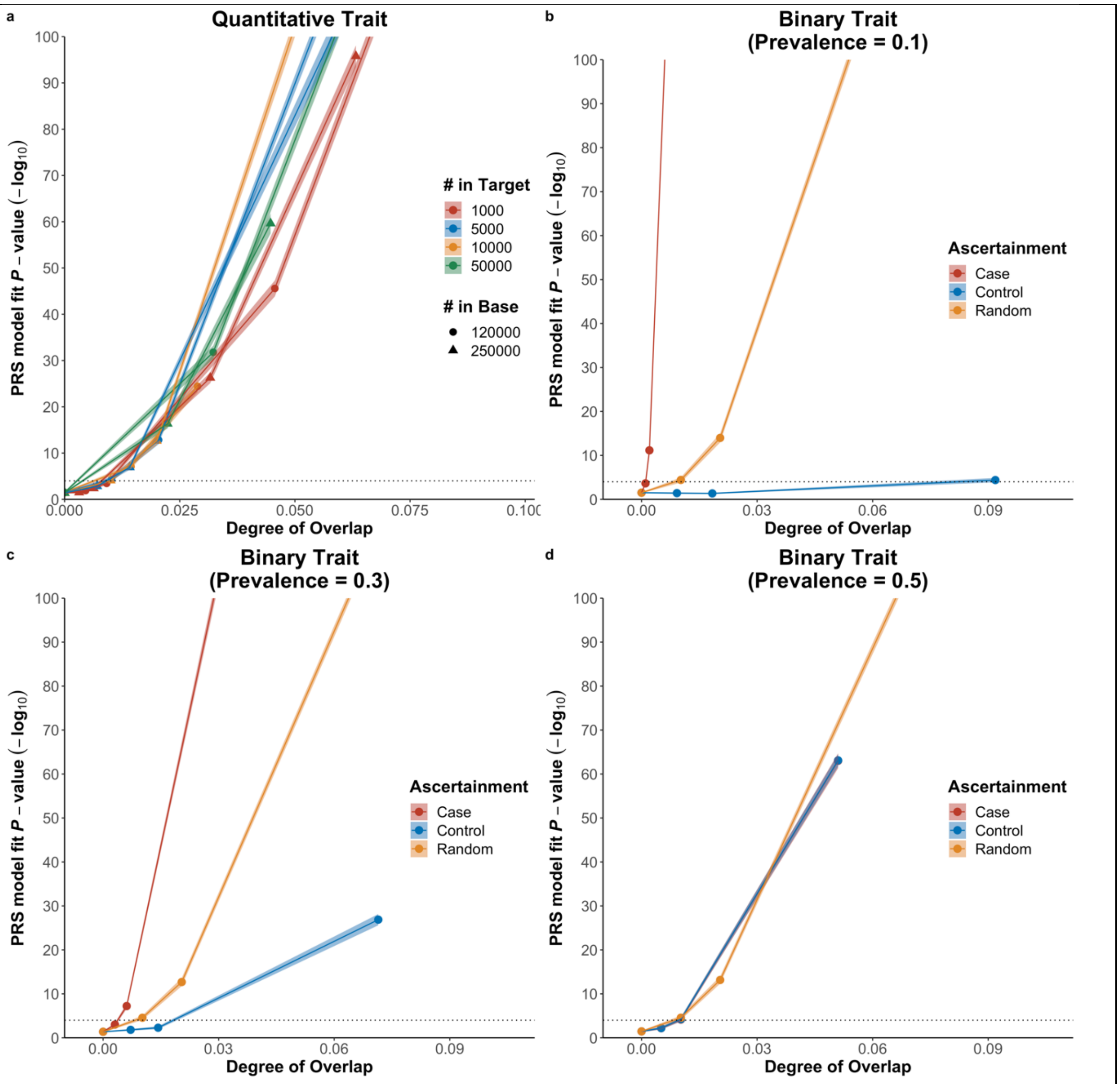

Figure 1. Effect of sample overlap on performance of PRS for non-heritable traits. The dotted line represents the significance threshold (*P*-value = 1x10^{-4}) for high-resolution testing in *PRSice-2* [27]. The X-axis shows the degree of overlap, calculated as $\frac{N_c}{\sqrt{N_1 N_2}}$ and the Y-axis shows the -log$_{10}$ transformed p-value of association between the PRS and the simulated phenotype. Shaded area represents the 95% confidence interval. However, as the confidence interval is small, it is difficult to observe on the scale of these plots **a)** Quantitative traits with different cohort sizes. **b)** Binary traits with population prevalence of 0.1 **c)** Binary traits with population prevalence of 0.3 **d)** Binary traits with population prevalence of 0.5

In the binary trait setting, sample overlap may be among cases only, controls only, or be among both. These alternatives were investigated by first simulating binary traits with different population prevalence using

the liability threshold model [22]. Cohorts with effective sample sizes of 120k in the base data and 5000 in the target data were generated with different degrees and scenarios of sample overlap. We observed extreme inflation associated with case-only overlap when population prevalence is lower than 0.5. For a binary trait with population prevalence 0.1, a false positive rate of 40% is observed when the degree of overlap is 0.001, which corresponds to 5% of the cases from the target cohort also present in the base cohort (Fig 1b). When the degree of sample overlap is doubled (~0.002), the false positive rate is 100%. The inflation in PRS-trait association is not as sensitive to control-only sample overlap when the population prevalence is small. We observe a false positive rate of 50% when the degree of overlap is as high as 0.092, which corresponds to 50% of the controls from the target cohort also present in the base cohort (Fig 1b). This discrepancy between the effect of case and control overlap is a result of the differential contribution of cases and controls to the PRS-trait association in our simulations. Cases are sampled from the extreme upper tail of the liability distribution at a frequency corresponding to the disease prevalence, which is typically low: this gives each case greater weight in the calculation of the PRS-trait association and, thus, an overlapping case will generate greater inflation than an overlapping control. This was consistent with our simulation results (Fig 1c, 1d), where the inflation in ΔR^2 caused by overlapping cases decreases as population prevalence increases ($\gamma = -0.245$, P-value = 1.18x10^{-9}). The reverse relationship between inflation and population prevalence was observed for control-only overlap ($\gamma = 0.359$, p-value = 1.18x10^{-19}). For a population prevalence of 0.5, case-only and control-only overlap have the same impact on the inflation (Fig 1d).

Given that complete overlap of individuals in the base and target data can generate PRS-trait associations that are severely inflated, closely related individuals independently enrolled into the base and target cohorts may induce some inflation considering their shared genetics and environment. Here we tested the effect of relatedness between the base and target cohorts on PRS-trait associations in non-heritable traits in a similar way to that for sample overlap (see Methods), where inter-cohort relatedness is defined as $\frac{N_r}{\sqrt{N_1 N_2}}$ where N_r is the number of samples in the target cohort that are first degree relatives with samples in the base cohort. A false positive rate of 100% is observed when the inter-cohort relatedness is 0.042 (250k base, 5k target, 30% of target samples have 1st degree relatives in the base), when the shared environment explains $\geq$30% of the trait variance. See Supplementary Fig. 2 for full results regarding the effects of inter-cohort relatedness on PRS-trait association inflation.

The analyses in this section were performed only to highlight the potential impact of sample overlap, since highly significant PRS-trait associations are observed with overlap even for non-heritable traits for which there should be no PRS-trait association. EraSOR should not be applied to data on traits for which there is little evidence of heritability, since PRS analyses performed on underpowered GWAS are more likely to generate misleading results and conclusions based on them. Therefore, as for PRS analyses in general [1], we do not recommend the application of EraSOR to base GWAS data with estimated h^2_{SNP} < 5%.

In the next section we extend these investigations to consider the effects of sample overlap on PRS-trait associations on heritable traits, but we present these findings in conjunction with results based on the application of our method EraSOR, which is designed to resolve the problem.

Performance of EraSOR

To tackle the problem of inflation caused by inter-cohort overlap and relatedness, we developed the Erase Sample Overlap and Relatedness (EraSOR) method. Using GWAS summary statistics generated from the base and target cohorts, EraSOR implements univariate and bivariate LD score regression [18,19] to estimate several parameters that are then used to perform a de-correlation calculation of the base GWAS test statistics (see Methods). These adjusted base GWAS summary statistics can then be used for downstream PRS analyses, with sample overlap or relatedness corrected for.

In order to evaluate the performance of EraSOR, we conducted an extensive set of simulations covering a range of scenarios of inter-cohort sample overlap and relatedness (see below and Methods).

Simulations using UK Biobank data

We observed that for both quantitative and binary traits, EraSOR almost entirely eliminates the inflation caused by inter-cohort overlap and relatedness in our simulations based on UK Biobank (European ancestry base and target samples) data (Figure 2). These simulations modelled a range of scenarios that varied trait heritability, prevalence, degree of overlap and combinations of overlap among cases and controls. For example, simulating quantitative traits with heritability 0.1, a base cohort of 250k samples, target cohort of 5000 samples, and degree of overlap 0.141 – in which all samples in the target data are also in the base GWAS – the mean $\boldsymbol{\Delta R^2}$ is 1.68x10^{-5} (standard error: 3.83x10^{-4}) when there are 10k causal variants and 2.21 x10^{-4} (standard error: 4.77x10^{-4}) when there are 100k causal variants. Left unadjusted, the mean $\boldsymbol{\Delta R^2}$ is approximately 0.35 (standard error 0.00143) and 0.36 (standard error: 0.00141), respectively, suggesting that EraSOR has removed the inflation introduced by sample overlap. A similar pattern of complete removal of the effects of sample overlap is observed for the quantitative traits across the full range of heritability and cohort sample sizes tested (Figure 2a), with the exception that when the target cohort is of similar size as the base cohort and when majority of the target samples were found in the base GWAS, EraSOR adjusted PRS results can deviate from the truth. For example, simulating quantitative traits with heritability 0.1, a base cohort size of 120k, target cohort of 50k samples, and when all samples in the target are found in the base GWAS, the mean $\boldsymbol{\Delta R^2}$ for the adjusted PRS can be as high as 0.0134 (standard error = 3.73x10^{-4}), which is still far closer to the truth than the unadjusted PRS (mean $\boldsymbol{\Delta R^2}$ of 0.537). We argue that in such scenarios, while EraSOR cannot completely remove the biases, it can still be used as a tool for sensitivity analyses, where a large discrepancy in performance between the adjusted and unadjusted PRS suggests there might be a large degree of overlap. Similar performances were also observed when lassosum (v0.4.5) [29] instead of PRSice-2 were used for PRS calculation (see Supplementary Fig. 3)

EraSOR also performs extremely well for binary traits in our simulations (Figure 2b, 2c, 2d). In binary traits with heritability 0.1, population prevalence 0.1, a base cohort of with 120k effective samples, a target cohort of 5,000 effective samples, and with 50% of the target data presented in the base GWAS, the mean ΔR^2 for the adjusted PRS in relation to case-only overlap is 7.52x10^{-5} (standard error = 2.76x10^{-4}) (Fig 2b) and the mean ΔR^2 for the adjusted PRS in relation to control-only overlap is 3.36x10^{-4} (standard error = 2.84x10^{-4}) (Fig 2c). On the other hand, when unadjusted, the mean ΔR^2 in relation to case only overlap is as high as 0.161 (standard error = 0.00110), whereas the mean ΔR^2 is 5.80x10^{-3} (standard error = 3.48x10^{-4}) in relation to control-only overlap.

While EraSOR effectively eliminates inflation caused by inter-cohort overlap in all simulation scenarios tested in relation to heritable traits, false-positive results are still observed after EraSOR adjustment in non-heritable traits when there is a large degree of overlap (> 0.289). For non-heritable quantitative traits with base cohorts of 120k samples and target cohorts of 10k samples, if all target samples are also present in the base cohort, then we observe a false-positive rate of 20%, with a mean ΔR^2 of 5.84x10^{-4} (standard error = 1.03x10^{-4}). This is likely caused by the fact that a key component of the mathematics underlying the EraSOR algorithm (described by Eq. 11 in Methods) includes an estimate of h^2 in its denominator. Therefore, when the trait is non-heritable, Eq 9 may be unstable and lead to an error in the EraSOR adjustment. However, we recommend that polygenic risk score analyses should not be performed on traits with estimated $h^2 <$ 0.05 (see [1]) and, thus, in sufficiently powered applications of PRS, EraSOR should have strong performance.

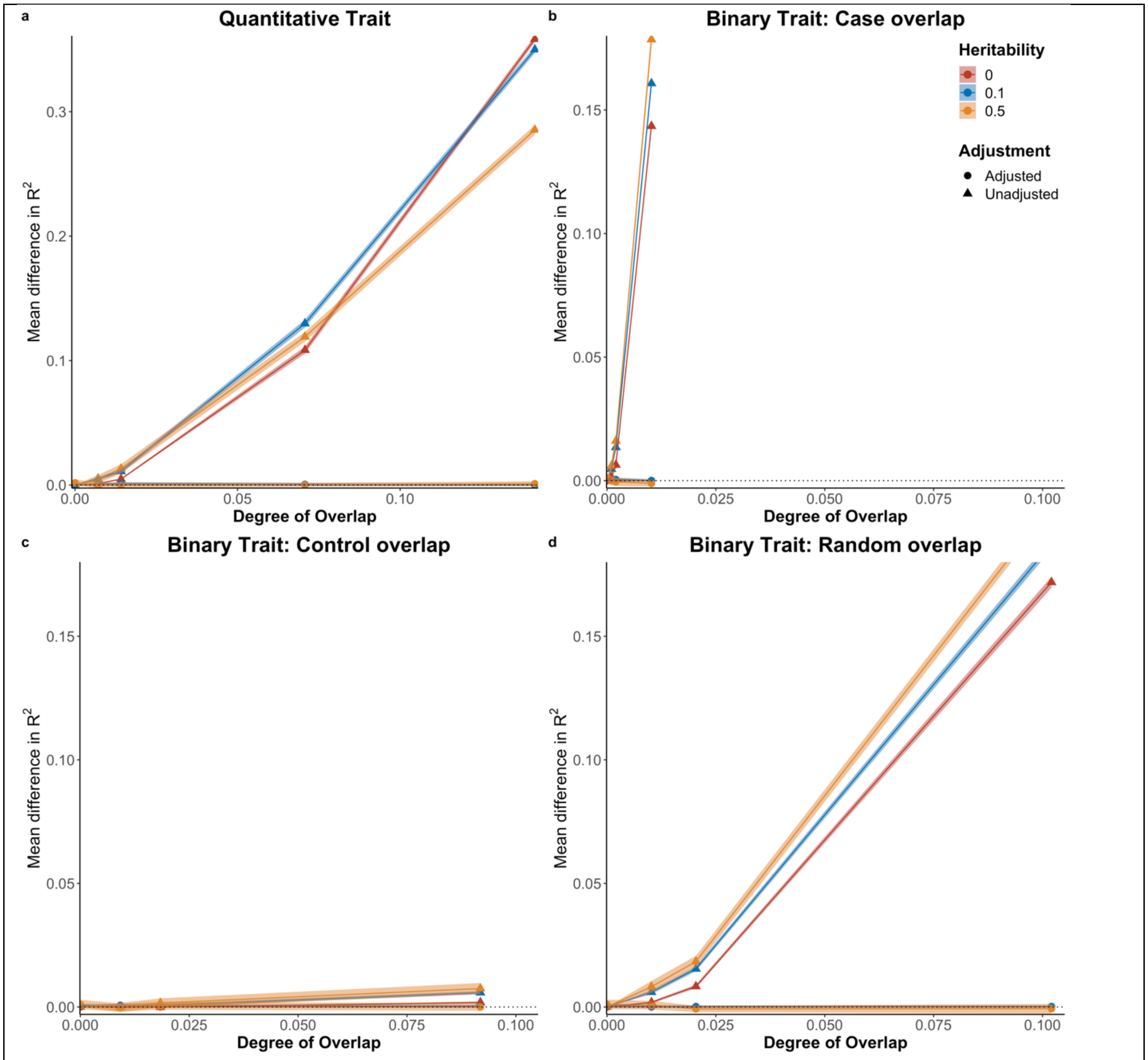

Figure 2. Comparing the performance of the PRS using the EraSOR adjusted summary statistics and the unadjusted summary statistics. The X-axis shows the degree of overlap, and the Y-axis shows the mean difference between the observed R^2 and the expected R^2. Shaded area represents the 95% confidence interval (small on this scale). **a)** Performance in quantitative traits with 250,000 samples in the base cohort and 5,000 samples in the target cohort; b) Performance in binary traits with prevalence of 0.1 and where overlap samples were ascertained for cases; **c)** ascertained for controls; **d)** or were randomly ascertained.

One of the main assumptions of EraSOR is that there is no environmental stratification ($\sigma_S^2 = 0$). To investigate the robustness of EraSOR to model misspecification, we also performed simulations by incorporating UK Biobank samples with non-European ancestry and simulated different level of environmental stratification.

Overall, EraSOR appears to be reasonably robust to model misspecification. For example, considering the high F_{ST} between the European and non-European samples (see Methods) in UK Biobank (F_{ST} = 0.018), coupled with a high environmental stratification (e.g. σ_S^2 =0.3), the mean ΔR^2 is still 5.58x10^{-6} with standard error of 9.11x10^{-4}, for quantitative traits with heritability of 0.1, a base cohort of 250k samples, target cohort of 5000 samples, and a degree of overlap of 0.141. EraSOR performs equally well for

quantitative traits with different heritability, different levels of environmental stratifications and cohorts with different sample size and overlap (see Supplementary Fig. 5-7).

Real UK Biobank phenotype analysis

We performed real data validation of our simulations using height, body mass index (BMI) and low-density lipoprotein (LDL) data from the UK Biobank. In all scenarios, EraSOR completely removed the effects of sample overlap, albeit with slight over-correction in Height and BMI (Figure 3 and Supplementary Table 2).

To further evaluate the performance of EraSOR in real data settings, we used the latest GLGC LDL GWAS summary statistics and the LDL data from the UK Biobank. Using the European-specific GLGC summary statistics, where the UK Biobank samples were removed, the expected PRS-R^2 was 0.102. When the GWAS summary statistics contain the UK Biobank samples, the PRS-R^2 is 0.205, almost doubled the value without sample overlap. However, this inflation was alleviated when we used the EraSOR-adjusted summary statistics, which yielded a PRS-R^2 of 0.0918, represents a relative decrease of 10% compared to the overlap-removed PRS-R^2, consistent with our simulation results.

We note that EraSOR relies on the intercept term from LD score regression, the estimate of which assumes that the level of genetic and environmental stratification is similar between the base and target cohorts [13]. When we used the trans-ancestry GLGC GWAS summary statistics, the PRS-R^2 with the overlapping samples (UK Biobank) removed was 0.145, the PRS-R^2 using the GWAS that included overlapping samples was 0.228, representing a relative increase of 57%. Although we did not observe the same inflation with the EraSOR-adjusted summary statistics, which yielded a PRS-R^2 of 0.0962, its performance was less accurate compared to the case where base and target ancestries were relatively matched, with a relative PRS-R^2 decrease of 33.5%, suggesting that EraSOR might be overly-conservative in such settings.

Overall, our real data analyses confirmed the performance of EraSOR as predicted by our simulations, and we anticipated that EraSOR will be an appropriate tool for sensitivity analyses in real world settings.

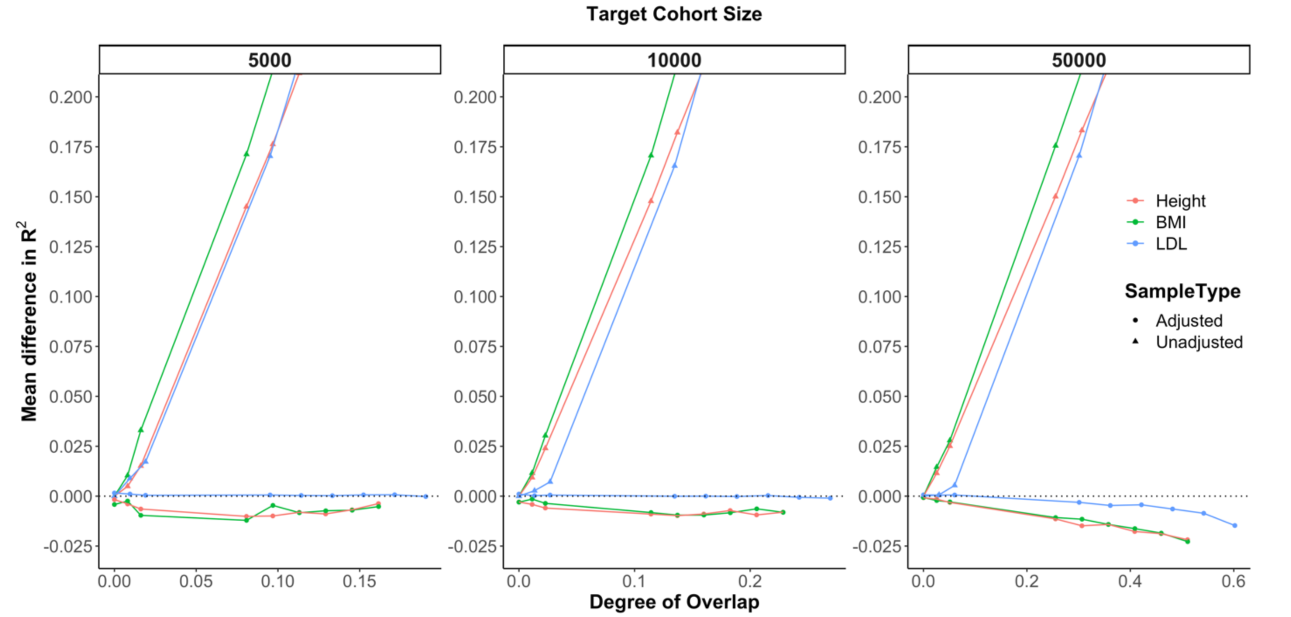

Figure 3. Comparing the performance of PRS using the EraSOR adjusted summary statistics and the unadjusted summary statistics in real UK Biobank phenotypes. The X-axis shows the degree of overlap, and the Y-axis shows the mean difference between the observed R^2 and the expected R^2. Mean difference in $R^2 = 0$ is represented by the black dotted line. Each column corresponds to different target cohort size and different colors correspond to different traits. Performance of the adjusted PRS is indicated with circle and performance of the unadjusted PRS is indicated with triangle. Shaded area represents the 95% confidence interval, which tends to be small.

Discussion

The recent advent of large-scale national and regional biobank projects, such as the UK Biobank [8], Japan Biobank [9] and FinnGen [11], have provided large resources of genotype-phenotype data ideal for conducting polygenic risk score analyses. However, this burgeoning generation of large data has led to an increased risk of inter-cohort sample overlap or relatedness, which can lead to inflated type 1 error. Due to privacy laws and practical concerns, it is usually impossible to identify overlapping samples or related samples across different cohorts. However, ideally researchers would be aware of the scale of the potential problem and have tools to mitigate against it. Therefore, here we reported on an investigation to evaluate the impact of inter-cohort sample overlap and relatedness in PRS analyses and developed a method to account for potential inter-cohort overlap and relatedness that does not require access to raw genotype data from the base GWAS.

We demonstrated that inter-cohort overlap results in a significant and often substantial inflation in the observed PRS-trait association, coefficient of determination (R^2) and false-positive rate. This inflation can be high even when the absolute number of overlapping individuals is small if this makes up a notable fraction of the target samples. The inflation is noticeably more severe for binary traits with a small population prevalence when all the overlapping samples are cases. Therefore, PRS results will likely be misinterpreted unless inter-cohort sample overlap and close relatedness is properly accounted for.

Here, we developed the Erase Sample Overlap and Relatedness (EraSOR) method. EraSOR is designed to correct for inter-cohort sample overlap and relatedness using only summary statistics, without requiring any other information. The results of PRS analyses using EraSOR-adjusted GWAS results in the presence of sample overlap or relatedness was remarkably similar to those gained when the overlap was explicitly removed in most simulated conditions. EraSOR is also robust to misspecification of the model, for example, when there is environmental stratification. While EraSOR does not fully adjust for the bias introduced by inter-cohort overlap for non-heritable traits when the degree of overlap is high, we recommend that researchers should not perform PRS analyses on non-heritable traits in any case [1]. EraSOR performs well for most simulation scenarios tested here, which we believe reflect a large fraction of PRS studies.

Theoretically, as ρ from the bivariate LD score regression is assumed to be the phenotypic correlation [19], we can apply EraSOR in situations where the base and target cohorts measure different phenotypes. Based on LeBlanc's equations [17], the spurious correlations caused by inter-cohort overlap and relatedness is a function of the phenotypic correlation. While this suggests that the impact of inter-cohort overlap and relatedness are likely to be smaller for cross-trait analyses, EraSOR adjustments may still be beneficial in these scenarios. Investigation of the performance of EraSOR in cross-trait analyses should be the subject of future work. Further research is also required to understand the performance and biases of EraSOR for applications in cross-trait studies and in its potential application to GWAS meta-analyses.

Our algorithm is not without limitation. First, as EraSOR relies on LD score intercept estimates for adjustment, all assumptions of LD score regression also apply to EraSOR. For instance, if the level of genetic and environmental stratification differs between the two cohorts, the bivariate LD score equation may not hold [13], which can lead to bias in EraSOR estimates. Another limitation is that EraSOR has only been tested on data from the UK Biobank, which has a maximum genetic stratification corresponding to an F_{ST} of approximately 0.02 (between European and non-European ancestry samples), and, thus, we do not recommend applying EraSOR to data for which the F_{ST} within or between base and target data is greater than 0.02. In practical terms, this means that until further testing or development of EraSOR has been performed, EraSOR should only be applied to single-ancestry base and target data sets that are closely matched by ancestry to each other. As it happens, cohorts of highly different ancestry are less likely to involve sample overlap. Additionally, EraSOR has only been tested using the C+T and lassosum PRS calculation method, and so is subject to their limitations, such as potential overfitting of PRS-trait associations. However, given the similar performance of different PRS methods [29] we do not expect qualitatively different results to those observed here when EraSOR is applied to correct for sample overlap in PRS analyses using other PRS methods.

It is important to note that, due to reliance on LD score regression estimates, EraSOR only produces sufficiently accurate adjustments for application when both base and target cohorts have sample sizes greater than 1,000 and is only consistently accurate when both cohorts have greater than 5,000 samples. In addition, when there is a complete overlap between two equal size cohort, EraSOR can fail to fully adjust for the inflation caused by sample overlap. Despite this, EraSOR adjusted results are still far closer to the empirical truth than the unadjusted PRS results. In general, EraSOR tends to over-correct, meaning that PRS-trait associations are underestimated after EraSOR-adjustment. One possible explanation is that LeBlanc et al.'s equations [17] were based on the null model of no contribution of SNP to the trait. Thus, in cases where SNPs contribute to the phenotype of interest, there will be an upper-bound to the level of inflation caused by sample overlap, and EraSOR might misclassify some of the true signal as inflation caused by sample overlap, leading to over-correction of test statistics.

Nevertheless, we argue that a large discrepancy between the unadjusted and EraSOR-adjusted results should act as a warning as to the possible presence of inter-cohort overlap or close relatedness. In such cases, it is recommended to investigate the potential source of sample overlap or relatedness, and take

appropriate measures to address them, such as removing the related or overlapped individuals, or adjusted for such bias using EraSOR to obtain more reliable PRS analysis results.

Availability of supporting source code and requirements

Project Name	EraSOR
Project Homepage	https://choishingwan.gitlab.io/EraSOR/
Programming Language	Python (version 3.0+)
License	GNU General Public License version 3.0 (GPLv3)
Any restrictions to use by non-academics	None
Simulation Scripts	**https://gitlab.com/choishingwan/sample_overlap_paper**
biotools ID	**erasor**
RRID	SCR_023542

Availability of supporting data and materials

All code used for this paper is available in gitlab repository[30] and were implemented using nextflow (version 20.10.0 build 5430) [31]. Full source code and documentation of EraSOR can be found online [16]. The UK Biobank Resource is retrieved under Application Number 18177 (controlled access). An archival copy of the code and supporting data is available via the GigaScience repository, GigaDB [32].

Abbreviations

PRS: polygenic risk score, GWAS: Genome Wide Association Study

Additional files

Supplementary Table 1: Simulation results

Supplementary Table 2: Results for real data analyses

Supplementary Method and Figures

Competing interests

The authors declare that they have no competing interests.

Funding

Medical Research Council FundRef identification ID: http://dx.doi.org/10.13039/501100000265 MR/N015746/1 and the National Institute of Health (R01MH122866) to P.F.O. This report represents independent research partially funded by the National Institute for Health Research (NIHR) Biomedical Research Centre at South London and Maudsley NHS Foundation Trust and King's College London. Research reported in this paper was supported by the Office of Research Infrastructure of the National Institutes of Health under award number S10OD026880. The content is solely the responsibility of the authors and does not necessarily represent the official views of the National Institutes of Health, NHS, the NIHR or the Department of Health.

Authors' contributions

Conceptualization, S.W.C. and P.F.O.; Methodology, S.W.C., T.S.H.M., C.H. and P.F.O.; Investigation, S.W.C.; Software, S.W.C.; Supervision, P.F.O.; Funding Acquisition, P.F.O.; Writing – Original Draft, S.W.C; Writing - Review and Edition, S.W.C., C.H. and P.F.O.;

Acknowledgements

We thank the participants in the UK Biobank and the scientists involved in the construction of this resource. We thank Jonathan Coleman and Kylie Glanville for helpful discussions. This research has been conducted using the UK Biobank Resource under application 18177 (P.F.O.). This work was supported in part through the computational resources and staff expertise provided by Scientific Computing at the Icahn School of Medicine at Mount Sinai.

Reference

1. Choi SW, Mak TS-H, O'Reilly PF. Tutorial: a guide to performing polygenic risk score analyses. *Nat Protoc*. 2020; doi: 10.1038/s41596-020-0353-1.

2. Mavaddat N, Michailidou K, Dennis J, Lush M, Fachal L, Lee A, et al.. Polygenic Risk Scores for Prediction of Breast Cancer and Breast Cancer Subtypes. *Am J Hum Genet*. 2019; doi: 10.1016/j.ajhg.2018.11.002.

3. Zhang J-P, Robinson D, Yu J, Gallego J, Fleischhacker WW, Kahn RS, et al.. Schizophrenia Polygenic Risk Score as a Predictor of Antipsychotic Efficacy in First-Episode Psychosis. *Am J Psychiatry*. 2019; doi: 10.1176/appi.ajp.2018.17121363.

4. Natarajan P, Young R, Stitziel NO, Padmanabhan S, Baber U, Mehran R, et al.. Polygenic Risk Score Identifies Subgroup with Higher Burden of Atherosclerosis and Greater Relative Benefit from Statin Therapy in the Primary Prevention Setting. *Circulation*. 2017; doi: 10.1161/CIRCULATIONAHA.116.024436.

5. Mega JL, Stitziel NO, Smith JG, Chasman DI, Caulfield MJ, Devlin JJ, et al.. Genetic risk, coronary heart disease events, and the clinical benefit of statin therapy: an analysis of primary and secondary prevention trials. *The Lancet*. Elsevier; 2015; doi: 10.1016/S0140-6736(14)61730-X.

6. Pain O, Hodgson K, Trubetskoy V, Ripke S, Marshe VS, Adams MJ, et al.. Antidepressant Response in Major Depressive Disorder: A Genome-wide Association Study. *medRxiv*. Cold Spring Harbor Laboratory Press; 2020; doi: 10.1101/2020.12.11.20245035.

7. Dudbridge F. Power and Predictive Accuracy of Polygenic Risk Scores. *PLOS Genet*. 2013; doi: 10.1371/journal.pgen.1003348.

8. Sudlow C, Gallacher J, Allen N, Beral V, Burton P, Danesh J, et al.. UK Biobank: An Open Access Resource for Identifying the Causes of a Wide Range of Complex Diseases of Middle and Old Age. *PLOS Med*. 2015; doi: 10.1371/journal.pmed.1001779.

9. Nagai A, Hirata M, Kamatani Y, Muto K, Matsuda K, Kiyohara Y, et al.. Overview of the BioBank Japan Project: Study design and profile. *J Epidemiol*. 2017; doi: 10.1016/j.je.2016.12.005.

10. Fan C-T, Lin J-C, Lee C-H. Taiwan Biobank: a project aiming to aid Taiwan's transition into a biomedical island. *Pharmacogenomics*. 2008; doi: 10.2217/14622416.9.2.235.

11. Kurki MI, Karjalainen J, Palta P, Sipilä TP, Kristiansson K, Donner KM, et al.. FinnGen provides genetic insights from a well-phenotyped isolated population. *Nature*. Nature Publishing Group; 2023; doi: 10.1038/s41586-022-05473-8.

12. Sullivan PF, Agrawal A, Bulik CM, Andreassen OA, Børglum AD, Breen G, et al.. Psychiatric Genomics: An Update and an Agenda. *Am J Psychiatry*. 2018; doi: 10.1176/appi.ajp.2017.17030283.

13. Yengo L, Sidorenko J, Kemper KE, Zheng Z, Wood AR, Weedon MN, et al.. Meta-analysis of genome-wide association studies for height and body mass index in ∼700000 individuals of European ancestry. *Hum Mol Genet*. 2018; doi: 10.1093/hmg/ddy271.

14. Global Lipids Genetics Consortium, Willer CJ, Schmidt EM, Sengupta S, Peloso GM, Gustafsson S, et al.. Discovery and refinement of loci associated with lipid levels. *Nat Genet*. 2013; doi: 10.1038/ng.2797.

15. Wray NR, Yang J, Hayes BJ, Price AL, Goddard ME, Visscher PM. Pitfalls of predicting complex traits from SNPs. *Nat Rev Genet*. 2013; doi: 10.1038/nrg3457.

16. Choi SW: EraSOR GitLab repository. GitLab 2023. https://gitlab.com/choishingwan/EraSOR

17. LeBlanc M, Zuber V, Thompson WK, Andreassen OA, Frigessi A, Andreassen BK, et al.. A correction for sample overlap in genome-wide association studies in a polygenic pleiotropy-informed framework. *BMC Genomics*. 2018; doi: 10.1186/s12864-018-4859-7.

18. Bulik-Sullivan BK, Loh P-R, Finucane HK, Ripke S, Yang J, Schizophrenia Working Group of the Psychiatric Genomics Consortium, et al.. LD Score regression distinguishes confounding from polygenicity in genome-wide association studies. *Nat Genet*. 2015; doi: 10.1038/ng.3211.

19. Bulik-Sullivan B, Finucane HK, Anttila V, Gusev A, Day FR, Loh P-R, et al.. An atlas of genetic correlations across human diseases and traits. *Nat Genet*. 2015; doi: 10.1038/ng.3406.

20. Choi SW: GreedyRelated · GitLab. GitLab. https://gitlab.com/choishingwan/GreedyRelated (2020).

21. Choi SW: Data Preparation - UK Biobank administration. https://choishingwan.gitlab.io/ukb-administration/admin/master_generation/ (2020).

22. Falconer DS. Introduction to quantitative genetics. New York,: Ronald Press Co;

23. Willer CJ, Li Y, Abecasis GR. METAL: fast and efficient meta-analysis of genomewide association scans. *Bioinformatics*. 2010; doi: 10.1093/bioinformatics/btq340.

24. Graham SE, Clarke SL, Wu K-HH, Kanoni S, Zajac GJM, Ramdas S, et al.. The power of genetic diversity in genome-wide association studies of lipids. *Nature*. Nature Publishing Group; 2021; doi: 10.1038/s41586-021-04064-3.

25. Chang CC, Chow CC, Tellier LC, Vattikuti S, Purcell SM, Lee JJ. Second-generation PLINK: rising to the challenge of larger and richer datasets. *GigaScience*. 2015; doi: 10.1186/s13742-015-0047-8.

26. Auton A, Abecasis GR, Altshuler DM, Durbin RM, Abecasis GR, Bentley DR, et al.. A global reference for human genetic variation. *Nature*. Nature Publishing Group; 2015; doi: 10.1038/nature15393.

27. Choi SW, O'Reilly PF. PRSice-2: Polygenic Risk Score software for biobank-scale data. *GigaScience*. 2019; doi: 10.1093/gigascience/giz082.

28. Euesden J, Lewis CM, O'Reilly PF. PRSice: Polygenic Risk Score software. *Bioinformatics*. 2015; doi: 10.1093/bioinformatics/btu848.

29. Mak TSH, Porsch RM, Choi SW, Zhou X, Sham PC. Polygenic scores via penalized regression on summary statistics. *Genet Epidemiol*. 2017; doi: 10.1002/gepi.22050.

30. Choi SW: sample_overlap_paper · GitLab repository. GitLab 2023. https://gitlab.com/choishingwan/sample_overlap_paper.

31. Di Tommaso P, Chatzou M, Floden EW, Barja PP, Palumbo E, Notredame C. Nextflow enables reproducible computational workflows. *Nat Biotechnol*. 2017; doi: 10.1038/nbt.3820.

32. Choi SW, Mak TSH, Hoggart C, O'Reilly P. Supporting data for "EraSOR: a software tool to eliminate inflation caused by sample overlap in polygenic score analyses." GigaScience Database; doi: http://dx.doi.org/10.5524/102393
