# Supplementary material for: EraSOR: a software tool to eliminate inflation caused by sample overlap in polygenic score analyses
Source: Gigascience. 2023 Jun 16;12:giad043. doi: 10.1093/gigascience/giad043 (PMC10273836; doi:10.1093/gigascience/giad043)
Supplement: giad043_Supplemental_File [file giad043_supplemental_file.docx]

# Supplementary Materials


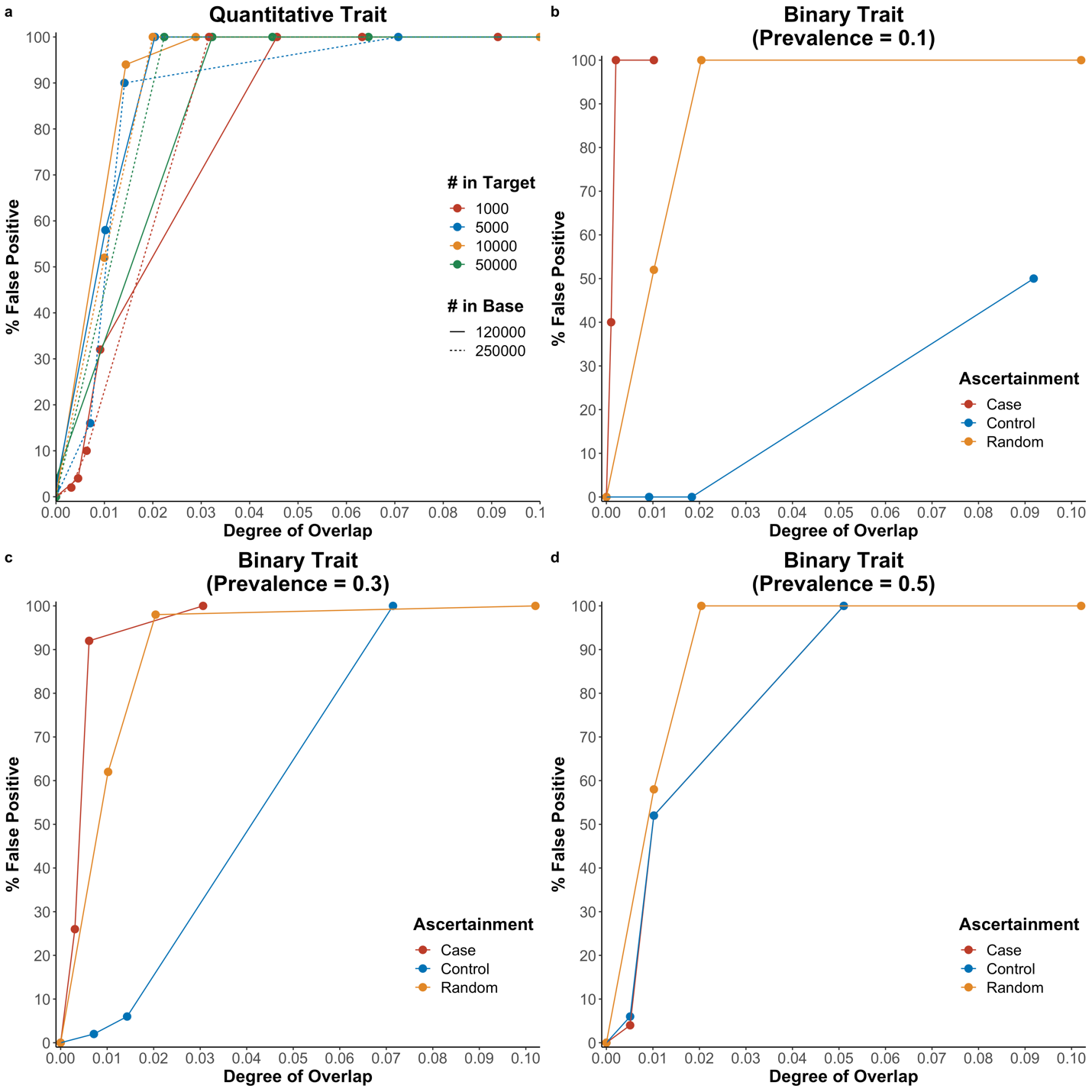


**Supplementary Figure 1.** False positive rate corresponding to different level of sample overlap. Non-heritable phenotypes were simulated. X axis shows the degree of overlap, calculated as $\frac{N_{c}}{\sqrt{N_{1}N_{2}}}$ and the Y-axis shows the percentage of false positive (PRS P-value < 1x10-4). **a)** Quantitative traits with different cohort sizes **b)** Binary traits with population prevalence of 0.1 **c)** Binary traits with population prevalence of 0.3 **d)** Binary traits with population prevalence of 0.5.


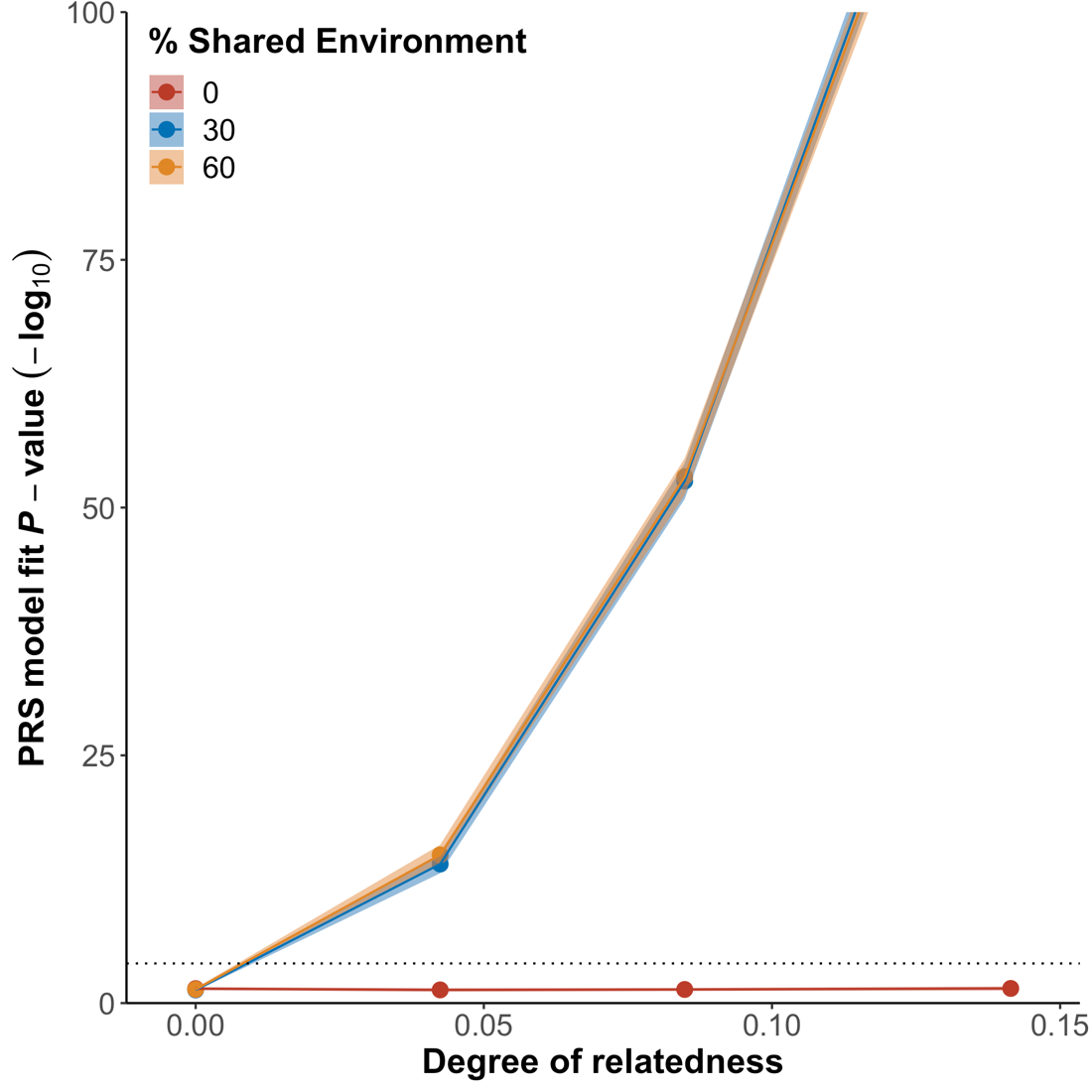


**Supplementary Figure 2.** Effect of sample relatedness on performance of PRS for non-heritability phenotypes. The dotted line represents the significant threshold i.e., p-value < 1x10^-4^. Y-axis represents the -log_10_ transformed *p*-value of association between the PRS and the phenotype; the X-axis represents the degree of relatedness between the target cohort and the base cohort, calculated $\frac{N_{r}}{\sqrt{N_{1}N_{2}}}$ where $N_{r}$ is the number of samples in the target cohort that are first degree relatives to sample in the base cohorts. Shaded area represents the 95% confidence interval.


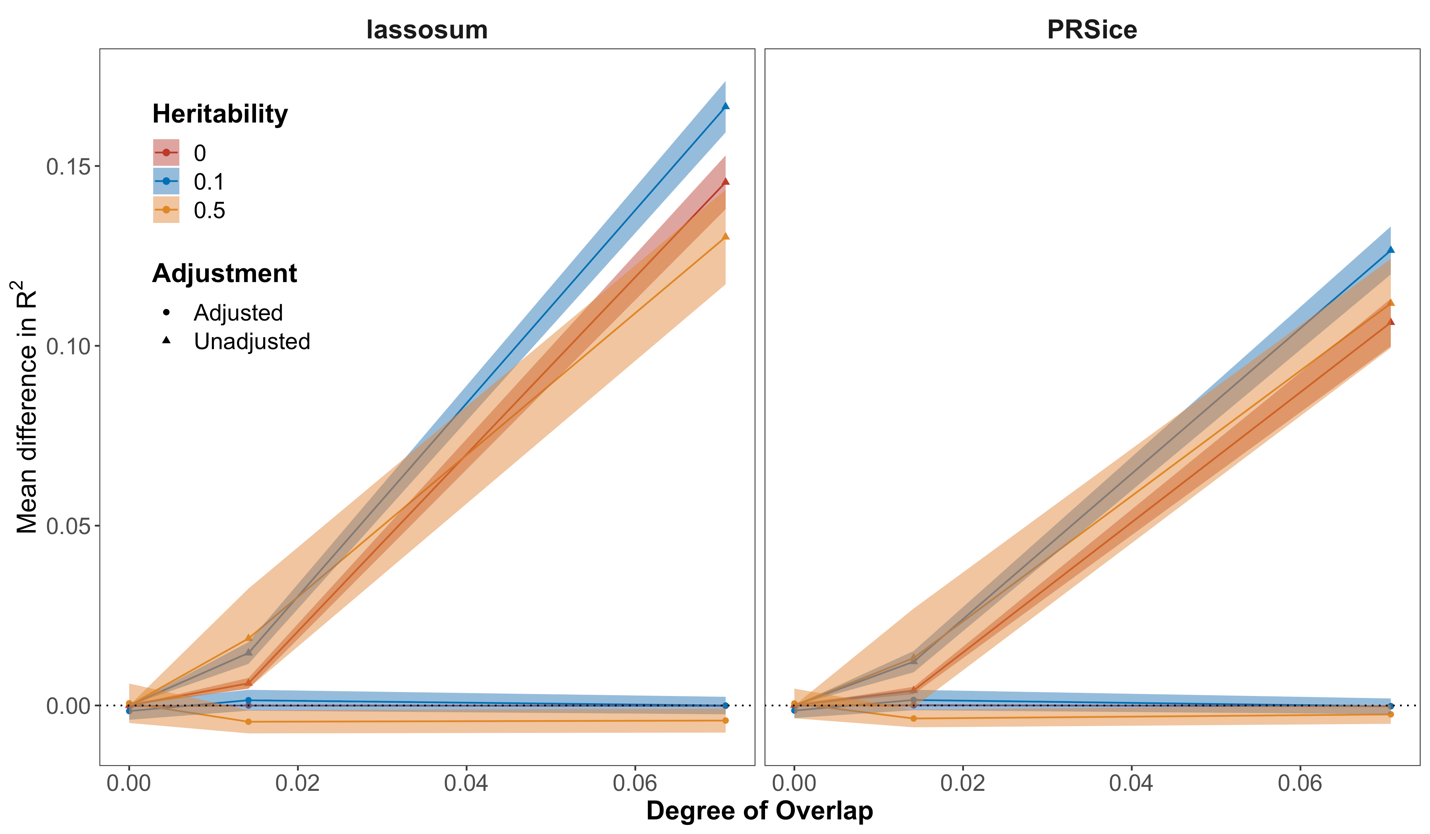


**Supplementary Figure 3** Comparing the performance of the PRS using the EraSOR adjusted summary statistics and unadjusted summary statistics. The X-axis shows the degree of overlap, and the Y-axis shows the mean difference between the observed R^2^ and the expected R^2^. Shaded area represents the 95 confidence interval. 10 permutations were performed on quantitative traits with 250,000 samples in the base cohort and 5,000 samples in the target cohort. a) PRS calculated using lassosum; b) PRS calculated using PRSice-2.


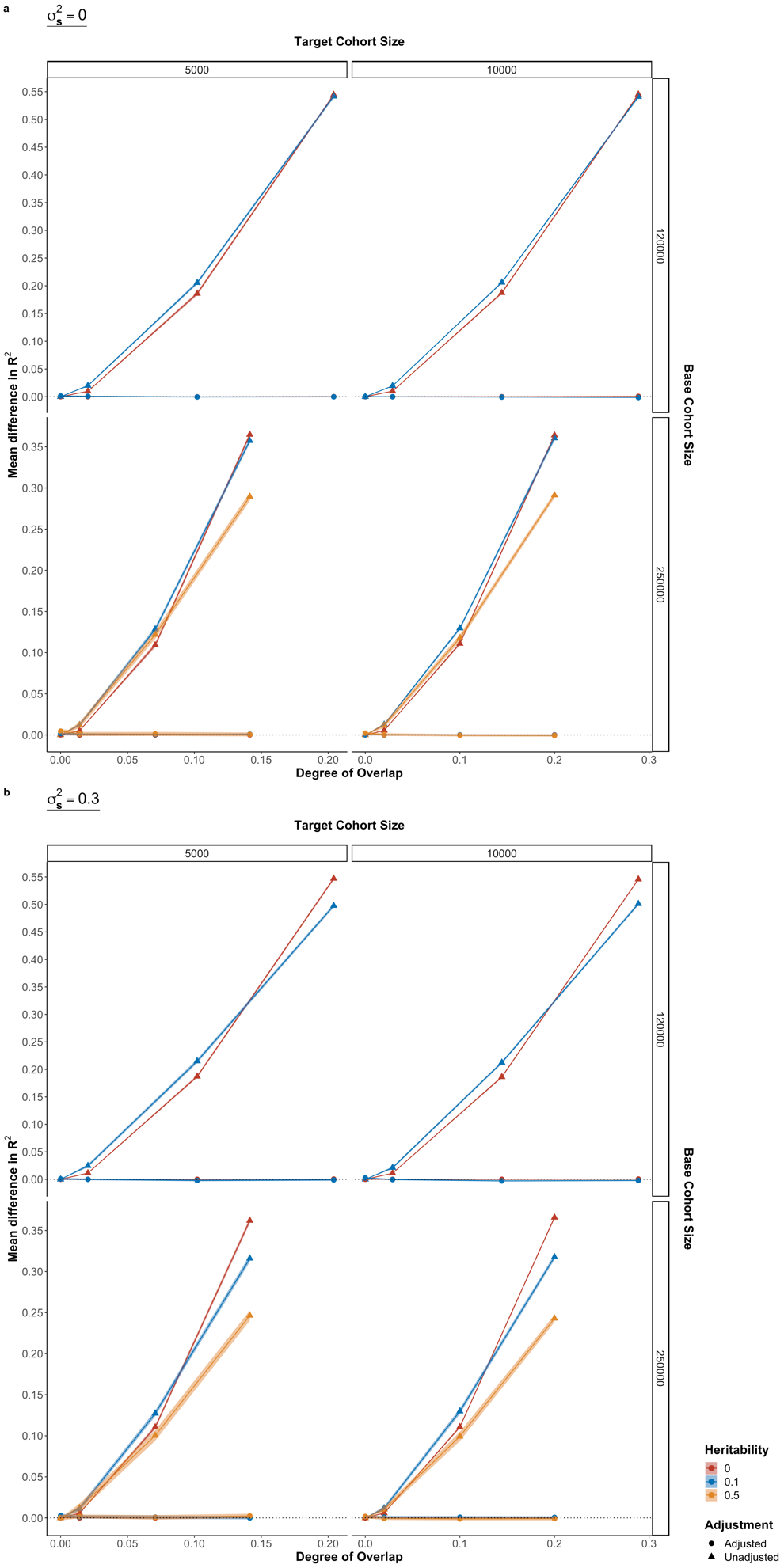


**Supplementary Figure 4.** Comparing the performance of PRS using the EraSOR adjusted summary statistics and the unadjusted summary statistics for quantitative trait when there are population stratifications. Samples from non-European ancestries were included in this analysis. Different level of environmental stratifications was simulated: **a)** no environmental stratification **b)** environmental stratification = 0.3. Shaded area represents the 95% confidence interval, with different colours represent different simulated heritability. Results of the unadjusted PRS were represented with triangle, while results of the adjusted PRS were represented with circle.


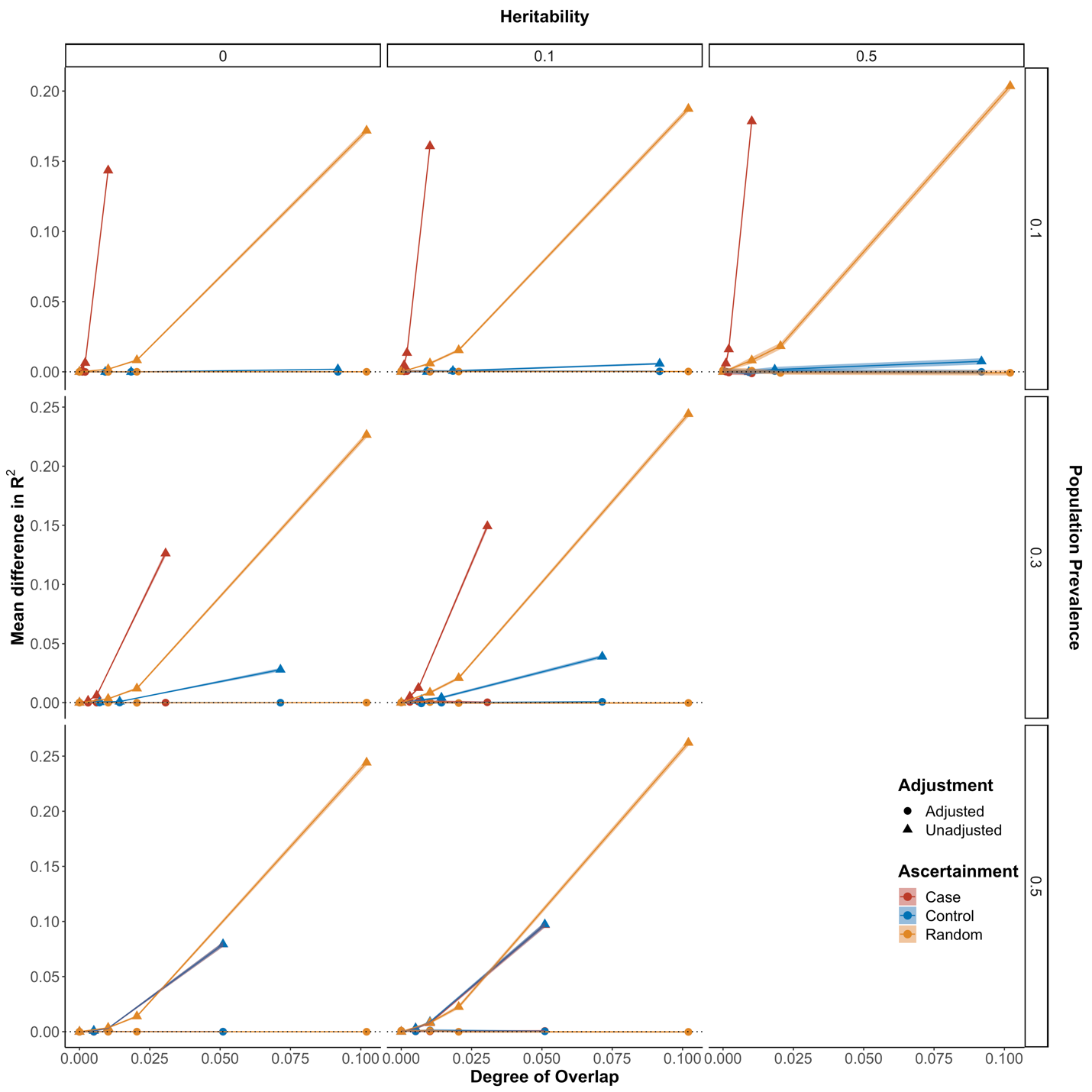


**Supplementary Figure 5.** Comparing the performance of PRS using the EraSOR adjusted summary statistics and the unadjusted summary statistics for binary trait analyses. The X-axis shows the degree of overlap, and the Y-axis shows the mean difference between the observed R^2^ and the expected R^2^. Mean difference in R^2^ = 0 is represented by the black dotted line. Each row corresponds to different trait heritability, each column corresponds to different population prevalence and colors were used to represent different ascertainment of the overlapped samples. Performance of the adjusted PRS is indicated with circle and performance of the unadjusted PRS is indicated with triangle. Shaded area represents the 95% confidence interval, which tends to be small.


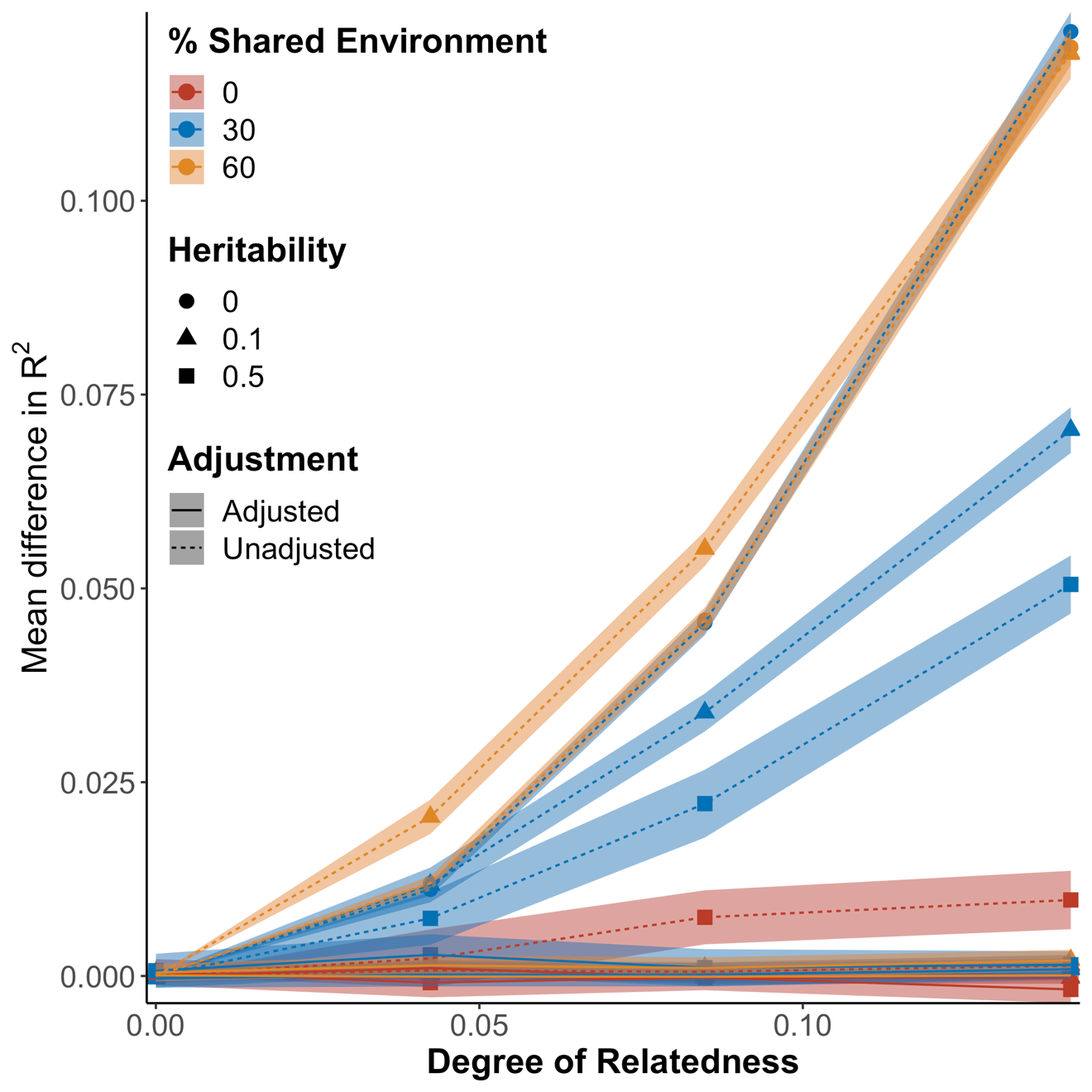


**Supplementary Figure 6**. Performance of EraSOR when adjusting for related samples between the target cohort and the base cohort. The X-axis shows the degree of overlap, and the Y-axis shows the mean difference between the observed R^2^ and the expected R^2^. Shaded areas represent the 95% confidence interval. Different colours correspond to the amount of shared environmental contribution, and the shapes represents the heritability. Performance of EraSOR adjusted PRS are represented with the dotted line, and the performance of the unadjusted PRS are represented with the solid line.

# Supplementary Methods

## Statin Medication used definition

Statin medications were extracted from field ID 20003, with code 1141146234, 1141192414, 1140910632, 1140888594, 1140864592, 1141146138, 1140861970, 1140888648, 1141192410, 1141188146, 1140861958, 1140881748, 1141200040 and 1140861922.

1. Su Z, Marchini J, Donnelly P. HAPGEN2: simulation of multiple disease SNPs. *Bioinforma Oxf Engl*. 2011; doi: 10.1093/bioinformatics/btr341.

2. Auton A, Abecasis GR, Altshuler DM, Durbin RM, Abecasis GR, Bentley DR, et al.. A global reference for human genetic variation. *Nature*. Nature Publishing Group; 2015; doi: 10.1038/nature15393.

3. Abraham G, Qiu Y, Inouye M. FlashPCA2: principal component analysis of Biobank-scale genotype datasets. *Bioinformatics*. 2017; doi: 10.1093/bioinformatics/btx299.
