# Supplementary material for: EraSOR: a software tool to eliminate inflation caused by sample overlap in polygenic score analyses
Source: Gigascience. 2023 Jun 16;12:giad043. doi: 10.1093/gigascience/giad043 (PMC10273836; doi:10.1093/gigascience/giad043)
Supplement: giad043_Response_to_Reviewer_Comments_Original_Submission [file giad043_response_to_reviewer_comments_original_submission.pdf]

Author's Response To Reviewer Comments

Close

Reviewer #1: This paper addresses a significant need that has arisen in the interaction between privacy rules and ever-larger genomic datasets, and I find the results to be very promising and clearly worth publishing. I just have a few comments on some methodological details:

>> We thank the reviewer for their positive comments on the utility and likely impact comment of our paper.

line 130: Have you compared the effectiveness of this algorithm with plink2 --king-cutoff?

>> The greedy related algorithm is different from plink2 --king-cutoff in that it does not calculate the relatedness between individuals but uses existing kinship calculation (pre-computed in the UK Biobank) for sample removal. It first ranks samples according to their number of related pairs based on the given kinship information, then removes the individual with the highest number of related pairs within the data, which should eliminate a high degree of relatedness while retaining a high sample size. This process iterates until there are no remaining closely related individuals. This algorithm is particularly useful for the UK Biobank data as the pairwise relatedness information was already calculated, allowing us to quickly remove related samples without recalculating the kinship information. If the kinship information is unavailable, then plink2 --king-cutoff can be used instead.

>> We have now explained this explicitly in the Methods section and have added the recommendation to use plink2 –king-cutoff if relatedness hasn't been pre-computed as it was in the UK Biobank.

lines 145-155: If I understand this correctly, these simulated quantitative traits are still normally distributed, they just aren't standardized to mean 0 variance 1. If the intent is to "simulate phenotypes that [do] not follow the standard normal distribution", I'd expect it to be more valuable to look at e.g. the log-normal case, where an alert user might transform the phenotype to normal, but some users may fail to do so. A mixture distribution may also be worth looking at.

>> Our intent here was only to perform a basic 'sanity check' that our results were unaffected when applied to a trait that does not strictly follow a standard normal distribution, rather than performing a fuller investigation of the impact of application to non-normally distributed traits that were not normalized by users. We have now made our intention here clear and have clarified that application of EraSOR assumes that non-normally distributed continuous traits have been normalized as standard.

lines 238-239: Have you considered using the "cc-residualize" option of plink2 --glm, which removes most of the computational cost of including PCs in your binary trait analysis?

>> We thank the reviewer for suggesting the `cc-residualize` option, which we were unaware of. We did consider performing a residualization of this kind, but decided not to because we did not simulate population stratification in the case control analyses and, thus, there was no need to control for PCs here.

lines 383-387: This is interesting; there is some room for follow-up investigation here. Thanks for posting all the scripts needed for another researcher to easily reproduce this Fst=0.00639 value; this could help facilitate development of a better genotype-simulation tool.

>> We thank the reviewer for their positive comments on this.

Also, some minor copyedits:
line 84: "subpopulation" -> "subpopulations"

line 342: "overlaps" -> "overlap"
line 363: "ErasOR" -> "EraSOR"
line 376: "different level of environmental stratifications" -> "different levels of environmental stratification"
line 384: "population" -> "populations"
line 402: "capture" -> "captured"

>> We thank the reviewer for pointing out these our typos. All of these typos have now been corrected in the revision.

Reviewer #2: Overall, I think that this manuscript is strong and describes a well-formulated method to address a relevant problem. There are a few outstanding questions about the performance of the EraSOR method from my perspective, which I'll detail as follows.

>> We thank the reviewer for their positive comments on our manuscript, the formulation of our method and the relevance of the problem that our method sets out to tackle.

My understanding of reference [16] indicates that equation (3) of this manuscript only holds for null SNPs, i.e. if SNP g is not associated with the outcome Y. If this is the case, then this should be discussed in the manuscript. I wonder if this can partially explain the 'under-estimation' behavior we see in the application to real data in Supplementary Figure 3. In particular, I am referencing the behavior where the EraSOR correction will under-estimate the predictive accuracy of the PRS in the target data, i.e. where delta-R^2 is negative. This behavior is not seen in the simulation and warrants further investigation and discussion. While the bias appears small, for some cases delta-R^2 approaches -.025, which corresponds to an under-estimation of Pearson's r by roughly .15; this is substantial. Could it be the case that, for highly polygenic traits such as height and BMI, the null-SNP assumption is unreliable and the performance of EraSOR is degraded? Does a fundamental assumption of sparse genetic association underlie EraSOR?

>> We thank the reviewer for highlighting this important point. Eq.2, and thus eq.3, are indeed based on an assumption of null SNPs, which we previously stated following Eq.1 but which we now repeat more explicitly at the end of the 'EraSOR framework' in the Methods section. Based on the reviewer's comments, we have now extended our quantitative simulations to incorporate 100k causal variants (i.e. 10x the fraction of causal variants as we originally simulated). These new simulation results indicate that the polygenicity of the trait is not the cause of the downward bias in the correction. On the other hand, when we avoid the use of EraSOR altogether and instead plug-in the known degree of sample overlap into LeBlanc's equation, then we observe a similar degree of downward bias, suggesting that this may be a result of a limitation in LeBlanc's equation. We have now highlighted this as a limitation in the Discussion.

I recommend that the real data application play a larger role in the manuscript narrative and be moved out of the supplementary. The simulations are appreciated and helpful, but there is nuance in the analysis of real data that cannot be replicated in simulation.

>> We agree with the reviewer that the real data analyses are extremely important in terms of involving relevant nuance that simulations cannot fully model and so we have now moved the entire real data application to the main text.

I believe the reference to "Supplementary Figure 2" on line 346 should actually be "Supplementary Figure 3". I believe that the axis labels in Supp Figure 3 are flipped.

>> Thank you for pointing out these errors. We have now fixed both the reference to the figure and figure axis labels.

Lines 82 and 83 reference genetic stratification and subpopulations; I think the relevance of these concepts should be introduced more clearly and they should be defined in this context. EraSOR concerns the over-estimation of predictive accuracy and association incurred by sample overlap between the base and target GWASs; to this reader, it's not clear what this central issue has to do with population stratification. I realize

that the derivation of the LD score method is motivated heavily by correcting for stratification; however, these concepts should be introduced more clearly in this manuscript.

>> Genetic stratification, and relatedly here sub-populations, is only relevant here in that EraSOR leverages a fully expanded version of the LD score regression equation: one that was formulated by Yengo et al (ref.13) and incorporates two terms that include Fst (which measures genetic stratification). We have now expanded the corresponding section of the Methods to define genetic stratification in this context and to clearly explain its relevance in this setting, which involves inferring sample overlap from an equation that includes an explicit measure (Fst) of genetic stratification.

Line 88: consider defining LD score lj.

>> We have now included the definition of LD score in our updated manuscript.

Lines 94-96: consider outlining the mathematical consequence of the assumption that "the two outcomes and cohorts are identical." It's the case that N_1 = N_2 = N_c = N, correct?

>> That is correct. We have now outlined the mathematical consequence of this assumption, which we agree will help with the reader's understanding here.

Line 109 / equation (11): My understanding is that the relevant quantity of this derivation is N_c / sqrt(N_1 N_2), which allows us to define the correct matrix C in expression (4). If this is the case, perhaps the quantity of interest should be moved to the LHS of the equation in the final line of the expression, for clarity.

>> We thank the reviewer for pointing this out. N_c / sqrt(N_1 N_2) is indeed the quantity of interest. We have now updated equation 11 to have this quantity located on the LHS of the equation for clarity.

As discussed in the manuscript, the estimated heritability is in the denominator of the expression for N_c / sqrt(N_1 N_2). The authors correctly discuss that the method should not be applied when there is doubt as to whether the heritability is different from zero. I would take this a step further; in cases where the heritability is zero, we cannot meaningfully apply the EraSOR correction, and thus I am not sure of the utility of the 'type I error' simulations in the manuscript. Perhaps an explicit test for h^2 > 0 should be worked into the EraSOR workflow?

>> We thank the reviewer for their comment. Our paper has two main goals: 1) To demonstrate the problem of sample overlap in the performance of PRS analyses; and 2) to introduce the EraSOR algorithm. As such, the 'type 1 error' simulations in the manuscript are included primarily to demonstrate the problem of sample overlap convincingly by showing that highly significant results are obtained under overlap, even under the null of no causal variants or heritability.

>> However, we have now explained this point in detail in the main text for clarity, so that it is absolutely clear to the reader that EraSOR should not be performed in relation to PRS analyses that use GWAS data with low heritability (h2SNP < 5%), and in fact we state in our 'guide to PRS' paper (Choi et al. 2020. Tutorial: a guide to polygenic risk score analyses') that PRS analyses should not be performed using GWAS with estimated h2SNP < 5%. Moreover, following the reviewer's suggestion, we have now expanded the pipeline of the EraSOR code to include a quality control step that estimates h2SNP in the base GWAS (provided by the user) and outputs a message to the reader stating that EraSOR is not performed in relation to base GWAS with h2SNP < 5%, since we do not recommend PRS analyses be performed if the base GWAS has estimated h2SNP < 5%.

Line 148 / expression (12): If beta has a normal distribution here, it is the case that all SNPs in the simulation are associated with the outcome Y. This is a somewhat unusual choice for the distribution of SNP effects in a simulation; other applications such as LDPred (Vilhjalmsson et al, AJHG 2015) and LassoSum (TSH Mak et al, Genetic Epi 2017) use a point-normal distribution for simulated SNP effects, which effectively simulates the sparsity frequently observed in nature. Is there a reference or justification for the non-sparse simulation structure here?

>> We thank the reviewer for their comment. In equation 12, we are in fact using a point-normal distribution for simulating SNP effects similar to that used in LDpred and lassosum. Here, only 10,000 SNPs have a non-zero effect size. We have now updated the manuscript to clarify this simulation.

Line 215: there may be a typo in the expression for the variance of the residual term. Is it the case that the variance of the residual depends on the variance of a covariance term? If so, I am confused as to the derivation.
>> We thank the reviewer for pointing this out. There was a typo in the residual term where $var(X\beta+S-2cov(X\beta,S))$ should have been included within the square root expression. We have now corrected this mistake.

Line 241: 'triat' should be 'trait'.

>> We thank the reviewer for spotting this error. We have now corrected this in the updated manuscript.

The simulation results in this paper are based on clumping and thresholding for PRS, which does not estimate joint SNP effects i.e. account for LD. Methods such as LDPred and LassoSum do so. Is there any reason to believe the results would be different for a method such as LassoSum?

>> We thank the reviewer for their comment. While methods such as LDPred and LassoSum do account for the correlation between SNPs in a statistically principled way, the ‘clumping' process of the standard PRS computation method does account for the correlation between the effects of different SNPs, albeit in a rather simplified way that acts only as a proxy for a full joint model of SNPs (see Choi et al. 2020. Nature Prot). Therefore, given this, and the relatively similar performance in trait prediction of all of these PRS calculation methods, then we do not believe that the results should be systematically different if using methods such as lassosum or LDpred. It is computationally prohibitive to perform our comprehensive simulations and benchmarking using methods such as lassosum or LDpred, but we have now discussed this point in the Discussion section and made clear that a limitation of our work is that we have not checked the impact of using EraSOR to correct PRS analyses with sample overlap performed using one of the multiple other PRS methods available (although we expect the results not to differ qualitatively since such methods will also be affected by sample overlap similarly and EraSOR is applied only to the base and target GWAS data, which are independent of any PRS analyses).

I am confused by the very low Fst between the simulated Finnish and Yoruban samples in simulation. As detailed on line 385: the reported Fst is > .1, but the simulated Fst is essentially zero. This seems likely to be an undesirable simulation artefact, and potentially invalidates the simulation study (or, at least, doesn't provide evidence that EraSOR functions correctly when Fst is large, which was the ostensible motivation for this simulation). Is there no way to effectively simulate populations with a larger Fst?

>> Unfortunately, after much time spent to try to resolve why HapGen-2 is not generating samples with large Fst in our analyses, and after contacting the HapGen-2 authors about the issue, we have not found a solution to this. Following the reviewer's point about this, we have decided to remove this HapGen-2 simulation study from the manuscript given the possibility that the results are subject to a simulation/coding artefact, and instead rely on the analyses performed in the UK Biobank in which we generate samples with an FST of 0.018 (European and non-European ancestry samples), which is relatively high compared to European-only samples.

>> In the Discussion we now explain that a limitation of our study is that we have only tested the performance of EraSOR in the real data of the UK Biobank, and so that we can only recommend the use of EraSOR in settings in which the base and target data are drawn from populations of the same or similar ancestry (at least no greater than random samples within the UK). However, the likely impact of this limitation is reduced by the fact that base and target samples that are drawn from different ancestral populations are substantially less likely to contain sample overlap in any case.

Reviewer #3: In this paper Choi et al. describe EraSOR, a new tool to remove the effects sample overlap between a set of summary statistics and a target dataset. EraSOR works by running a GWAS in the target dataset and then using LD-score regression techniques to estimate the heritability, genetic correlations of the phenotypes, and number of overlapping samples to decorrelate the effect sizes. The method is thoroughly described, and the simulation scenarios are relevant and well-motivated. However, the manuscript could better describe the inputs and characteristics of the decorrelated summary statistics, focusing more on the degree of bias in effect sizes rather than p-value inflation, and the practicalities of how the tool may be used.

>> We thank the reviewer for their helpful comments, which have helped us to produce, we believe, a substantially improved manuscript.

Specific Comments:
* The results of Figure 1/Supp Figure 1 are highly motivating, but the p-value of the association doesn't seem like the perfect measure of inflation. Plots of the effect size of the PRS compared to its expected effect (0, based on heritability) would better illustrate this.

>> We thank the reviewer for their comment. The main purpose of Figure 1 is to show the effect of sample overlap on the increase of false positive results and so feel that this is made clearest through a focus on the impact on P-values rather than change in effect size estimates. We did consider specifically highlighting false-positive rates rather than P-values but the false-positive rates are mostly 100% even with limited overlap and so we decided to illustrate the impact on P-values directly to retain greater information and differentiation between scenarios (e.g. different target sample sizes).

* The paper proposes a method to remove the effects of sample overlap on summary statistics, but instead mostly focuses on how overlap biases the results of PRS prediction. Additional exploration of the decorrelated summary statistics themselves is needed to illustrate the validity of the method. Specifically, how different are the EraSOR adjusted summary statistics from the true summary statistics measured without sample overlap (e.g. distribution of effect sizes differences); what types of variants does EraSOR fail for or overcorrect (e.g. MAF differences between the summary statistics and the target cohort)? Are the results used as-is in other analyses, or do they have to be filtered in some way?

>> We thank the reviewer for their comments. The main motivation of the current paper is to specifically tackle the problem of sample overlap in PRS analysis. There are many algorithms designed that account for sample overlap in the context of other genetic studies, for example, METAL has an algorithm for meta-analysis; MTAG is designed to account for sample overlap in cross-trait GWAS analyses; whereas LDSC and LDAK account for sample overlap in heritability estimation. To our knowledge, EraSOR is the only algorithm that can account for an unknown degree of sample overlap in PRS analyses. As such, the main focus of the current paper is its utility for PRS analyses, which we believe should be highly impactful given the large number of PRS analyses being published presently, almost all of which have unknown sample overlap and thus potentially questionable results and conclusions. Our results present extensively on how EraSOR performs in terms of PRS-trait associations across a range of scenarios, each compared to associations observed with sample overlap removed. However, we agree with the reviewer that the statistical development of EraSOR could be useful in other settings, which we think will make our paper an important starting point not only for PRS applied studies but also for methodological follow-up in other settings.

>> In terms of whether results need post-EraSOR filtering – no, the EraSOR corrected GWAS results can be used as-is, albeit the use of EraSOR assumes that the standard GWAS quality control and filtering processes have already been applied to both the base and target data (as recommended in our 'PRS guide paper': ref.1). We have now made this clear in the manuscript and in our new detailed documentation of EraSOR that we produced in response to this and subsequent points from the reviewer (https://choishingwan.gitlab.io/EraSOR/).

* The PRS analyses in the paper all use PRSice to perform clumping+thresholding, selecting the best p-value and LD thresholds on the target datasets. This could be considered overfitting to the target data, and other derivation methods that do not require a sample to optimize hyperparameters (e.g. PRS-cs, LDpred-

auto) could be used. It would be good to provide some additional analyses showing that EraSOR outputs also work with other methods of PRS derivation, and that the results are not sensitive to overfitting through hyperparameter optimization.

>> We thank the reviewer for their comment. We acknowledge that the results from the C+T analyses may be subject to overfitting and we have now highlighted this in the Discussion. However, the problem of overfitting, and thus PRS calculation accuracy, is independent of inflation caused by sample overlap. Therefore, the use of the EraSOR adjustment, and thus the difference between adjusted, unadjusted and observed results, should not be sensitive to overfitting (or underfitting) or the specific PRS calculation method used. We opted to use the C+T approach, using our PRSice implementation, because it remains the default and most popular PRS calculation method and because our PRSice software is highly computationally efficient, allowing us to perform a huge range of scenarios to evaluate the performance of EraSOR in different settings, which would not have been possible had alternative PRS methods also been included.

* The PRS analysis of the real phenotype data in UKB should be expanded. Currently the analysis uses summary statistics derived in UKB with varying levels of overlap; however, this does not match the real scenario that EraSOR will likely be used in (applying EraSOR to an externally-sourced GWAS and applied to UK Biobank). The authors should perform a descriptive analysis to show that EraSOR is useful in this real-world scenario by downloading summary statistics from the GWAS Catalog (with and without inclusion of UK Biobank), applying EraSOR, and quantifying the difference in accuracy (r2) and effect size. On a related note: does the ancestry of the summary statistics have to perfectly match the target cohort? How well does EraSOR work with multi-ancestry summary statistics where the LD-panel might be mismatched?

>> The main reason why externally sourced GWAS were not used in the current analyses is that without access to the raw genotyped data, we cannot establish the empirical truth and would not know whether results generated by EraSOR reflect scenarios where the sample overlap is known. Limited by data availability, we can only perform large-scale real data analyses using UK Biobank data where we can estimate the expected R2 and effect size of the PRS association according to retaining and removing known sample overlap to assess the performance of EraSOR.

>> In the Discussion, we now make clear that EraSOR has only been tested in settings of ancestry-matched target data, whereby both base and target data sets are predominantly single ancestry (see final response to reviewer 2), and so can only recommend its use in these settings presently.

* The point about insufficient adjustment the authors raise on lines 336-42 is quite important. Proper signposting about the limits of the decorrelation is needed in the software description and the discussion. From this passage that the authors suggest that known sample overlap should be avoided and EraSOR should only be used as a sensitivity analysis to ensure that overlap does not exist? It would be useful to get the authors perspective on whether the evaluation of a PRS in a cohort derived using EraSOR-adjusted summary statistics can be seen as truly external to the source GWAS.

>> We thank the reviewer for their comment. The issue raised on lines 336-42 is specific to the scenario where *all samples* from the target were found in the base data. In this scenario, the EraSOR adjustment is unlikely to be optimal because of the complete degree of overlap, but will still generate results much closer to the empirical truth when compared to the unadjusted results, making this an ideal sensitivity analyses. In this scenario, EraSOR-adjusted summary statistics cannot be viewed as truly “free” of overlap – in the same way as imputed genotypes cannot be viewed as the same as true genotypes (but are useful estimates of them) – and deviation of EraSOR adjusted results and the unadjusted results should be treated as a possible red flag for analysts, informing them about the possibility of overlapping samples between their base and target data. In this case, they should reach out to the GWAS cohort to request leave-one-out GWAS results for their PRS analyses to achieve perfect non-overlap between base and target data, which is always preferable to a mathematical approximation to non-overlapping data. We have now expanded our limitation section to better describe this scenario and our perspective on it.

* The paper should be accompanied by a more detailed user guide and some test data for the EraSOR tool.

Are there any diagnostic plots that are produced that could be used to inspect the data quality?

>> We thank the reviewer for their comment. We have included a detailed documentation, including diagnostic plots, in https://choishingwan.gitlab.io/EraSOR/ and have included test data for users to familiarise themselves with the use of EraSOR.

Close
