# Supplementary material for: EraSOR: a software tool to eliminate inflation caused by sample overlap in polygenic score analyses
Source: Gigascience. 2023 Jun 16;12:giad043. doi: 10.1093/gigascience/giad043 (PMC10273836; doi:10.1093/gigascience/giad043)
Supplement: giad043_Response_to_Reviewer_Comments_Revision_1 [file giad043_response_to_reviewer_comments_revision_1.pdf]

Author's Response To Reviewer Comments

Close

Reviewer #2: We thank the reviewer for their time and effort.
Reviewer #3:
The revised manuscript is much clearer and better illustrates when and how to use the EraSOR method. However, I still think important analyses reflecting more common use cases are missing:
We thank the reviewer for their suggestions and comments. Below are our point-by-point response.

1. Use of EraSOR with multi-ancestry summary statistics

We thank the reviewer for this suggestion. We agree that the use of multi-ancestry summary statistics is becoming more standard (as it should be) in GWAS and polygenic risk score research and would ideally be tested in the current context. However, an important assumption from LD score regression, which we have used directly in our EraSOR method, is that the level of genetic and environmental stratification is similar between the two study cohorts (line 469 - 471). Therefore, according to the recommendation of LD score regression, our method is not recommended in the context of multi-ancestry summary statistics for which it is not possible to match ancestry with target data. This was highlighted as one of the limitations of EraSOR in the discussion (line 471 – 472) and is explicitly advised against (line 516-519). To further illustrate this, we have now performed an analysis using the GLGC multi-ancestry summary statistics to illustrate the problem:

"To further evaluate the performance of EraSOR in real data settings, we used the latest GLGC LDL summary statistics and the LDL GWAS data from the UK Biobank. Using the European-specific GLGC summary statistics, where the UK Biobank samples were removed, the expected PRS-R2 was 0.102. When the GWAS summary statistics contain the UK Biobank samples, the PRS-R2 is 0.205, almost double the value without sample overlap. However, this inflation was alleviated when we used the EraSOR-adjusted summary statistics, which yielded a PRS-R2 of 0.0918, which represents a relative decrease of 10% compared to the overlap-removed PRS-R2, consistent with our simulation results.
We note that EraSOR relies on the intercept term from LD score regression, the estimate of which assumes that the level of genetic and environmental stratification is similar between the base and target cohorts [13]. When we used the trans-ancestry GLGC GWAS summary statistics, the PRS-R2 with the overlapping samples (UK Biobank) removed was 0.145, the PRS-R2 using the GWAS that included overlapping samples was 0.228, representing a relative increase of 57%. Although we did not observe the same inflation with the EraSOR-adjusted summary statistics, which yielded a PRS-R2 of 0.0962, its performance was less accurate compared to the case where base and target ancestries were relatively matched, with a relative PRS-R2 decrease of 33.5%, suggesting that EraSOR might be overly-conservative in such settings."

And we have also rephrased the limitation section to better convey this:
"Our algorithm is not without limitation. First, as EraSOR relies on LD score intercept estimates for adjustment, all assumptions of LD score regression also apply to EraSOR. For instance, if the level of genetic and environmental stratification differs between the two cohorts, the bivariate LD score equation may not hold [13], which can lead to bias in EraSOR estimates."

2. Use of EraSOR corrected sumstats with other PGS-derivation methods (e.g. LDpred or PRS-CS).

We thank the reviewer for this suggestion. We have now performed an additional analysis using lassosum (see supplementary) and show that EraSOR corrected summary statistics still eliminate the inflation of lassosum results that include sample overlap between base and target samples. We did not perform the analysis on other PRS software due to the high computational burden and the fact that lassosum performs a very different PRS computation method to PRSice, and more similar to many other methods, such as LDpred and PRS-CSx, and so we can reasonably assume that the performance of EraSOR generalizes well

to many PRS computation methods. The results are now presented as Supplementary Figure 3.

3. Providing results of a real sensitivity analysis for sample overlap. I understand that you won't know the true overlap in UKB but the difference in the adjusted and unadjusted SumStats performance in the presence of known overlap would be illustrative. Adding these analyses to the real UKB section would greatly benefit the manuscript and utility of the method.

We thank the reviewer for this comment. Unfortunately, unless we have access to consortia data providing summary statistics with the UK Biobank removed, it is impossible for us to know the true overlap in real data settings and, thus, assess the performance of EraSOR. Fortunately, GLGC has recently published their latest GWAS, containing both the UK Biobank left out and whole consortium summary statistics, which allow us to perform a sensitivity analysis. Therefore, we have conducted analyses using these data, which show that EraSOR still performs well (see response to point 1 above). As such, we anticipate that EraSOR should be an appropriate tool for this kind of sensitivity analyses in real world settings, accounting appropriately for the degree of ancestry matching between base and target data (as described in response ot point 1 above, and the text in the manuscript highlighted therein)

Minor:
Apart from that I note that related to line 19, the impact of sample overlap was also outlined as a pitfall by Wray et al Nat Genet (2013, PMID:23774735).
We thank the reviewer for this comment. Line 19 is part of the abstract and we do not think a citation is required. However, we have cited Professor Wray's paper in our introduction (line 44, citation 15) when we introduce the problems of sample overlap in polygenic risk score research.

Close
