# Supplementary material for: EraSOR: a software tool to eliminate inflation caused by sample overlap in polygenic score analyses
Source: Gigascience. 2023 Jun 16;12:giad043. doi: 10.1093/gigascience/giad043 (PMC10273836; doi:10.1093/gigascience/giad043)
Supplement: giad043_Response_to_Reviewer_Comments_Revision_2 [file giad043_response_to_reviewer_comments_revision_2.pdf]

Author's Response To Reviewer Comments

Close

Attached are the updated manuscript with the required correction to citations. Thank you.

Close
