# Supplementary material for: EraSOR: a software tool to eliminate inflation caused by sample overlap in polygenic score analyses
Source: Gigascience. 2023 Jun 16;12:giad043. doi: 10.1093/gigascience/giad043 (PMC10273836; doi:10.1093/gigascience/giad043)
Supplement: giad043_Reviewer_1_Report_Original_Submission — Christopher C. Chang, Ph.D. -- 2/14/2022 Reviewed [file giad043_reviewer_1_report_original_submission.pdf]

Reviewer Report

Title: EraSOR: a software tool to eliminate inflation caused by sample overlap in polygenic score analyses

Version: Original Submission **Date:** 2/14/2022

Reviewer name: Christopher C. Chang, Ph.D.

Reviewer Comments to Author:

This paper addresses a significant need that has arisen in the interaction between privacy rules and ever-larger genomic datasets, and I find the results to be very promising and clearly worth publishing. I just have a few comments on some methodological details:line 130: Have you compared the effectiveness of this algorithm with plink2 --king-cutoff?lines 145-155: If I understand this correctly, these simulated quantitative traits are still normally distributed, they just aren't standardized to mean 0 variance 1. If the intent is to "simulate phenotypes that [do] not follow the standard normal distribution", I'd expect it to be more valuable to look at e.g. the log-normal case, where an alert user might transform the phenotype to normal, but some users may fail to do so. A mixture distribution may also be worth looking at.lines 238-239: Have you considered using the "cc-residualize" option of plink2 --glm, which removes most of the computational cost of including PCs in your binary trait analysis?lines 383-387: This is interesting; there is some room for follow-up investigation here. Thanks for posting all the scripts needed for another researcher to easily reproduce this Fst=0.00639 value; this could help facilitate development of a better genotype-simulation tool.Also, some minor copyedits:line 84: "subpopulation" -> "subpopulations"line 342: "overlaps" -> "overlap"line 363: "ErasOR" -> "EraSOR"line 376: "different level of environmental stratifications" -> "different levels of environmental stratification"line 384: "population" -> "populations"line 402: "capture" -> "captured"

Level of Interest

Please indicate how interesting you found the manuscript: Choose an item.

Quality of Written English

Please indicate the quality of language in the manuscript: Choose an item.

Declaration of Competing Interests

Please complete a declaration of competing interests, considering the following questions:

- Have you in the past five years received reimbursements, fees, funding, or salary from an organisation that may in any way gain or lose financially from the publication of this manuscript, either now or in the future?

- Do you hold any stocks or shares in an organisation that may in any way gain or lose financially from the publication of this manuscript, either now or in the future?
- Do you hold or are you currently applying for any patents relating to the content of the manuscript?
- Have you received reimbursements, fees, funding, or salary from an organization that holds or has applied for patents relating to the content of the manuscript?
- Do you have any other financial competing interests?
- Do you have any non-financial competing interests in relation to this paper?

If you can answer no to all of the above, write 'I declare that I have no competing interests' below. If your reply is yes to any, please give details below.

I declare that I have no competing interests

I agree to the open peer review policy of the journal. I understand that my name will be included on my report to the authors and, if the manuscript is accepted for publication, my named report including any attachments I upload will be posted on the website along with the authors' responses. I agree for my report to be made available under an Open Access Creative Commons CC-BY license (http://creativecommons.org/licenses/by/4.0/). I understand that any comments which I do not wish to be included in my named report can be included as confidential comments to the editors, which will not be published.

Choose an item.

To further support our reviewers, we have joined with Publons, where you can gain additional credit to further highlight your hard work (see: https://publons.com/journal/530/gigascience). On publication of this paper, your review will be automatically added to Publons, you can then choose whether or not to claim your Publons credit. I understand this statement.

Yes Choose an item.
