# Supplementary material for: EraSOR: a software tool to eliminate inflation caused by sample overlap in polygenic score analyses
Source: Gigascience. 2023 Jun 16;12:giad043. doi: 10.1093/gigascience/giad043 (PMC10273836; doi:10.1093/gigascience/giad043)
Supplement: giad043_Reviewer_2_Report_Original_Submission — Jack Pattee, Ph.D. -- 2/21/2022 Reviewed [file giad043_reviewer_2_report_original_submission.pdf]

Reviewer Report

Title: EraSOR: a software tool to eliminate inflation caused by sample overlap in polygenic score analyses

Version: Original Submission **Date:** 2/21/2022

Reviewer name: Jack Pattee, Ph.D.

Reviewer Comments to Author:

Overall, I think that this manuscript is strong and describes a well-formulated method to address a relevant problem. There are a few outstanding questions about the performance of the EraSOR method from my perspective, which I'll detail as follows.My understanding of reference [16] indicates that equation (3) of this manuscript only holds for null SNPs, i.e. if SNP g is not associated with the outcome Y. If this is the case, then this should be discussed in the manuscript. I wonder if this can partially explain the 'under-estimation' behavior we see in the application to real data in Supplementary Figure 3. In particular, I am referencing the behavior where the EraSOR correction will under-estimate the predictive accuracy of the PRS in the target data, i.e. where delta-R^2 is negative. This behavior is not seen in the simulation and warrants further investigation and discussion. While the bias appears small, for some cases delta-R^2 approaches -.025, which corresponds to an under-estimation of Pearson's r by roughly .15; this is substantial. Could it be the case that, for highly polygenic traits such as height and BMI, the null-SNP assumption is unreliable and the performance of EraSOR is degraded? Does a fundamental assumption of sparse genetic association underlie EraSOR?I recommend that the real data application play a larger role in the manuscript narrative and be moved out of the supplementary. The simulations are appreciated and helpful, but there is nuance in the analysis of real data that cannot be replicated in simulation.I believe the reference to "Supplementary Figure 2" on line 346 should actually be "Supplementary Figure 3". I believe that the axis labels in Supp Figure 3 are flipped.Lines 82 and 83 reference genetic stratification and subpopulations; I think the relevance of these concepts should be introduced more clearly and they should be defined in this context. EraSOR concerns the over-estimation of predictive accuracy and association incurred by sample overlap between the base and target GWASs; to this reader, it's not clear what this central issue has to do with population stratification. I realize that the derivation of the LD score method is motivated heavily by correcting for stratification; however, these concepts should be introduced more clearly in this manuscript.Line 88: consider defining LD score l_j.Lines 94-96: consider outlining the mathematical consequence of the assumption that "the two outcomes and cohorts are identical." It's the case that N_1 = N_2 = N_c = N, correct?Line 109 / equation (11): My understanding is that the relevant quantity of this derivation is N_c / sqrt(N_1 N_2), which allows us to define the correct matrix C in expression (4). If this is the case, perhaps the quantity of interest should be moved to the LHS of the equation in the final line of the expression, for clarity.As discussed in the manuscript, the estimated heritability is in the denominator of the expression for N_c / sqrt(N_1 N_2). The authors correctly discuss that the method should not be applied when there is doubt as to whether the heritability is different from zero. I would take this a step further; in cases where the heritability is zero, we cannot meaningfully apply the EraSOR correction, and

thus I am not sure of the utility of the 'type I error' simulations in the manuscript. Perhaps an explicit test for h^2 > 0 should be worked into the EraSOR workflow?Line 148 / expression (12): If beta has a normal distribution here, it is the case that all SNPs in the simulation are associated with the outcome Y. This is a somewhat unusual choice for the distribution of SNP effects in a simulation; other applications such as LDPred (Vilhjalmsson et al, AJHG 2015) and LassoSum (TSH Mak et al, Genetic Epi 2017) use a point-normal distribution for simulated SNP effects, which effectively simulates the sparsity frequently observed in nature. Is there a reference or justification for the non-sparse simulation structure here?Line 215: there may be a typo in the expression for the variance of the residual term. Is it the case that the variance of the residual depends on the variance of a covariance term? If so, I am confused as to the derivation.Line 241: 'triat' should be 'trait'.The simulation results in this paper are based on clumping and thresholding for PRS, which does not estimate joint SNP effects i.e. account for LD. Methods such as LDPred and LassoSum do so. Is there any reason to believe the results would be different for a method such as LassoSum?I am confused by the very low Fst between the simulated Finnish and Yoruban samples in simulation. As detailed on line 385: the reported Fst is > .1, but the simulated Fst is essentially zero. This seems likely to be an undesirable simulation artefact, and potentially invalidates the simulation study (or, at least, doesn't provide evidence that EraSOR functions correctly when Fst is large, which was the ostensible motivation for this simulation). Is there no way to effectively simulate populations with a larger Fst?

Level of Interest

Please indicate how interesting you found the manuscript: Choose an item.

Quality of Written English

Please indicate the quality of language in the manuscript: Choose an item.

Declaration of Competing Interests

Please complete a declaration of competing interests, considering the following questions:

- Have you in the past five years received reimbursements, fees, funding, or salary from an organisation that may in any way gain or lose financially from the publication of this manuscript, either now or in the future?
- Do you hold any stocks or shares in an organisation that may in any way gain or lose financially from the publication of this manuscript, either now or in the future?
- Do you hold or are you currently applying for any patents relating to the content of the manuscript?
- Have you received reimbursements, fees, funding, or salary from an organization that holds or has applied for patents relating to the content of the manuscript?
- Do you have any other financial competing interests?
- Do you have any non-financial competing interests in relation to this paper?

If you can answer no to all of the above, write 'I declare that I have no competing interests' below. If your reply is yes to any, please give details below.

I declare that I have no competing interests.

I agree to the open peer review policy of the journal. I understand that my name will be included on my report to the authors and, if the manuscript is accepted for publication, my named report including any attachments I upload will be posted on the website along with the authors' responses. I agree for my report to be made available under an Open Access Creative Commons CC-BY license (http://creativecommons.org/licenses/by/4.0/). I understand that any comments which I do not wish to be included in my named report can be included as confidential comments to the editors, which will not be published.

Choose an item.

To further support our reviewers, we have joined with Publons, where you can gain additional credit to further highlight your hard work (see: https://publons.com/journal/530/gigascience). On publication of this paper, your review will be automatically added to Publons, you can then choose whether or not to claim your Publons credit. I understand this statement.

Yes Choose an item.
