# Supplementary material for: EraSOR: a software tool to eliminate inflation caused by sample overlap in polygenic score analyses
Source: Gigascience. 2023 Jun 16;12:giad043. doi: 10.1093/gigascience/giad043 (PMC10273836; doi:10.1093/gigascience/giad043)
Supplement: giad043_Reviewer_3_Report_Original_Submission — Samuel A. Lambert -- 2/28/2022 Reviewed [file giad043_reviewer_3_report_original_submission.pdf]

Reviewer Report

Title: EraSOR: a software tool to eliminate inflation caused by sample overlap in polygenic score analyses

Version: Original Submission **Date:** 2/28/2022

Reviewer name: Samuel A. Lambert

Reviewer Comments to Author:

In this paper Choi et al. describe EraSOR, a new tool to remove the effects sample overlap between a set of summary statistics and a target dataset. EraSOR works by running a GWAS in the target dataset and then using LD-score regression techniques to estimate the heritability, genetic correlations of the phenotypes, and number of overlapping samples to decorrelate the effect sizes. The method is thoroughly described, and the simulation scenarios are relevant and well-motivated. However, the manuscript could better describe the inputs and characteristics of the decorrelated summary statistics, focusing more on the degree of bias in effect sizes rather than p-value inflation, and the practicalities of how the tool may be used.Specific Comments:* The results of Figure 1/Supp Figure 1 are highly motivating, but the p-value of the association doesn't seem like the perfect measure of inflation. Plots of the effect size of the PRS compared to its expected effect (0, based on heritability) would better illustrate this.* The paper proposes a method to remove the effects of sample overlap on summary statistics, but instead mostly focuses on how overlap biases the results of PRS prediction. Additional exploration of the decorrelated summary statistics themselves is needed to illustrate the validity of the method. Specifically, how different are the EraSOR adjusted summary statistics from the true summary statistics measured without sample overlap (e.g. distribution of effect sizes differences); what types of variants does EraSOR fail for or overcorrect (e.g. MAF differences between the summary statistics and the target cohort)? Are the results used as-is in other analyses, or do they have to be filtered in some way?* The PRS analyses in the paper all use PRSice to perform clumping+thresholding, selecting the best p-value and LD thresholds on the target datasets. This could be considered overfitting to the target data, and other derivation methods that do not require a sample to optimize hyperparameters (e.g. PRS-cs, LDpred-auto) could be used. It would be good to provide some additional analyses showing that EraSOR outputs also work with other methods of PRS derivation, and that the results are not sensitive to overfitting through hyperparameter optimization.* The PRS analysis of the real phenotype data in UKB should be expanded. Currently the analysis uses summary statistics derived in UKB with varying levels of overlap; however, this does not match the real scenario that EraSOR will likely be used in (applying EraSOR to an externally-sourced GWAS and applied to UK Biobank). The authors should perform a descriptive analysis to show that EraSOR is useful in this real-world scenario by downloading summary statistics from the GWAS Catalog (with and without inclusion of UK Biobank), applying EraSOR, and quantifying the difference in accuracy (r2) and effect size. On a related note: does the ancestry of the summary statistics have to perfectly match the target cohort? How well does EraSOR work with multi-ancestry summary statistics where the LD-panel might be mismatched?* The point about insufficient adjustment the authors raise on lines 336-42 is quite important. Proper signposting about the limits of

the decorrelation is needed in the software description and the discussion. From this passage that the authors suggest that known sample overlap should be avoided and EraSOR should only be used as a sensitivity analysis to ensure that overlap does not exist? It would be useful to get the authors perspective on whether the evaluation of a PRS in a cohort derived using EraSOR-adjusted summary statistics can be seen as truly external to the source GWAS.* The paper should be accompanied by a more detailed user guide and some test data for the EraSOR tool. Are there any diagnostic plots that are produced that could be used to inspect the data quality?

Level of Interest

Please indicate how interesting you found the manuscript: Choose an item.

Quality of Written English

Please indicate the quality of language in the manuscript: Choose an item.

Declaration of Competing Interests

Please complete a declaration of competing interests, considering the following questions:

- Have you in the past five years received reimbursements, fees, funding, or salary from an organisation that may in any way gain or lose financially from the publication of this manuscript, either now or in the future?
- Do you hold any stocks or shares in an organisation that may in any way gain or lose financially from the publication of this manuscript, either now or in the future?
- Do you hold or are you currently applying for any patents relating to the content of the manuscript?
- Have you received reimbursements, fees, funding, or salary from an organization that holds or has applied for patents relating to the content of the manuscript?
- Do you have any other financial competing interests?
- Do you have any non-financial competing interests in relation to this paper?

If you can answer no to all of the above, write 'I declare that I have no competing interests' below. If your reply is yes to any, please give details below.

I declare that I have no competing interests

I agree to the open peer review policy of the journal. I understand that my name will be included on my report to the authors and, if the manuscript is accepted for publication, my named report including any attachments I upload will be posted on the website along with the authors' responses. I agree for my report to be made available under an Open Access Creative Commons CC-BY license (http://creativecommons.org/licenses/by/4.0/). I understand that any comments which I do not wish to

be included in my named report can be included as confidential comments to the editors, which will not be published.

Choose an item.

To further support our reviewers, we have joined with Publons, where you can gain additional credit to further highlight your hard work (see: https://publons.com/journal/530/gigascience). On publication of this paper, your review will be automatically added to Publons, you can then choose whether or not to claim your Publons credit. I understand this statement.

Yes Choose an item.
